# Proceedings of the Frontiers of Retrovirology Conference 2016

**DOI:** 10.1186/s12977-016-0294-5

**Published:** 2016-09-26

**Authors:** Irena Zurnic, Sylvia Hütter, Ute Lehmann, Nicole Stanke, Juliane Reh, Tobias Kern, Fabian Lindel, Gesche Gerresheim, Martin Hamann, Erik Müllers, Paul Lesbats, Peter Cherepanov, Erik Serrao, Alan Engelman, Dirk Lindemann, Claire Da Silva Santos, Kevin Tartour, Andrea Cimarelli, Rya Burdick, Jianbo Chen, Jaya Sastri, Wei-Shau Hu, Vinay Pathak, Oliver T. Keppler, Karine Pradeau, Sylvia Eiler, Nicolas Levy, Sarah Lennon, Sarah Cianferani, Stéphane Emiliani, Marc Ruff, Vincent Parissi, Sylvie Rato, Antonio Rausell, Miguel Munoz, Amalio Telenti, Angela Ciuffi, Alexander Zhyvoloup, Anat Melamed, Ian Anderson, Delphine Planas, Janos Kriston-Vizi, Robin Ketteler, Chen- Hsuin Lee, Andy Merritt, Petronela Ancuta, Charles Bangham, Ariberto Fassati, Anthony Rodari, Benoit Van Driessche, Mathilde Galais, Nadége Delacourt, Sylvain Fauquenoy, Caroline Vanhulle, Anna Kula, Arsène Burny, Olivier Rohr, Carine Van Lint, Thijs van Montfort, Renee van der Sluis, Dave Speijer, Ben Berkhout, Bo Meng, Andrzej Rutkowski, Neil Berry, Lars Dölken, Andrew Lever, Thomas Schuster, Benedikt Asbach, Ralf Wagner, Christine Gross, Veit Wiesmann, Martina Kalmer, Thomas Wittenberg, Jan Gettemans, Andrea K. Thoma-Kress, Minghua Li, Eric O. Freed, Shan-Lu Liu, Janis Müller, Jan Münch, Xaver Sewald, Pradeep Uchil, Mark Ladinsky, Jagadish Beloor, Ruoxi Pi, Christin Herrmann, Nasim Motamedi, Thomas Murooka, Michael Brehm, Dale Greiner, Thorsten Mempel, Pamela Bjorkman, Priti Kumar, Walther Mothes, Simone Joas, Erica Parrish, Clement Wesley Gnanadurai, Edina Lump, Christina M. Stürzel, Nicholas F. Parrish, Ulrike Sauermann, Katharina Töpfer, Tina Schultheiss, Steven Bosinger, Guido Silvestri, Cristian Apetrei, Nicholas Huot, Michaela Müller-Trutwin, Daniel Sauter, Beatrice H. Hahn, Christiane Stahl-Hennig, Frank Kirchhoff, Gerald Schumann, Sabine Jung-Klawitter, Nina V. Fuchs, Kyle R. Upton, Martin Muñoz-Lopez, Ruchi Shukla, Jichang Wang, Marta Garcia-Canadas, Cesar Lopez-Ruiz, Daniel J. Gerhardt, Attila Sebe, Ivana Grabundzija, Patricia Gerdes, Sylvia Merkert, Andres Pulgarin, Anja Bock, Ulrike Held, Anett Witthuhn, Alexandra Haase, Ernst J. Wolvetang, Ulrich Martin, Zoltán Ivics, Zsuzsanna Izsvák, J. Garcia-Perez, Geoffrey J. Faulkner, Tara Hurst, Aris Katzourakis, Gkikas Magiorkinis, Kerstin Schott, Rita Derua, Janna Seifried, Andreas Reuter, Heike Schmitz, Christiane Tondera, Alberto Brandariz-Nuñez, Felipe Diaz-Griffero, Veerle Janssens, Renate König, Hanna-Mari Baldauf, Lena Stegmann, Sarah-Marie Schwarz, Maud Trotard, Margarethe Martin, Gina Lenzi, Manja Burggraf, Xiaoyu Pan, Oliver I. Fregoso, Efrem S. Lim, Libin Abraham, Elina Erikson, Laura Nguyen, Ina Ambiel, Frank Rutsch, Baek Kim, Michael Emerman, Oliver T. Fackler, Sabine Wittmann, Rayk Behrendt, Bianca Volkmann, Kristin Eissmann, Thomas Gramberg, Sebastian Bolduan, Herwig Koppensteiner, Stefanie Regensburg, Ruth Brack-Werner, Rika Draenert, Michael Schindler, Aurélie Ducroux, Shuting Xu, Aparna Ponnurangam, Sergej Franz, Angelina Malassa, Ellen Ewald, Christine Goffinet, Sin-Yee Fung, Ching-Ping Chan, Chun-Kit Yuen, Kin-Hang Kok, Chin-Ping Chan, Dong-Yan Jin, Ulf Dittmer, Dorota Kmiec, Shilpa Iyer, Christina Stürzel, Beatrice Hahn, Yasuo Ariumi, Mariko Yasuda-Inoue, Koudai Kawano, Satoshi Tateishi, Priscilla Turelli, Alex Compton, Nicolas Roy, Françoise Porrot, Anne Billet, Nicoletta Casartelli, Jacob Yount, Chen Liang, Oliver Schwartz, Carsten Magnus, Lucia Reh, Penny Moore, Therese Uhr, Jacqueline Weber, Lynn Morris, Alexandra Trkola, Rashel V. Grindberg, Erika Schlaepfer, Gideon Schreiber, Viviana Simon, Roberto F. Speck, Zeger Debyser, Lenard Vranckx, Jonas Demeulemeester, Suha Saleh, Eric Verdin, Anna Cereseto, Frauke Christ, Rik Gijsbers, Gang Wang, Na Zhao, Atze T. Das, Josef Köstler, Beatriz Perdiguero, Mariano Esteban, Bertram L. Jacobs, David C. Montefiori, Celia C. LaBranche, Nicole L. Yates, Georgia D. Tomaras, Guido Ferrari, Kathryn E. Foulds, Mario Roederer, Gary Landucci, Donald N. Forthal, Michael S. Seaman, Natalie Hawkins, Steven G. Self, Sanjay Phogat, James Tartaglia, Susan W. Barnett, Brian Burke, Anthony D. Cristillo, Song Ding, Jonathan L. Heeney, Giuseppe Pantaleo, Viktoria Stab, Armin Ensser, Bettina Tippler, Dennis Burton, Matthias Tenbusch, Klaus Überla, Galit Alter, Giuseppe Lofano, Anne-Sophie Dugast, Viraj Kulkarni, Todd Suscovich, Tatiana Opazo, Felipe Barraza, Diego Herrera, Andrea Garces, Tomas Schwenke, Diego Tapia, Jorge Cancino, Gloria Arriagada, Christina Haußner, Dominik Damm, Anette Rohrhofer, Barbara Schmidt, Jutta Eichler, Rebecca Midgley, James Wheeldon, Vincent Piguet, Priyanka Khopkar, Megha Rohamare, Smita Kulkarni, Ana Godinho-Santos, Allan Hance, Joao Goncalves, Fabrizio Mammano, Romain Gasser, Meriem Hamoudi, Martina Pellicciotta, Zhicheng Zhou, Clara Visdeloup, Philippe Colin, Martine Braibant, Bernard Lagane, Matteo Negroni, Jula Wamara, Norbert Bannert, Thibault Mesplede, Nathan Osman, Kaitlin Anstett, Jiaming Calvin Liang, Hanh Thi Pham, Mark Wainberg, Wei Shao, Jigui Shan, Mary Kearney, Xiaolin Wu, Frank Maldarelli, John Mellors, Brian Luke, John Coffin, Stephen Hughes, Thomas Fricke, Silvana Opp, Caitlin Shepard, Dmitri Ivanov, Jose Valle-Casuso, Marine Kanja, Pierre Cappy, Matteo Negroni, Daniela Lener, Ekaterina Knyazhanskaya, Andrey Anisenko, Timofey Zatsepin, Marina Gottikh, Alexander Komkov, Anastasia Minervina, Gaiaz Nugmanov, Vadim Nazarov, Konstantin Khodosevich, Ilgar Mamedov, Yuri Lebedev, Marta Colomer-Lluch, Ruth Serra-Moreno, Ambra Sarracino, Lavina Gharu, Alexander Pasternak, Alessandro Marcello, Ann Marie McCartin, Anurag Kulkarni, Valentin Le Douce, Virginie Gautier, Ann Baeyens, Evelien Naessens, Anouk Van Nuffel, Karin Weening, Anne- Marie Reilly, Eva Claeys, Wim Trypsteen, Linos Vandekerckhove, Sven Eyckerman, Kris Gevaert, Bruno Verhasselt, Hoi Ping Mok, Nicholas Norton, Axel Fun, Jack Hirst, Mark Wills, Dalibor Miklik, Filip Senigl, Jiri Hejnar, Jun-ichi Sakuragi, Sayuri Sakuragi, Masaru Yokoyama, Tatsuo Shioda, Hironori Sato, Jochen Bodem, Rebecca Moschall, Sarah Denk, Steffen Erkelenz, Christian Schenk, Heiner Schaal, Norbert Donhauser, Ellen Socher, Sebastian Millen, Heinrich Sticht, Christine Gross, Melanie Mann, Guochao Wei, Matthew J. Betts, Yang Liu, Timo Kehl, Robert B. Russell, Martin Löchelt, Oliver Hohn, Saeed Mostafa, Kirsten Hanke, Stephen Norley, Chia-Yen Chen, Masashi Shingai, Pedro Borrego, Nuno Taveira, Klaus Strebel, Chris Hellmund, Bo Meng, Melanie Friedrich, Friedrich Hahn, Christian Setz, Pia Rauch, Kirsten Fraedrich, Alina Matthaei, Petra Henklein, Maximilian Traxdorf, Torgils Fossen, Ulrich Schubert, Aya Khwaja, Meytal Galilee, Akram Alian, Birco Schwalbe, Heiko Hauser, Michael Schreiber, Mirte Scherpenisse, Young-Keol Cho, Jungeun Kim, Daeun Jeong, Katerina Trejbalova, Martina Benesova, Dana Kucerova, Zdenka Vernerova, Rachel Amouroux, Petra Hajkova, Daniel Elleder, Tomas Hron, Helena Farkasova, Abinash Padhi, Jan Paces, Henan Zhu, Robert Gifford, Pablo Murcia, Maria Luisa Carrozza, Anna-Maria Niewiadomska, Maurizio Mazzei, Mounir Abi-Said, Joseph Hughes, Stéphane Hué, Robert Gifford, Adetayo Obasa, Graeme Jacobs, Susan Engelbrecht, Katharina Mack, Kathrin Starz, Matthias Geyer, Frederic Bibollet-Ruche, Christina Stürzel, Marie Leoz, Jean Christophe Plantier, Ayele Argaw-Denboba, Emanuela Balestrieri, Annalucia Serafino, Ilaria Bucci, Chiara Cipriani, Corrado Spadafora, Paolo Sinibaldi-Vallebona, Claudia Matteucci, S. Nandi Jayashree, Ujjwal Neogi, Anil K. Chhangani, Shravan Sing Rathore, Bajrang R. J. Mathur, Adeyemi Abati, B. Taylan Koç, Tuba Çiğdem Oğuzoğlu, Takatoshi Shimauchi, Stephan Caucheteux, Jocelyn Turpin, Katja Finsterbusch, Yoshiki Tokura, Shanti Souriant, Luciana Balboa, Karine Pingris, Denise Kviatcowsky, Brigitte Raynaud-Messina, Céline Cougoule, Ingrid Mercier, Marcelo Kuroda, Pablo González-Montaner, Sandra Inwentarz, Eduardo Jose Moraña, Maria del Carmen Sasiain, Olivier Neyrolles, Isabelle Maridonneau-Parini, Geanncarlo Lugo-Villarino, Christel Vérollet, Alexandra Herrmann, Dominique Thomas, Nerea Ferreirós Bouzas, Xavier Lahaye, Anvita Bhargava, Takeshi Satoh, Matteo Gentili, Silvia Cerboni, Aymeric Silvin, Cécile Conrad, Hakim Ahmed-Belkacem, Elisa C. Rodriguez, Jean-François Guichou, Nathalie Bosquet, Matthieu Piel, Roger Le Grand, Megan King, Jean-Michel Pawlotsky, Nicolas Manel, Henning Hofmann, Benedicte Vanwalscappel, Nicolin Bloch, Nathaniel Landau, Stanislav Indik, Benedikt Hagen, José Carlos Valle-Casuso, Awatef Allouch, Annie David, Françoise Barré-Sinoussi, Monsef Benkirane, Gianfranco Pancino, Asier Saez-Cirion, Wing-Yiu Lee, Richard Sloan, Bianca Schulte, Silvana Opp, Jonas Blomberg, Luana Vargiu, Patricia Rodriguez-Tomé, Enzo Tramontano, Göran Sperber, Namita Kumari, Tatiana Ammosova, Sharmeen Diaz, Patricia Oneal, Sergei Nekhai, Audrey Fahrny, Gustavo Gers-Huber, Annette Audigé, Anitha Jayaprakash, Ravi Sachidanandam, Matt Hernandez, Marsha Dillon-White, Shanti Souriant, Karine Pingris, Brigitte Raynaud-Messina, Céline Cougoule, Ingrid Mercier, Olivier Neyrolles, Isabelle Maridonneau-Parini, Geanncarlo Lugo-Villarino, Emmanuel Maze, Claire Ham, Neil Almond, Greg Towers, Robert Belshaw, Patrícia de Sousa-Pereira, Joana Abrantes, Massimo Pizzato, Pedro J. Esteves, Tanja Kahle, Sven Schmitt, Laura Merkel, Nina Reuter, Thomas Stamminger, Ilaria Dalla Rosa, Kate Bishop, Antonella Spinazzola, Harriet Groom, Gabrielle Vieyres, Mathias Müsken, Thomas Zillinger, Veit Hornung, Winfried Barchet, Susanne Häussler, Thomas Pietschmann, Aneela Javed, Nicole Leuchte, Gabriela Salinas, Lennart Opitz, Sieghart Sopper, Christiane Mummert, Christian Hofmann, Angela G. Hückelhoven, Silke Bergmann, Sandra M. Müller-Schmucker, Ellen G. Harrer, Jan Dörrie, Niels Schaft, Thomas Harrer, Laure Cardinaux, M.- L. Zahno, H.- R. Vogt, R. Zanoni, G. Bertoni, Maximilian Muenchhoff, Philip Goulder, Oliver Keppler, Stephanie Rebensburg, Markus Helfer, Yuwei Zhang, Huicheng Chen, Annie Bernier, Annie Gosselin, Jean- Pierre Routy, Birgitta Wöhrl, Anna Schneider, Angela Corona, Imke Spöring, Mareike Jordan, Bernd Buchholz, Elias Maccioni, Roberto Di Santo, Kristian Schweimer, Christian Schölz, Brian Weinert, Sebastian Wagner, Petra Beli, Yasuyuki Miyake, Jun Qi, Lars Jensen, Werner Streicher, Anna McCarthy, Nicholas Westwood, Sonia Lain, Jürgen Cox, Patrick Matthias, Matthias Mann, James Bradner, Chunaram Choudhary, Marcel Stern, Elena Valletta, Caterina Frezza, Francesca Marino-Merlo, Sandro Grelli, Anna Lucia Serafino, Antonio Mastino, Beatrice Macchi, Meike Kaulfuß, Sonja Windmann, Wibke Bayer, Sello Mikasi, Graeme Jacobs, Rebecca Heß, Michael Storcksdieck gen. Bonsmann, Carsten Kirschning, Bernd Lepenies, Anne Kolenbrander, Vladimir Temchura, Kenta Iijima, Junya Kobayashi, Yukihito Ishizaka

**Affiliations:** 1 Molecular Virology and Gene Therapy, Molecular Medicine, Leuven, Belgium; 2 Molecular Virology, Technical University Dresden, Dresden, Germany; 3 Potsdam University, Potsdam, Germany; 4 Center for Regenerative Therapies Dresden, Dresden, Germany; 5 The Francis Crick Institute, London, Great Britain; 6 Dana Farber Cancer Institute, Boston, MA United States; 7 CIRI, Lyon, France; 8 National Cancer Institute, Frederick, MD United States; 9 Virology, Max von Pettenkofer-Institut, LMU Munich, Munich, Germany; 10 Institute of Medical Virology, University of Frankfurt, Frankfurt a. M., Germany; 11 German Center for Infection Research, Munich, Germany; 12 Institute of Medical Virology, University Hospital Frankfurt, Frankfurt a. M., Germany; 13 Integrative structural Biology, IGBMC, Illkirch, France; 14 Institut Pluridisciplinaire Hubert Curien, Strasbourg, France; 15 Institut Cochin, Paris, France; 16 CNRS, UMR5234 MFP Lab, Bordeaux, France; 17 Associated International Laboratory (LIA) Microbiology and Immunology, CNRS/Université de Bordeaux/Heinrich Pette Institute-Leibniz Institute for Experimental Virology, Bordeaux/Hamburg, France; 18 Institute of Microbiology, University Hospital Center and University of Lausanne, Lausanne, Switzerland; 19 Imagine Institute, Paris Descartes University, Paris, France; 20 J. Craig Venter Institute, La Jolla, CA United States; 21 Infection, University College London, London, Great Britain; 22 Medicine, Imperial College, London, Great Britain; 23 Microbiology & Infection, University of Montreal, Montreal, Great Britain; 24 CRCHUM, Montreal, Canada; 25 University College London, LMCB, London, Great Britain; 26 MRC Technology, Centre for Therapeutic Discovery, London, Great Britain; 27 Section of Virology, Department of Medicine, Imperial College London, London, Great Britain; 28 Molecular Virology, University of Brussels, Gosselies, Belgium; 29 Australian Institute for Bioengineering and Nanotechnology, Brisbane, Australia; 30 Université Libre de Bruxelles, Bruxelles, Belgium; 31 Laboratory of Experimental Hematology, University of Brussels, Brussels, Belgium; 32 Institut Universitaire de Technologie (IUT) Louis Pasteur de Schiltigheim, University of Strasbourg, Schiltigheim, France; 33 Medical Microbiology, Academic Medical Center, Amsterdam, Netherlands; 34 Medical Biochemistry, Academic Medical Center, Amsterdam, Netherlands; 35 Laboratory of Experimental Virology, Academic Medical Center of the University of Amsterdam, Amsterdam, Netherlands; 36 Division of Infectious Diseases, University of Cambridge, Cambridge, Great Britain; 37 Division of Virology, National Institute for Biological Standards and Control, Potters Bar, Great Britain; 38 Virology, NIBSC, South Mimms, Great Britain; 39 Institute for Virology and Immunbiology, Julius-Maximilians-Universität Würzburg, Würzburg, Germany; 40 Department of Medicine, University of Cambridge, Cambridge, Great Britain; 41 Institute of Medical Microbiology and Hygiene, University of Regensburg, Regensburg, Germany; 42 Institute of Clinical and Molecular Virology, Friedrich-Alexander-Universität Erlangen-Nuremberg, Erlangen, Germany; 43 Fraunhofer Institute for Integrated Circuits IIS, Erlangen, Germany; 44 Department of Biochemistry, Faculty of Medicine and Health Sciences, Ghent University, Campus Rommelaere, Ghent, Belgium; 45 Center for Retrovirus Research, The Ohio State University, Columbus, GA United States; 46 HIV Dynamics and Replication, National Cancer Institute, Frederick, MD United States; 47 Institute of Molecular Virology, Ulm University Medical Center, Ulm, Germany; 48 Dept. of Virology, Max von Pettenkofer Institute, LMU Munich, Munich, Germany; 49 Dept. of Microbial Pathogenesis, Yale University, School of Medicine, New Haven, CT United States; 50 Division of Biology and Biological Engineering, California Institute of Technology, Pasadena, CA United States; 51 Department of Medicine, School of Medicine, Yale University, New Haven, CT United States; 52 Center for Immunology and Inflammatory Diseases, Harvard Medical School, Boston, MA United States; 53 Program in Molecular Medicine, University of Massachusetts Medical School, Worcester, MA United States; 54 Department of Medicine, University of Pennsylvania, Philadelphia, PA United States; 55 Department of Microbiology, University of Pennsylvania, Philadelphia, PA United States; 56 Department of Pathology, University of Georgia, Athens, GA United States; 57 German Primate Centre, Göttingen, Germany; 58 Deutsches Primatenzentrum GmbH, Infektionsmodelle, Göttingen, Germany; 59 Emory Vaccine Center and Yerkes National Primate Research Center, Emory University, Atlanta, GA United States; 60 Center for Vaccine Research, University of Pittsburgh, Pittsburgh, PA United States; 61 Unité de Régulation des Infections Rétrovirales, Institut Pasteur, Paris, France; 62 Virology, Institut Pasteur, Paris, France; 63 Infection Models, German Primate Center, Göttingen, Germany; 64 Ulm University, Ulm, Germany; 65 Medical Biotechnology, Paul-Ehrlich-Institut, Langen, Germany; 66 Mater Research Institute, Brisbane, Australia; 67 GENYO, Granada, Germany; 68 Max-Delbrück-Center for Molecular Medicine, Berlin, Germany; 69 Hanover Medical School, Hanover, Germany; 70 University of Copenhagen, Copenhagen, Denmark; 71 Zoology, University of Oxford, Oxford, Great Britain; 72 Host-Pathogen Interactions, Paul-Ehrlich-Institute, Langen, Germany; 73 Laboratory of Protein Phosphorylation and Proteomics, Department of Cellular and Molecular Medicine, KU Leuven, Leuven, Belgium; 74 Division of Allergology, Paul-Ehrlich-Institute, Langen, Germany; 75 Department of Microbiology and Immunology, Albert Einstein College of Medicine, New York City, NY United States; 76 Immunity and Pathogenesis Program, Sanford Burnham Prebys Medical Discovery Institute, La Jolla, CA Germany; 77 Virology, Max von Pettenkofer Institute, LMU, Munich, Germany; 78 Institute for Medical Virology, Frankfurt a. M., Germany; 79 Department of Infectious Diseases, Integrative Virology, University Hospital Heidelberg, Heidelberg, Germany; 80 Department of Pediatrics, Center for Drug Discovery, Atlanta, GA United States; 81 Host-Pathogen-Interactions, Paul-Ehrlich-Institute, Langen, Germany; 82 Fred Hutchinson Cancer Research Center, Seattle, WA United States; 83 Department of General Pediatrics, Münster, Germany; 84 Department of Pediatrics, Emory University, Atlanta, GA United States; 85 Emory University School of Medicine, Atlanta, GA United States; 86 Institute of Virology, Universitätsklinikum Erlangen, Erlangen, Germany; 87 Institute of Clinical and Molecular Virology, Friedrich-Alexander University Erlangen-Nuremberg, Erlangen, Germany; 88 Institute for Immunology, Technical University Dresden, Dresden, Germany; 89 Institute of Virology, Helmholtz Center Munich, Neuherberg, Germany; 90 Ludwig-Maximilians-University Munich, Munich, Germany; 91 Institute of Medical Virology, University Hospital Tuebingen, Tuebingen, Germany; 92 Institute of Medical Virology and Epidemiology of Virus Diseases, University Medical Center Tübingen, Tübingen, Germany; 93 Experimental Virology, Twincore, Hanover, Germany; 94 School of Biomedical Sciences, The University of Hong Kong, Pokfulam, Hong Kong; 95 Department of Microbiology, The University of Hong Kong, Pokfulam, Hong Kong; 96 Institute for Virology, University Hospital Essen, Essen, Germany; 97 University of Pennsylvania, Philadelphia, PA United States; 98 Center for AIDS Research, Kumamoto University, Kumamoto, Japan; 99 Institute of Molecular Embryology and Genetics, Kumamoto University, Kumamoto, Japan; 100 EPFL, Lausanne, Switzerland; 101 Institut Pasteur, Paris, France; 102 Lady Davis Institute, McGill University AIDS Centre, Montreal, Canada; 103 Computational Evolution, ETH Zurich, D-BSSE, Basel, Switzerland; 104 Institute of Medical Virology, University of Zurich, Zurich, Switzerland; 105 Faculty of Health Sciences, University of the Witwatersrand, Johannesburg, South Africa; 106 Infectious Disease and Hospital Epidemiology, University Hospital Zurich, Zurich, Switzerland; 107 Department of Biological Chemistry, Weizmann Institute of Science, Rehovot, Israel; 108 Department of Microbiology and The Global Health and Emerging Pathogens Institute, Icahn School of Medicine at Mount Sinai, New York City, NY United States; 109 Division of Infectious Diseases, Department of Medicine, School of Medicine at Mount Sinai, New York City, NY United States; 110 Infectious Diseases, University hospital Zurich, Zurich, Switzerland; 111 KU Leuven, Leuven, Belgium; 112 Gladstone Institute of Virology and Immunology, University of California, San Francisco, CA United States; 113 Centre for Integrative Biology (CIBIO), Trento, Italy; 114 Institute for Clinical Microbiology and Hygiene, University Regensburg, Regensburg, Germany; 115 Centro Nacional de Biotecnología, Madrid, Spain; 116 Biodesign Institute, Arizona State University, Tempe, AZ United States; 117 Duke University Medical Center, Durham, NC United States; 118 Vaccine Research Center, National Institutes of Health, Bethesda, MD United States; 119 University of California, Irvine, CA United States; 120 Center for Virology and Vaccine Research, Beth Israel Deaconess Medical Center, Boston, MA United States; 121 Statistical Center for HIV/AIDS Research and Prevention, Fred Hutchinson Cancer Research Center, Seattle, WA United States; 122 Sanofi Pasteur, Swiftwater, PA United States; 123 Novartis Vaccines and Diagnostics, Inc., Cambridge, MA United States; 124 Advanced BioScience Laboratories, Inc., Rockville, MD United States; 125 EuroVacc Foundation, Lausanne, Switzerland; 126 Department of Veterinary Medicine, University of Cambridge, Cambridge, Great Britain; 127 Centre Hospitalier Universitaire Vaudois, University of Lausanne, Lausanne, Switzerland; 128 Department of Molecular and Medical Virology, Ruhr-University Bochum, Bochum, Germany; 129 Department of Immunology and Microbial Science, The Scripps Research Institute, La Jolla, CA United States; 130 Ragon Institute of MGH, MIT and Harvard, Cambridge, MA United States; 131 Research Center, Novartis Vaccines and Diagnostics S.r.l. (a GSK Company), Siena, Italy; 132 Center for Cancer Research, National Cancer Institute, Frederick, MD United States; 133 Ciencias Biologicas, Universidad Andres Bello, Viña del Mar, Chile; 134 Millenium Nucleus Biology of Neuropsiquiatric Disorders NuMIND, Valparaiso, Chile; 135 Department of Chemistry and Pharmacy, Friedrich-Alexander-University Erlangen-Nuremberg, Erlangen, Germany; 136 Clinical Virology and Infection Immunology, Institute of Microbiology and Hygiene, Regensburg, Germany; 137 Department of Dermatology, Cardiff University, Cardiff, Great Britain; 138 Department of Dermatology, Institute of Infection and Immunity, Cardiff University, Cardiff, Great Britain; 139 Virology, National AIDS Research Institute, Pune, India; 140 Department of Health and Biomedical Sciences, Symbiosis International University, Pune, India; 141 Academic Department, National Institute of Virology, Pune, India; 142 Research Institute for Medicines, University of Lisbon, Lisbon, Portugal; 143 INSERM, University Paris-Diderot, Paris, France; 144 Institut de Biologie Moleculaire et Cellulaire, Strasbourg, France; 145 Université François Rabelais, Tours, France; 146 Department 1, FG18, Robert Koch Institute, Berlin, Germany; 147 FG18 HIV and Other Retroviruses, Robert Koch Institute, Berlin, Germany; 148 Lady Davis Institute, Jewish General Hospital, McGill AIDS Centre, Montreal, Canada; 149 Advanced Biomedical Computing Center, Leidos Biomedical Research, Inc, Frederick, MD United States; 150 HIV Dynamics and Replication Program, National Cancer Institute, Frederick, MD United States; 151 Frderick National Lab for Cancer Research, Leidos Biomedical Research, Inc, Frederick, MD United States; 152 Division of Infectious Disease, University of Pittsburgh, Pittsburgh, PA United States; 153 Tuffs University, Boston, MA United States; 154 Department of Microbiology & Immunology, Albert Einstein College of Medicine, New York City, NY United States; 155 Laboratory of Structural Biology, International Institute of Molecular and Cell Biology, Warszawa, Poland; 156 Chemical Department, Lomonosov Moscow State University, Moscow, Russian Federation; 157 Faculty of bioengineering and Bioinformatics, Lomonosov Moscow State University, Moscow, Russian Federation; 158 Belozersky Institute of Physical and Chemical Biology, Lomonosov Moscow State University, Moscow, Russian Federation; 159 Shemyakin-Ovchinnikov Institute of Bioorganic Chemistry of the Russian Academy of Sciences, Moscow, Russian Federation; 160 National Research University Higher School of Economics, Moscow, Russian Federation; 161 Biological Sciences, Texas Tech University, Lubbock, TX United States; 162 ICGEB, Trieste, Italy; 163 Laboratory of Experimental Virology, CINIMA AMC, University of Amsterdam, Amsterdam, Netherlands; 164 School of Medicine, College of Health and Agricultural Sciences, UCD-Centre for Research in Infectious Diseases, University College Dublin, Dublin, Ireland; 165 Clinical Chemistry, Microbiology, and Immunology, Ghent University, Ghent, Belgium; 166 Internal Medicine, Ghent University and Ghent University Hospital, Ghent, Belgium; 167 VIB Medical Biotechnology Center, Ghent, Belgium; 168 Laboratory of Viral and Cellular Genetics, Institute of Molecular Genetics of the ASCR, Prague, Czech Republic; 169 Dpt. of Viral and Cellular Genetics, Institute of Molecular Genetics, Prague 4, Czech Republic; 170 Dept. Viral Infections, RIMD, Osaka University, Osaka, Japan; 171 Pathogen Genomics Center, National Institute of Infectious Diseases, Tokyo, Japan; 172 Institute for Virology and Immunobiology, University Würzburg, Würzburg, Germany; 173 Institute for Virology and Immunobiology, Heinrich-Heine-University Düsseldorf, Würzburg, Germany; 174 Division of Bioinformatics, Institute of Biochemistry, Erlangen, Germany; 175 DKFZ, F020, Heidelberg, Germany; 176 University of Heidelberg, Heidelberg, Germany; 177 NIH, NIAID, Bethesda, Great Britain; 178 Faculty of Pharmacy, University of Lisbon, Lisbon, Portugal; 179 Institute of Virology, Friedrich-Alexander University Erlangen-Nuremberg, Erlangen, Germany; 180 Institute of Biochemistry, Charité Medical University Berlin, Berlin, Germany; 181 Department of Otorhinolaryngology, Head and Neck Surgery, Friedrich-Alexander University Erlangen-Nuremberg, Erlangen, Germany; 182 Department of Chemistry and Center for Pharmacy, University of Bergen, Bergen, Norway; 183 Technion, Biology, Haifa, Israel; 184 Virology, Bernhard Nocht Institute for Tropical Medicine, Hamburg, Germany; 185 Microbiology, University of Ulsan College of Med., Seoul, South Korea; 186 Department of Pathology, Third Faculty of Medicine, Charles University in Prague, Prague, Czech Republic; 187 Medical Research Council Clinical Sciences Centre, Imperial College London, London, Great Britain; 188 Laboratory of Viral and Cellular Genetics, Institute of Molecular Genetics, ASCR, Prague, Czech Republic; 189 Department of Animal and Avian Sciences, University of Maryland, College Park, MA United States; 190 Institute of Molecular Genetics, Prague, Czech Republic; 191 MRC-University of Glasgow Centre for Virus Research, Glasgow, Great Britain; 192 Scuola Normale Superiore, Pisa, Italy; 193 Aaron Diamond AIDS Research Center, New York City, NY United States; 194 Università of Pisa, Pisa, Italy; 195 Lebanese University, Al Fanar, Lebanon; 196 MRC-University of Glasgow Centre for Virology, Glasgow, Great Britain; 197 London School of Hygiene and Tropical Medicine, London, Great Britain; 198 Department of Pathology, Stellenbosch University, Cape Town, South Africa; 199 Department of Physical Biochemistry, Max Planck Institute of Molecular Physiology, Dortmund, Germany; 200 Laboratoire de Virologie, CHU Charles Nicolle, Rouen, France; 201 EA 2656 GRAM, Université de Rouen, Rouen, France; 202 Laboratoire associé au Centre National de Référence du VIH, CHU Charles Nicolle, Rouen, France; 203 Department of Experimental Medicine and Surgery, University of Rome Tor Vergata, Rome, Italy; 204 Institute of Translational Pharmacology, National Research Council, Rome, Italy; 205 Dept. of Microbiology and Immunology, Albert Einstein College of Medicine, New York City, NY United States; 206 HIV Dynamics and Replication Program, Retrovirus Assembly Laboratory, National Cancer Institute, Frederick, MD United States; 207 Division of Clinical Microbiology, F68, Department of Laboratory Medicine, Karolinska Institute, Stockholm, Sweden; 208 Department of Environmental Science, Maharaja Ganga Singh University, Rajasthan, India; 209 Machiya Biological Park, Veterinary Center, Jodhpur Rajasthan, India; 210 Kamla Nehru Nagar, 1B1, Jodhpur Rajasthan, India; 211 Luth, Publi Health, Lagos, Nigeria; 212 Faculty of Veterinary Medicine, Virology, Adnan Menderes University, Aydin, Turkey; 213 Faculty of Veterinary Medicine, Virology, Ankara University, Ankara, Turkey; 214 Department of Dermatology, Hamamatsu University School of Medicine, Hamamatsu, Japan; 215 IPBS, CNRS UMR 5089, Toulouse, France; 216 Inmunologia de Enfermedades Respiratorias, Instituto de Medicina Experimental (IMEX)-CONICET, Buenos Aires, Argentina; 217 Division of Immunology, Tulane National Primate Research Center, Covington, LA United States; 218 Department of Microbiology and Immunology, School of Medicine, Tulane University, New Orleans, LA United States; 219 Instituto Prof. Dr. Raúl Vaccarezza, Hospital de Infecciosas Dr. F.J. Muñiz, Buenos Aires, Argentina; 220 Institute of Clinical Pharmacology, Frankfurt a. M., Germany; 221 Institut Curie, Inserm, U932, Paris, France; 222 Department of Virology, Hôpital Henri Mondor, Créteil, France; 223 School of Medicine, Yale University, New Haven, CT United States; 224 CNRS, UMR508, Montpellier, France; 225 CEA, IDMIT Center, Fontenay-aux-Roses, France; 226 Institut Curie, CNRS, UMR144, Paris, France; 227 HIV and Other Retroviruses, Robert Koch Institut, Berlin, Germany; 228 Microbiology, New York University, New York City, NY United States; 229 Institute of Virology, University of Veterinary Med, Vienna, Austria; 230 Institute of Human Genetics, CNRS UPR 1142, Montpellier, France; 231 Barts and the London School of Medicine, Blizard Institute, London, Great Britain; 232 Microbiology and Immunology, Albert Einstein College of Medicine, New York City, NY United States; 233 Medical Sciences, Uppsala University, Uppsala, Sweden; 234 Life and Environmental Sciences, Cagliari University, Cagliari, Sweden; 235 Nurideas SRL, Cagliari, Sweden; 236 Neuroscience, Uppsala University, Uppsala, Sweden; 237 Medicine, Howard University, Washington, DC United States; 238 Department of Oncological Sciences, Icahn School of Medicine at Mount Sinai, New York City, NY United States; 239 Peninsula Schools of Medicine and Dentistry, Plymouth University, Plymouth, Great Britain; 240 Infection and Immunity, University College London, London, Great Britain; 241 CIBIO/InBIO, University of Porto, Vairão, Portugal; 242 Faculty of Sciences, University of Porto, Porto, Portugal; 243 Centre for Integrative Biology, University of Trento, Trento, Italy; 244 CITS, CESPU, Gandra, Portugal; 245 Institute of Clinical Chemistry and Pharmacology, University Hospital Bonn, Bonn, Germany; 246 Francis Crick Institute, Mill Hill, London, Great Britain; 247 Molecular Bacteriology, TWINCORE, Hanover, Germany; 248 Clinical Chemistry and Clinical Pharmacology, University of Bonn, Bonn, Germany; 249 Gene Center and Department of Biochemistry, Ludwig-Maximilians-University Munich, Munich, Germany; 250 Atta-ur-Rahman School of Applied Biosciences (ASAB), National University of Sciences and Technology, Islamabad, Pakistan; 251 Transcriptome and Genome Analysis Laboratory (TAL), Faculty of Medicine, University Göttingen, Göttingen, Germany; 252 Functional Genomics Center Zurich, Swiss Federal Institute of Technology, Zurich, Switzerland; 253 Tumor Immunology Lab, Cancer Research Institute, Medical University Innsbruck and Tyrolean, Innsbruck, Austria; 254 Infectious Diseases Section, Department of Internal Medicine III, Universitätsklinikum Erlangen, Erlangen, Germany; 255 Department of Dermatology, Universitätsklinikum Erlangen, Erlangen, Germany; 256 Division of Infectious Diseases, University of California, Los Angeles, LA United States; 257 Department of Internal Medicine 5, University Hospital Heidelberg, Heidelberg, Germany; 258 Institute of Virology and Immunology, Vetsuisse Faculty, University of Bern, Bern, Switzerland; 259 Virology, Max von Pettenkofer-Institute, Munich, Germany; 260 Paediatrics, University of Oxford, Oxford, Great Britain; 261 College of Pharmacy/Ohio State University, Columbus, OH United States; 262 McGill University Health Centre, Montreal, Canada; 263 Biopolymere, University Bayreuth, Bayreuth, Germany; 264 Life and Environmental Sciences, University of Cagliari, Monserrato Cagliari, Italy; 265 Medical Institution Mannheim, University Heidelberg, Heidelberg, Germany; 266 University of Rome, Rome, Italy; 267 Virology, Max-von-Pettenkofer Institute, LMU, Munich, Germany; 268 NNF CPR, University Copenhagen, Copenhagen, Denmark; 269 Friedrich Miescher Institute, Basel, Switzerland; 270 Dana-Farber Cancer Institute, Harvard Medical School, Boston, MA United States; 271 Karolinska Institutet, Stockholm, Sweden; 272 EaStCHEM, St. Andrews, Great Britain; 273 Max Planck Institute for Biochemistry, Martinsried, Germany; 274 Systems Medicine, University of Rome Tor Vergata, Rome, Italy; 275 Chemical, Biological, Pharmaceutical and Environmental Sciences, University of Messina, Messina, Italy; 276 Experimental Medicine and Surgery, University of Rome Tor Vergata, Rome, Italy; 277 CNR, The Institute of Translational Pharmacology, Rome, Italy; 278 Molecular and Medical Virology, Ruhr-University Bochum, Bochum, Germany; 279 Medical Microbiology, University Hospital Essen, Essen, Germany; 280 Infection Immunology, University of Veterinary Medicine Foundation, Hanover, Germany; 281 Department of Intractable Diseases, Research Institute, National Center for Global Health and Medicine, Tokyo, Japan; 282 Department of Genome Repair Dynamics, Radiation Biology Center, Kyoto University, Kyoto, Japan

## Abstract

Oral presentations

Session 1: Entry & uncoating

O1 Host cell polo-like kinases (PLKs) promote early prototype foamy virus (PFV) replication

Irena Zurnic, Sylvia Hütter, Ute Lehmann, Nicole Stanke, Juliane Reh, Tobias Kern, Fabian Lindel, Gesche Gerresheim, Martin Hamann, Erik Müllers, Paul Lesbats, Peter Cherepanov, Erik Serrao, Alan Engelman, Dirk Lindemann

O2 A novel entry/uncoating assay reveals the presence of at least two species of viral capsids during synchronized HIV-1 infection

Claire Da Silva Santos, Kevin Tartour, Andrea Cimarelli

O3 Dynamics of nuclear envelope association and nuclear import of HIV-1 complexes

Rya Burdick, Jianbo Chen, Jaya Sastri, Wei-Shau Hu, Vinay Pathak

O4 Human papillomavirus protein E4 potently enhances the susceptibility to HIV infection

Oliver T. Keppler

Session 2: Reverse transcription & integration

O5 Structure and function of HIV-1 integrase post translational modifications

Karine Pradeau, Sylvia Eiler, Nicolas Levy, Sarah Lennon, Sarah Cianferani, Stéphane Emiliani, Marc Ruff

O6 Regulation of retroviral integration by RNA polymerase II associated factors and chromatin structure

Vincent Parissi

Session 3: Transcription and latency

O7 A novel single-cell analysis pipeline to identify specific biomarkers of HIV permissiveness

Sylvie Rato, Antonio Rausell, Miguel Munoz, Amalio Telenti, Angela Ciuffi

O8 A capsid-dependent integration program linking T cell activation to HIV-1 gene expression

Alexander Zhyvoloup, Anat Melamed, Ian Anderson, Delphine Planas, Janos Kriston-Vizi, Robin Ketteler, Chen-Hsuin Lee, Andy Merritt, Petronela Ancuta, Charles Bangham, Ariberto Fassati

O9 Characterisation of new RNA polymerase III and RNA polymerase II transcriptional promoters in the Bovine Leukemia Virus genome

Anthony Rodari, Benoit Van Driessche, Mathilde Galais, Nadége Delacourt, Sylvain Fauquenoy, Caroline Vanhulle, Anna Kula, Arsène Burny, Olivier Rohr, Carine Van Lint

O10 Tissue-specific dendritic cells differentially modulate latent HIV-1 reservoirs

Thijs van Montfort, Renee van der Sluis, Dave Speijer, Ben Berkhout

Session 4: RNA trafficking & packaging

O11 A novel *cis*-acting element affecting HIV replication

Bo Meng, Andrzej Rutkowski, Neil Berry, Lars Dölken, Andrew Lever

O12 Tolerance of HIV’s late gene expression towards stepwise codon adaptation

Thomas Schuster, Benedikt Asbach, Ralf Wagner

Session 5: Assembly & release

O13 Importance of the tax-inducible actin-bundling protein fascin for transmission of human T cell leukemia virus Type 1 (HTLV-1)

Christine Gross, Veit Wiesmann, Martina Kalmer, Thomas Wittenberg, Jan Gettemans, Andrea K. Thoma-Kress

O14 Lentiviral nef proteins antagonize TIM-mediated inhibition of viral release

Minghua Li, Eric O. Freed, Shan-Lu Liu

Session 6: Pathogenesis & evolution

O15 SEVI and semen prolong the half-life of HIV-1

Janis Müller, Jan Münch

O16 CD169^+^ macrophages mediate retrovirus *trans*-infection of permissive lymphocytes to establish infection in vivo

Xaver Sewald, Pradeep Uchil, Mark Ladinsky, Jagadish Beloor, Ruoxi Pi, Christin Herrmann, Nasim Motamedi, Thomas Murooka, Michael Brehm, Dale Greiner, Thorsten Mempel, Pamela Bjorkman, Priti Kumar, Walther Mothes

O17 Efficient replication of a *vpu* containing SIVagm construct in African Green Monkeys requires an HIV-1 *nef* gene

Simone Joas, Erica Parrish, Clement Wesley Gnanadurai, Edina Lump, Christina M. Stürzel, Nicholas F. Parrish, Ulrike Sauermann, Katharina Töpfer, Tina Schultheiss, Steven Bosinger, Guido Silvestri, Cristian Apetrei, Nicholas Huot, Michaela Müller-Trutwin, Daniel Sauter, Beatrice H. Hahn, Christiane Stahl-Hennig, Frank Kirchhoff

O18 Reprogramming initiates mobilization of endogenous mutagenic LINE-1, *Alu* and SVA retrotransposons in human induced pluripotent stem cells with consequences for host gene expression

Gerald Schumann, Sabine Jung-Klawitter, Nina V. Fuchs, Kyle R. Upton, Martin Muñoz-Lopez, Ruchi Shukla, Jichang Wang, Marta Garcia-Canadas, Cesar Lopez-Ruiz, Daniel J. Gerhardt, Attila Sebe, Ivana Grabundzija, Patricia Gerdes, Sylvia Merkert, Andres Pulgarin, Anja Bock, Ulrike Held, Anett Witthuhn, Alexandra Haase, Ernst J. Wolvetang, Ulrich Martin, Zoltán Ivics, Zsuzsanna Izsvák, J. Garcia-Perez, Geoffrey J. Faulkner

O19 NF-κB activation induces expression of human endogenous retrovirus and particle production

Tara Hurst, Aris Katzourakis, Gkikas Magiorkinis

Session 7a and b: Innate sensing & intrinsic immunity

O20 Identification of the phosphatase acting on T592 in SAMHD1 during M/G_1_ transition

Kerstin Schott, Rita Derua, Janna Seifried, Andreas Reuter, Heike Schmitz, Christiane Tondera, Alberto Brandariz-Nuñez, Felipe Diaz-Griffero, Veerle Janssens, Renate König

O21 Vpx overcomes a SAMHD1-independent block to HIV reverse transcription that is specific to resting CD4 T cells

Hanna-Mari Baldauf, Lena Stegmann, Sarah-Marie Schwarz, Maud Trotard, Margarethe Martin, Gina Lenzi, Manja Burggraf, Xiaoyu Pan, Oliver I. Fregoso, Efrem S. Lim, Libin Abraham, Elina Erikson, Laura Nguyen, Ina Ambiel, Frank Rutsch, Renate König, Baek Kim, Michael Emerman, Oliver T. Fackler, Oliver T. Keppler

O22 The role of SAMHD1 in antiviral restriction and immune sensing in the mouse

Sabine Wittmann, Rayk Behrendt, Bianca Volkmann, Kristin Eissmann, Thomas Gramberg

O23 T cells expressing reduced restriction factors are preferentially infected in therapy naïve HIV-1 patients

Sebastian Bolduan, Herwig Koppensteiner, Stefanie Regensburg, Ruth Brack-Werner, Rika Draenert, Michael Schindler

O24 cGAS-mediated innate immunity spreads through HIV-1 env-induced membrane fusion sites from infected to uninfected primary HIV-1 target cells

Aurélie Ducroux, Shuting Xu, Aparna Ponnurangam, Sergej Franz, Angelina Malassa, Ellen Ewald, Christine Goffinet

O25 Perturbation of innate RNA and DNA sensing by human T cell leukemia virus type 1 oncoproteins

Sin-Yee Fung, Ching-Ping Chan, Chun-Kit Yuen, Kin-Hang Kok, Chin-Ping Chan, Dong-Yan Jin

O26 Induction and anti-viral activity of Interferon α subtypes in HIV-1 infection

Ulf Dittmer

O27 Vpu-mediated counteraction of tetherin is a major determinant of HIV-1 interferon resistance

Dorota Kmiec, Shilpa Iyer, Christina Stürzel, Daniel Sauter, Beatrice Hahn, Frank Kirchhoff

O28 DNA repair protein Rad18 restricts HIV-1 and LINE-1 life cycle

Yasuo Ariumi, Mariko Yasuda-Inoue, Koudai Kawano, Satoshi Tateishi, Priscilla Turelli

O29 Natural mutations in *IFITM3* allow escape from post-translational regulation and toggle antiviral specificity

Alex Compton, Nicolas Roy, Françoise Porrot, Anne Billet, Nicoletta Casartelli, Jacob Yount, Chen Liang, Oliver Schwartz

Session 8: Adaptive immunity & immune evasion

O30 Observing evolution in HIV-1 infection: phylogenetics and mutant selection windows to infer the influence of the autologous antibody response on the viral quasispecies

Carsten Magnus, Lucia Reh, Penny Moore, Therese Uhr, Jacqueline Weber, Lynn Morris, Alexandra Trkola

O31 Dose and subtype specific analyses of the anti-HIV *effects of IFN-alpha family members*

Rashel V. Grindberg, Erika Schlaepfer, Gideon Schreiber, Viviana Simon, Roberto F. Speck

Session 9: Novel antiviral strategies

O32 LEDGIN-mediated inhibition of the integrase-LEDGF/p75 interaction reduces reactivation of residual latent HIV

Zeger Debyser, Lenard Vranckx, Jonas Demeulemeester, Suha Saleh, Eric Verdin, Anna Cereseto, Frauke Christ, Rik Gijsbers

O33 NKG2D-mediated clearance of reactivated viral reservoirs by natural killer cells

O34 Inhibition of HIV reactivation in brain cells by AAV-mediated delivery of CRISPR/Cas9

O35 CRISPR-Cas9 as antiviral: potent HIV-1 inhibition, but rapid virus escape and the subsequent design of escape-proof antiviral strategies

Ben Berkhout, Gang Wang, Na Zhao, Atze T. Das

Session 10: Recent advances in HIV vaccine development

O36 Priming with a potent HIV-1 DNA vaccine frames the quality of T cell and antibody responses prior to a poxvirus and protein boost

Benedikt Asbach, Josef Köstler, Beatriz Perdiguero, Mariano Esteban, Bertram L. Jacobs, David C. Montefiori, Celia C. LaBranche, Nicole L. Yates, Georgia D. Tomaras, Guido Ferrari, Kathryn E. Foulds, Mario Roederer, Gary Landucci, Donald N. Forthal, Michael S. Seaman, Natalie Hawkins, Steven G. Self, Sanjay Phogat, James Tartaglia, Susan W. Barnett, Brian Burke, Anthony D. Cristillo, Song Ding, Jonathan L. Heeney, Giuseppe Pantaleo, Ralf Wagner

O37 Passive immunisation with a neutralising antibody against HIV-1 Env prevents infection of the first cells in a mucosal challenge rhesus monkey model

Christiane Stahl-Hennig, Viktoria Stab, Armin Ensser, Ulrike Sauermann, Bettina Tippler, Dennis Burton, Matthias Tenbusch, Klaus Überla

O38 HIV antibody Fc-glycoforms drive B cell affinity maturation

Galit Alter, Giuseppe Lofano, Anne-Sophie Dugast, Viraj Kulkarni, Todd Suscovich

Poster presentations

Topic 1: Entry & uncoating

P1 Dynein light chain is required for murine leukemia virus infection

Tatiana Opazo, Felipe Barraza, Diego Herrera, Andrea Garces, Tomas Schwenke, Diego Tapia, Jorge Cancino, Gloria Arriagada

P2 Peptide paratope mimics of the broadly neutralising HIV-1 antibody b12

Christina Haußner, Dominik Damm, Anette Rohrhofer, Barbara Schmidt, Jutta Eichler

P3 Investigating cellular pathways involved in the transmission of HIV-1 between dendritic cells and T cells using RNAi screening techniques

Rebecca Midgley, James Wheeldon, Vincent Piguet

P4 Co-receptor tropism in HIV-1, HIV-2 monotypic and dual infections

Priyanka Khopkar, Megha Rohamare, Smita Kulkarni

P5 Characterisation of the role of CIB1 and CIB2 as HIV-1 helper factors

Ana Godinho-Santos, Allan Hance, Joao Goncalves, Fabrizio Mammano

P6 Buffering deleterious polymorphisms in the highly constrained C2 region of HIV-1 envelope by the flexible V3 domain

Romain Gasser, Meriem Hamoudi, Martina Pellicciotta, Zhicheng Zhou, Clara Visdeloup, Philippe Colin, Martine Braibant, Bernard Lagane, Matteo Negroni

P7 Entry inhibition of HERV-K(HML-2) by an Env-IgG fusion protein

Jula Wamara, Norbert Bannert

Topic 2: Reverse transcription & integration

P8 The R263K/H51Y resistance substitutions in HIV integrase decreases levels of integrated HIV DNA over time

Thibault Mesplede, Nathan Osman, Kaitlin Anstett, Jiaming Calvin Liang, Hanh Thi Pham, Mark Wainberg

P9 The Retrovirus Integration Database (RID)

Wei Shao, Jigui Shan, Mary Kearney, Xiaolin Wu, Frank Maldarelli, John Mellors, Brian Luke, John Coffin, Stephen Hughes

P10 The small molecule 3G11 inhibits HIV-1 reverse transcription

Thomas Fricke, Silvana Opp, Caitlin Shepard, Dmitri Ivanov, Baek Kim, Jose Valle-Casuso, Felipe Diaz-Griffero

P11 Dual and opposite regulation of HIV-1 integration by hRAD51: impact on therapeutical approaches using homologous DNA repair modulators

Vincent Parissi

P12 A flexible motif essential for integration by HIV-1 integrase

Marine Kanja, Pierre Cappy, Matteo Negroni, Daniela Lener

P13 Interaction between HIV-1 integrase and the host protein Ku70: identification of the binding site and study of the influence on integrase-proteasome interplay

Ekaterina Knyazhanskaya, Andrey Anisenko, Timofey Zatsepin, Marina Gottikh

P14 Normalisation based method for deep sequencing of somatic retroelement integrations in human genome

Alexander Komkov, Anastasia Minervina, Gaiaz Nugmanov, Vadim Nazarov, Konstantin Khodosevich, Ilgar Mamedov, Yuri Lebedev

Topic 3: Transcription and latency

P15 BCA2/RABRING7 restricts HIV-1 transcription by preventing the nuclear translocation of NF-κB

Marta Colomer-Lluch, Ruth Serra-Moreno

P16 MATR3 post-transcriptional regulation of HIV-1 transcription during latency

Ambra Sarracino, Anna Kula, Lavina Gharu, Alexander Pasternak, Carine Van Lint, Alessandro Marcello

P17 HIV-1 tat intersects the SUMO pathway to regulate HIV-1 promoter activity

Ann Marie McCartin, Anurag Kulkarni, Valentin Le Douce, Virginie Gautier

P18 Conservation in HIV-1 Vpr guides tertiary gRNA folding and alternative splicing

Ann Baeyens, Evelien Naessens, Anouk Van Nuffel, Karin Weening, Anne-Marie Reilly, Eva Claeys, Wim Trypsteen, Linos Vandekerckhove, Sven Eyckerman, Kris Gevaert, Bruno Verhasselt

P19 The majority of reactivatable latent HIV are genetically distinct

Hoi Ping Mok, Nicholas Norton, Axel Fun, Jack Hirst, Mark Wills, Andrew Lever

P20 Do mutations in the *tat* exonic splice enhancer contribute to HIV-1 latency?

Nicholas Norton, Hoi Ping Mok, Jack Hirst, Andrew Lever

P21 Culture-to-Ct: A fast and direct RT-qPCR HIV gene reactivation screening method using primary T cell culture

Valentin Le Douce, Ann Marie McCartin, Virginie Gautier

P22 A novel approach to define populations of early silenced proviruses

Dalibor Miklik, Filip Senigl, Jiri Hejnar

Topic 4: RNA trafficking & packaging

P23 Functional analysis of the structure and conformation of HIV-1 genome RNA DIS

Jun-ichi Sakuragi, Sayuri Sakuragi, Masaru Yokoyama, Tatsuo Shioda, Hironori Sato

P24 Regulation of foamy viral *env* splicing controls *gag* and *pol* expression

Jochen Bodem, Rebecca Moschall, Sarah Denk, Steffen Erkelenz, Christian Schenk, Heiner Schaal

Topic 5: Assembly & release

P25 Transfer of HTLV-1 p8 to target T cells depends on VASP: a novel interaction partner of p8

Norbert Donhauser, Ellen Socher, Sebastian Millen, Heinrich Sticht, Andrea K. Thoma-Kress

P26 COL4A1 and COL4A2 are novel HTLV-1 tax targets with a putative role in virus transmission

Christine Gross, Sebastian Millen, Melanie Mann, Klaus Überla, Andrea K. Thoma-Kress

P27 The C terminus of foamy virus gag protein is required for particle formation, and virus budding: starting assembly at the C terminus?

Guochao Wei, Matthew J. Betts, Yang Liu, Timo Kehl, Robert B. Russell, Martin Löchelt

P28 Generation of an antigen-capture ELISA and analysis of Rec and Staufen-1 effects on HERV-K(HML-2) virus particle production

Oliver Hohn, Saeed Mostafa, Kirsten Hanke, Stephen Norley, Norbert Bannert

P29 Antagonism of BST-2/tetherin is a conserved function of primary HIV-2 Env glycoproteins

Chia-Yen Chen, Masashi Shingai, Pedro Borrego, Nuno Taveira, Klaus Strebel

P30 Mutations in the packaging signal region of the HIV-1 genome cause a late domain mutant phenotype

Chris Hellmund, Bo Meng, Andrew Lever

P31 p6 regulates membrane association of HIV-1 gag

Melanie Friedrich, Friedrich Hahn, Christian Setz, Pia Rauch, Kirsten Fraedrich, Alina Matthaei, Petra Henklein, Maximilian Traxdorf, Torgils Fossen, Ulrich Schubert

Topic 6: Pathogenesis & evolution

P32 Molecular and structural basis of protein evolution during viral adaptation

Aya Khwaja, Meytal Galilee, Akram Alian

P33 HIV-1 enhancement and neutralisation by soluble gp120 and its role for the selection of the R5-tropic “best fit”

Birco Schwalbe, Heiko Hauser, Michael Schreiber

P34 An insertion of seven amino acids in the Env cytoplasmic tail of Human Immunodeficiency Virus type 2 (HIV-2) selected during disease progression enhances viral replication

François Dufrasne, Mara Lucchetti, Patrick Goubau, Jean Ruelle

P35 Cell-associated HIV-1 unspliced to multiply spliced RNA ratio at 12 weeks ART correlates with markers of immune activation and apoptosis and predicts the CD4 T-cell count at 96 weeks ART

Mirte Scherpenisse, Ben Berkhout, Alexander Pasternak

P36 Faster progression in non-B subtype HIV-1-infected patients than Korean subclade of subtype B is accompanied by higher variation and no induction of gross deletion in non-B *nef* gene by Korean red ginseng treatment

Young-Keol Cho, Jungeun Kim, Daeun Jeong

P37 Aberrant expression of ERVWE1 endogenous retrovirus and overexpression of TET dioxygenases are characteristic features of seminoma

Katerina Trejbalova, Martina Benesova, Dana Kucerova, Zdenka Vernerova, Rachel Amouroux, Petra Hajkova, Jiri Hejnar

P38 Life history of the oldest lentivirus: characterisation of ELVgv integrations and the *TRIM5* selection pattern in dermoptera

Daniel Elleder, Tomas Hron, Helena Farkasova, Abinash Padhi, Jan Paces

P39 Characterisation of a highly divergent endogenous retrovirus in the equine germ line

Henan Zhu, Robert Gifford, Pablo Murcia

P40 The emergence of pandemic retroviral infection in small ruminants

Maria Luisa Carrozza, Anna-Maria Niewiadomska, Maurizio Mazzei, Mounir Abi-Said, Joseph Hughes, Stéphane Hué, Robert Gifford

P41 Near full-length genome (NFLG) Characterisation of HIV-1 subtype B identified in South Africa

Adetayo Obasa, Graeme Jacobs, Susan Engelbrecht

P42 Acquisition of Vpu-mediated tetherin antagonism by an HIV-1 group O strain

Katharina Mack, Kathrin Starz, Daniel Sauter, Matthias Geyer, Frederic Bibollet-Ruche, Christina Stürzel, Marie Leoz, Jean Christophe Plantier, Beatrice H. Hahn, Frank Kirchhoff

P43 The human endogenous retrovirus type K is involved in cancer stem cell markers expression and in human melanoma malignancy

Ayele Argaw-Denboba, Emanuela Balestrieri, Annalucia Serafino, Ilaria Bucci, Chiara Cipriani, Corrado Spadafora, Paolo Sinibaldi-Vallebona, Claudia Matteucci

P44 Natural infection of Indian non-human primates by unique lentiviruses

S. Nandi Jayashree, Ujjwal Neogi, Anil K. Chhangani, Shravan Sing Rathore, Bajrang R. J. Mathur

P45 Free cervical cancer screening among HIV-positive women receiving antiretroviral treatment in Nigeria

Adeyemi Abati

P46 Molecular evolutionary status of feline immunodeficiency virus in Turkey

B. Taylan Koç, Tuba Çiğdem Oğuzoğlu

Topic 7: Innate sensing & intrinsic immunity

P47 Cell-to-cell contact with HTLV-1-infected T cells reduces dendritic cell immune functions and contributes to infection *in trans*.

Takatoshi Shimauchi, Stephan Caucheteux, Jocelyn Turpin, Katja Finsterbusch, Charles Bangham, Yoshiki Tokura, Vincent Piguet

P48 Deciphering the mechanisms of HIV-1 exacerbation induced by *Mycobacterium tuberculosis* in monocytes/macrophages

Shanti Souriant, Luciana Balboa, Karine Pingris, Denise Kviatcowsky, Brigitte Raynaud-Messina, Céline Cougoule, Ingrid Mercier, Marcelo Kuroda, Pablo González-Montaner, Sandra Inwentarz, Eduardo Jose Moraña, Maria del Carmen Sasiain, Olivier Neyrolles, Isabelle Maridonneau-Parini, Geanncarlo Lugo-Villarino, Christel Vérollet

P49 The SAMHD1-mediated inhibition of LINE-1 retroelements is regulated by phosphorylation

Alexandra Herrmann, Sabine Wittmann, Caitlin Shepard, Dominique Thomas, Nerea Ferreirós Bouzas, Baek Kim, Thomas Gramberg

P50 Activities of nuclear envelope protein SUN2 in HIV infection

Xavier Lahaye, Anvita Bhargava, Takeshi Satoh, Matteo Gentili, Silvia Cerboni, Aymeric Silvin, Cécile Conrad, Hakim Ahmed-Belkacem, Elisa C. Rodriguez, Jean-François Guichou, Nathalie Bosquet, Matthieu Piel, Roger Le Grand, Megan King, Jean-Michel Pawlotsky, Nicolas Manel

P51 Activation of TLR7/8 with a small molecule agonist induces a novel restriction to HIV-1 infection of monocytes

Henning Hofmann, Benedicte Vanwalscappel, Nicolin Bloch, Nathaniel Landau

P52 Steady state between the DNA polymerase and Rnase H domain activities of reverse transcriptases determines the sensitivity of retroviruses to inhibition by APOBEC3 proteins

Stanislav Indik, Benedikt Hagen

P53 HIV restriction in mature dendritic cells is related to p21 induction and p21-mediated control of the dNTP pool and SAMHD1 activity.

José Carlos Valle-Casuso, Awatef Allouch, Annie David, Françoise Barré-Sinoussi, Michaela Müller-Trutwin, Monsef Benkirane, Gianfranco Pancino, Asier Saez-Cirion

P54 IFITM protens restrict HIV-1 protein synthesis

Wing-Yiu Lee, Chen Liang, Richard Sloan

P55 Characterisation and functional analysis of the novel restriction factor Serinc5

Bianca Schulte, Silvana Opp, Felipe Diaz-Griffero

P56 piRNA sequences are common in Human Endogenous Retroviral Sequences (HERVs): An antiretroviral restriction mechanism?

Jonas Blomberg, Luana Vargiu, Patricia Rodriguez-Tomé, Enzo Tramontano, Göran Sperber

P57 Ferroportin restricts HIV-1 infection in sickle cell disease

Namita Kumari, Tatiana Ammosova, Sharmeen Diaz, Patricia Oneal, Sergei Nekhai

P58 APOBEC3G modulates the response to antiretroviral drugs in humanized mice

Audrey Fahrny, Gustavo Gers-Huber, Annette Audigé, Roberto F. Speck, Anitha Jayaprakash, Ravi Sachidanandam, Matt Hernandez, Marsha Dillon-White, Viviana Simon

P59 High-throughput epigenetic analysis of evolutionarily young endogenous retrovirus presents in the mule deer *(Odocoileus hemionus*) genome

Tomas Hron, Helena Farkasova, Daniel Elleder

P60 Characterisation of the expression of novel endogenous retroviruses and immune interactions in a macaque model

Neil Berry, Emmanuel Maze, Claire Ham, Neil Almond, Greg Towers, Robert Belshaw

P61 HIV-1 restriction by orthologs of SERINC3 and SERINC5

Patrícia de Sousa-Pereira, Joana Abrantes, Massimo Pizzato, Pedro J. Esteves, Oliver T. Fackler, Oliver T. Keppler, Hanna-Mari Baldauf

P62 TRIM19/PML restricts HIV infection in a cell type-dependent manner

Bianca Volkmann, Tanja Kahle, Kristin Eissmann, Alexandra Herrmann, Sven Schmitt, Sabine Wittmann, Laura Merkel, Nina Reuter, Thomas Stamminger, Thomas Gramberg

P63 Recent invasion of the mule deer genome by a retrovirus

Helena Farkasova, Tomas Hron, Daniel Elleder

P64 Does the antiviral protein SAMHD1 influence mitochondrial function?

Ilaria Dalla Rosa, Kate Bishop, Antonella Spinazzola, Harriet Groom

P65 cGAMP transfers intercellularly via HIV-1 Env-mediated cell–cell fusion sites and triggers an innate immune response in primary target cells

Shuting Xu, Aurélie Ducroux, Aparna Ponnurangam, Sergej Franz, Gabrielle Vieyres, Mathias Müsken, Thomas Zillinger, Angelina Malassa, Ellen Ewald, Veit Hornung, Winfried Barchet, Susanne Häussler, Thomas Pietschmann, Christine Goffinet

P66 Pre-infection transcript levels of *FAM26F* in PBMCS inform about overall plasma viral load in acute and postacute phase after SIV-infection

Ulrike Sauermann, Aneela Javed, Nicole Leuchte, Gabriela Salinas, Lennart Opitz, Christiane Stahl-Hennig, Sieghart Sopper

P67 Sequence-function analysis of three T cell receptors targeting the HIV-1 p17 epitope SLYNTVATL

Christiane Mummert, Christian Hofmann, Angela G. Hückelhoven, Silke Bergmann, Sandra M. Müller-Schmucker, Ellen G. Harrer, Jan Dörrie, Niels Schaft, Thomas Harrer

P68 An immunodominant region of the envelope glycoprotein of small ruminant lentiviruses may function as decoy antigen

Laure Cardinaux, M.-L. Zahno, H.-R. Vogt, R. Zanoni, G. Bertoni

P69 Impact of immune activation, immune exhaustion, broadly neutralising antibodies and viral reservoirs on disease progression in HIV-infected children

Maximilian Muenchhoff, Philip Goulder, Oliver Keppler

Topic 9: Novel antiviral strategies

P70 Identification of natural compounds as new antiviral products by bioassay-guided fractionation

Alexandra Herrmann, Stephanie Rebensburg, Markus Helfer, Michael Schindler, Ruth Brack-Werner

P71 The PPARG antagonism disconnects the HIV replication and effector functions in Th17 cells

Yuwei Zhang, Huicheng Chen, Delphine Planas, Annie Bernier, Annie Gosselin, Jean-Pierre Routy, Petronela Ancuta

P72 Characterisation of a multiresistant subtype AG reverse transcriptase: AZT resistance, sensitivity to RNase H inhibitors and inhibitor binding

Birgitta Wöhrl, Anna Schneider, Angela Corona, Imke Spöring, Mareike Jordan, Bernd Buchholz, Elias Maccioni, Roberto Di Santo, Jochen Bodem, Enzo Tramontano, Kristian Schweimer

P73 Insigths into the acetylation pattern of HDAC inhibitors and their potential role in HIV therapy

Christian Schölz, Brian Weinert, Sebastian Wagner, Petra Beli, Yasuyuki Miyake, Jun Qi, Lars Jensen, Werner Streicher, Anna McCarthy, Nicholas Westwood, Sonia Lain, Jürgen Cox, Patrick Matthias, Matthias Mann, James Bradner, Chunaram Choudhary

P74 HPV-derived and seminal amyloid peptides enhance HIV-1 infection and impair the efficacy of broadly neutralising antibodies and antiretroviral drugs

Marcel Stern, Oliver T. Keppler

P75 D(−)lentiginosine inhibits both proliferation and virus expression in cells infected by HTLV-1 in vitro

Elena Valletta, Caterina Frezza, Claudia Matteucci, Francesca Marino-Merlo, Sandro Grelli, Anna Lucia Serafino, Antonio Mastino, Beatrice Macchi

P76 HIV-1 resistance analyses of the Cape Winelands districts, South Africa

Sello Mikasi, Graeme Jacobs, Susan Engelbrecht

Topic 10: Recent advances in HIV vaccine development

P77 Induction of complex retrovirus antigen-specific immune responses by adenovirus-based vectors depends on the order of vector administration

Meike Kaulfuß, Sonja Windmann, Wibke Bayer

P78 Direct impact of structural properties of HIV-1 Env on the regulation of the humoral immune response

Rebecca Heß, Michael Storcksdieck gen. Bonsmann, Viktoria Stab, Carsten Kirschning, Bernd Lepenies, Matthias Tenbusch, Klaus Überla

P79 Lentiviral virus-like particles mediate gerenration of T-follicular helper cells *in vitro*

Anne Kolenbrander, Klaus Überla, Vladimir Temchura

P80 Recruitment of HIV-1 Vpr to DNA damage sites and protection of proviral DNA from nuclease activity

Kenta Iijima, Junya Kobayashi, Yukihito Ishizaka

## Oral presentations

### Session 1: Entry & uncoating

#### O1 Host cell polo-like kinases (PLKs) promote early prototype foamy virus (PFV) replication

##### Irena Zurnic^1,2^, Sylvia Hütter^2^, Ute Lehmann^3^, Nicole Stanke^2^, Juliane Reh^4^, Tobias Kern^2,4^, Fabian Lindel^2,4^, Gesche Gerresheim^2^, Martin Hamann^2,4^, Erik Müllers^2,4^, Paul Lesbats^5^, Peter Cherepanov^5^, Erik Serrao^6^, Alan Engelman^6^, Dirk Lindemann^2^

###### ^1^Molecular Virology and Gene Therapy, Molecular Medicine, Leuven, Belgium; ^2^Technical University Dresden, Molecular Virology, Dresden, Germany; ^3^Potsdam University, Potsdam, Germany; ^4^Center for regenerative therapies Dresden, Dresden, Germany; ^5^The Francis Crick Institute, London, Great Britain; ^6^Dana Farber Cancer Institute, Boston, MA United States

**Correspondence:** Irena Zurnic

*Retrovirology* 2016, **13(Suppl 1)**: O1

**Question:** Foamy viruses (FV), and in particular PFV, have emerged in recent years as attractive gene therapy vector candidates. Since the lack of knowledge on molecular events in FV replication is a major hurdle for broader usage of foamy virus vectors, we aimed at elucidating PFV biology by investigating interactions of its capsid protein, Gag, with host cell components.

**Methodology and result:** To this end, we identified members of the mammalian PLK family as PFV Gag interactants in a commercial yeast-two-hybrid (Y2H) screen and validated these results in detailed Y2H experiments for PLK1–3. In the yeast system, the intact PLK kinase and substrate recognition motifs were required for interactions with PFV Gag, in which a unique S_224_-T-P_226_ motif served as a PLK binding determinant. PFV Gag mutants harbouring alanine substitutions of STP residues (iSTP) or phosphomimetic mutations of the T_225_ (pmSTP) failed to interact with PLK1–3 in yeast. These findings were corroborated by colocalization studies of ectopically expressed, fluorescently tagged proteins in mammalian cells, where mCherry-tagged PFV Gag was able to recruit eGFP-tagged PLK1 and 2 to condensed mitotic chromatin in an STP motif-dependent manner.

When characterizing PFV virions containing wild type or STP mutant Gag proteins, we observed that the mutations did not interfere with particle assembly, release or reverse transcription, but led to a 70 % titer reduction relative to wild type in single-round infection experiments. These replication defects became more prominent in the replication-competent PFV context. Therefore, the lack of Gag STP mutant interaction with PLK proteins upon viral entry into host cells was likely underlying this replication deficit. This hypothesis was strengthened by the finding that enzymatic PLK inhibition in host cells during transduction with wild type PFV mimicked the replication phenotype of PFV STP mutants. In addition to the overall reduced infectivity of the mutants, we also observed that the STP mutations in particle-associated Gag lead to differential sensitivity to integrase inhibition by dolutegravir and resulted in decreased integration efficiency.

**Conclusions:** Taken together, our results demonstrate that PLK proteins influence PFV replication by virtue of their interaction with the Gag protein, ensuring timely and efficient transduction.

#### O2 A novel entry/uncoating assay reveals the presence of at least two species of viral capsids during synchronized HIV-1 infection

##### Claire Da Silva Santos, Kevin Tartour, Andrea Cimarelli

###### CIRI, Lyon, France

**Correspondence:** Andrea Cimarelli

*Retrovirology* 2016, **13(Suppl 1)**: O2

After viral-to-cellular membrane fusion, a nucleoprotein complex enclosing the viral genome and composed of viral as well as cellular proteins is released in the cell cytoplasm. The behavior of viral cores once inside the cell is notoriously difficult to apprehend especially given the metamorphic nature of these structures over time. To better understand the behavior of HIV-1 capsids, we have developed a novel assay that we named EURT, forEntry/Uncoating assay based on core-packagedRNA availability andTranslation. This novel entry-uncoating assay is based on the degree of exposure of a virion core-packaged mRNA reporter to the translation machinery of the target cell and it provides a measure of the status of viral capsids, as reporter RNA translation is prevented in hyperstable viral capsids. Using EURT, we highlight here that two kinds of viral cores coexist during HIV-1 infection: one that we define as open, in which the viral genome is readily accessible to the cytoplasmic environment and to the translation machinery and another that we define as close, in which access to the RNA is prevented until viral capsid is destabilized. The results we have obtained so far indicate that the former species represents a dead-end product of infection, likely derived from improper or inefficient assembly of infectious virion units. Interestingly, IFNα that negatively impacts HIV-1 replication increases the proportion of open viral cores to the detriment of closed ones, suggesting a core-destabilizing activity driven by interferon-regulated proteins. These and other results examining the relationship between core opening and reverse transcription will be presented.

Work in our laboratory is supported by the ANRS and Sidaction.

#### O3 Dynamics of nuclear envelope association and nuclear import of HIV-1 complexes

##### Rya Burdick, Jianbo Chen, Jaya Sastri, Wei-Shau Hu, Vinay Pathak

###### National Cancer Institute, Frederick, MD, United States

**Correspondence:** Vinay Pathak

*Retrovirology* 2016, **13(Suppl 1)**: O3

HIV-1 must travel through the cytoplasm to reach the nuclear envelope (NE) of an infected cell, transport through a nuclear pore to enter the nucleus, and integrate its genome into the chromosomal DNA of the host cell. Recently, we have labeled HIV-1 virions with APOBEC3F fused to yellow fluorescent protein (A3F-YFP), which remains stably associated with viral complexes, and visualized the viral complexes in infected cells to gain new insights into the early stages of viral replication. We showed that reverse transcription is not required for nuclear import of HIV-1 complexes, indicating that alterations in the viral capsid (CA) structure that accompany reverse transcription are dispensable for their nuclear import. We found that HIV-1 CA mutations that altered the stability of the viral core significantly reduced the association of viral complexes with the nuclear envelope (NE) and their nuclear import. In addition, we found that nuclear viral complexes remain near NE and are not randomly distributed in the nuclei.

The dynamics of HIV-1 association with the NE and nuclear import in infected cells are not well understood. To gain insights into the dynamics of HIV-1 association with the NE, we analysed A3F-labeled HIV-1 complexes in living cells, and observed that most contacts between HIV-1 and NE form transient associations while few form stable associations, which are essential for nuclear import. Furthermore, HIV-1 capsid and host Nup358 played critical roles in forming the stable associations. Additionally, we observed the translocation of viral complexes from the cytoplasm to the NE during nuclear import. We determined that viral complexes have long residence times at the NE prior to import. After import, viral complexes exhibit a brief fast phase as they move away from the point of entry, followed by a long slow phase, suggesting they are associated with chromatin and/or other nuclear macromolecules. These studies provide novel insights into the dynamics of HIV-1 NE association, nuclear import, and nuclear movements.

#### O4 Human papillomavirus protein E4 potently enhances the susceptibility to HIV infection

##### Oliver T. Keppler

###### LMU Munich, Max von Pettenkofer-Institut, Virology, Munich, Germany

**Correspondence:** Oliver T. Keppler

*Retrovirology* 2016, **13(Suppl 1)**: O4

Sexually transmitted infections of the anogenital tract are important cofactors for HIV transmission, and acute infections by mucotropic human papillomaviruses (HPV) enhance the risk of HIV acquisition. Little is known about the molecular mechanisms involved in this increased susceptibility.

Here we show that the abundant E4 protein, which is encoded by both oncogenic and non-oncogenic HPV types, drastically enhances HIV infection. E4 is expressed in HPV-infected, ultimately disintegrating keratinocytes in the outermost cell layers of the anogenital mucosa. *N*-terminally cleaved forms of E4 self-assembled into cationic, intermediate amyloid fibrils that captured and concentrated cell-free HIV particles, protecting their infectivity and promoting their envelope-independent binding and envelope-dependent fusion to primary target cells. E4 drastically lowered the virus titer required for productive HIV infection in lymphoid organ cultures ex vivo and infection enhancement occurred efficiently in vaginal fluid. Moreover, HIV-permissive target cells were found to be recruited into HPV-induced lesions in the anogential mucosa.

Thus the concept emerges that aggregating cleavage products originating from body fluids, including semen, and from co-infecting pathogens can alone or in combination modulate the susceptibility to sexual transmission of HIV. In conjunction with the observational epidemiological evidence these findings provide a molecular rationale to extend anti-HPV vaccine programs to individuals at risk for HIV acquisition. The development of a new generation of broad-range vaccines that protect from infection with all circulating, oncogenic and non-oncogenic mucosal HPV types may lower the global incidence of HIV infection.

### Session 2: Reverse transcription & integration

#### O5 Structure and function of HIV-1 integrase post translational modifications

##### Karine Pradeau^1^, Sylvia Eiler^1^, Nicolas Levy^1^, Sarah Lennon^2^, Sarah Cianferani^2^, Stéphane Emiliani^3^, Marc Ruff^1^

###### ^1^IGBMC, Integrative structural Biology, Illkirch, France; ^2^Institut Pluridisciplinaire Hubert Curien, Strasbourg, France; ^3^Institut Cochin, Paris, France

**Correspondence:** Marc Ruff

*Retrovirology* 2016, **13(Suppl 1)**: O5

After retroviral infection of a target cell, during the early phase of replication, the HIV-1 genomic viral RNA is reverse transcribed by the viral reverse transcriptase (RT) to generate the double-stranded viral DNA that interact with viral and cellular proteins to form the pre-integration complex (PIC). Viral integrase (IN) is a key component of the PIC and is involved in several steps of replication notably in reverse transcription, nuclear import, chromatin targeting and integration. Viral components such as IN cannot perform these functions on their own and need to recruit host cell proteins to efficiently carry out the different processes. IN is a disordered protein showing high inter-domain flexibility. This flexibility accounts for IN ability to interact with multiple partners allowing its multiple functions in viral replication. Yet the molecular mechanisms and dynamics of these processes, the role of cellular co-factors as well as of post-translational modifications remain largely unknown.

To produce and purify proteins participating in these transient macromolecular complexes we develop new technologies for high molecular weight transient complexes production as well as for functional and structural analysis. We demonstrated that the low solubility and inter-domain flexibility can be circumvented by forming stable and specific complexes with substrates such as DNA or protein co-factors and by post-translational modifications (PTMs). We purified HIV-1 IN alone and in complex with viral and cellular proteins produced in *E. coli*, insect and mammalian cells (Levy et al. 2016, Nature Communications 7:10932). Comparison of IN purified from *E. coli*, insect and mammalian cells production showed that IN purified from mammalian cell production showed higher solubility, increased 3′ processing activity as well as 5 PTMs (one phosphorylation and four acetylation). Mutant of the phosphorylated and acetylated sites were generated and their effect on viral replication and 3′ processing are analysed. Structural analysis of acetylation in the IN catalytic core domain suggest that acetylation participate in the modulation of IN multimerization.

#### O6 Regulation of retroviral integration by RNA polymerase II associated factors and chromatin structure

##### Vincent Parissi

###### CNRS, UMR5234 MFP Lab, Bordeaux, France

**Correspondence:** Vincent Parissi

*Retrovirology* 2016, **13(Suppl 1)**: O6

HIV-1 integration occurs in highly Pol II transcribed and spliced regions of the chromatin thanks to the interaction between the retroviral intasome and the cellular tethering factor LEDGF/p75. These regions of the host genome are enriched in transcription and remodeling factors that are expected to modulate the chromatin access to the incoming intasome and its functional association with the targeted nucleosome. Since this final step has been shown to be regulated by both intasomes and chromatin structure we investigated these regulation processes focusing on the analysis of the IN/nucleosome interaction and on the role of the cellular proteins associated with the Pol II transcription apparatus. We found that HIV-1 IN specifically binds to the amino-terminal tail of human histone H4, a major component of the nucleosome. This interaction was found required for optimal association and integration onto nucleomes. Functional and structural analysis of this interaction led us to validate the presence of an unedited histone tail binding motif in the CTD of HIV-1 IN that behaves as an ancestral SH3/β-chromobarrel chromatin-reader domain and point out the critical role of the IN/H4 association during the retroviral integration process. Additionally, the analysis of the role of Pol II associated remodeling factors on this functional association led us to found that FACT (facilitates chromatin transcription) complex, a chromatin remodeler associated with Pol II and recently reported to bind LEDGF/p75, can regulate the access to the nucleosome and histone tails to the incoming intasomes. Mechanistic studies indicate that FACT generates partially dissociated nucleosomes structures that are highly favored substrates for HIV-1 integration. This partial nucleosome dissociation decreases the chromatin density in the vicinity of the integration site and, thus, allows the final association between intasomes and the targeted nucleosome. Consequently, our work highlights new host/pathogen interactions that could constitute novel and attractive targets for future potential therapeutic applications in addition to provide a better understanding of this crucial integration step of the retroviral replication.

### Session 3: Transcription and latency

#### O7 A novel single-cell analysis pipeline to identify specific biomarkers of HIV permissiveness

##### Sylvie Rato^1^, Antonio Rausell^2^, Miguel Munoz^1^, Amalio Telenti^3^, Angela Ciuffi^1^

###### ^1^Institute of Microbiology, University Hospital Center and University of Lausanne, Lausanne, Switzerland; ^2^Imagine Institute, Paris Descartes University, Paris, France; ^3^J. Craig Venter Institute, La Jolla, CA, United States

**Correspondence:** Sylvie Rato

*Retrovirology* 2016, **13(Suppl 1)**: O7

**Background:** Cellular permissiveness to HIV infection is highly heterogeneous across individuals, as well as across cells from the same individual. To investigate the major source of this difference and identify biomarkers, we developed a novel pipeline based on single-cell analysis, where (i) cellular heterogeneity was evaluated at transcriptome level, and (ii) HIV permissiveness was correlated to cell surface protein expression.

**Methods:** Activated CD4+ T cells from a high and low permissive donor were used for single-cell RNA-seq analysis (fluidigm C1™ technology).

Activated cells from the high permissive donor were infected with a GFP encoding HIV-based vector. Expression of 332 cell surface proteins (LegendScreen™) was assessed by FACS and correlated with GFP expression.

Candidate biomarkers were validated in activated cells sorted according to their protein expression level and infected by HIV vector.

**Results:** Transcriptomic profiles of 85 high and 81 low permissive single cells were successfully obtained. Transcriptional heterogeneity observed at single-cell level identified TCR-mediated cell activation as a major determinant of cellular heterogeneity.

Cell surface expression analysis of 332 proteins identified 76 candidates correlating with successful HIV infection, including CD25, a typical activation marker.

Candidate biomarkers of HIV permissiveness were selected based on correlations between gene expression and activation (single-cell RNA-Seq) and between surface protein expression and HIV permissiveness (LegendScreen™), and tested for their ability to capture permissive cells.

Eleven candidate biomarkers were successfully validated, showing enrichment of HIV permissive cells.

**Conclusions:** In this study, we developed a single-cell pipeline to investigate cell heterogeneity and identify gene candidates affecting HIV permissiveness. Our data showed that, at the single-cell level, the status of cellular activation was the major driver of cell heterogeneity towards HIV permissiveness. Moreover, we identified several cell surface biomarkers characterizing the HIV permissive cell. This single-cell analysis pipeline represents a valuable tool for biomarker identification.

**Acknowledgements**

FP7 European grant n°305762/Hit Hidden HIV and Swiss SNF grant 166412.

#### O8 A capsid-dependent integration program linking T cell activation to HIV-1 gene expression

##### Alexander Zhyvoloup^1^, Anat Melamed^2^, Ian Anderson^1^, Delphine Planas^3^, Janos Kriston-Vizi^4^, Robin Ketteler^4^, Chen-Hsuin Lee^1^, Andy Merritt^5^, Petronela Ancuta^3^, Charles Bangham^2^, Ariberto Fassati^1^

###### ^1^University College London, Infection, London, Great Britain; ^2^Imperial College, Medicine, London, Great Britain; ^3^University of Montreal, Microbiology & Infection, Montreal, Great Britain; ^4^University College London, LMCB, London, Great Britain; ^5^MRC Technology, Centre for Therapeutic discovery, London, Great Britain

**Correspondence:** Ariberto Fassati

*Retrovirology* 2016, **13(Suppl 1)**: O8

To identify key steps of the HIV-1 life cycle that are dependent on capsid (CA), we have developed differential high throughput chemical screening. The CA point mutation N74D makes HIV-1 independent of several host factors. Taking advantage of this phenotype, CD4+ T cells were co-infected with two HIV-1 vectors (WT-GFP and N74D-mCherry), which were identical except for the CA N74D mutation. Compound libraries were screened to find molecules that selectively inhibited infection of WT over N74D, which, by implication, should affect directly or indirectly the interaction between host factors and HIV-1 CA.

We found that digoxin selectively inhibited infection of WT in both CD4 T cell lines and primary memory CD4 T cells, repressing HIV-1 gene expression. The antiretroviral activity of digoxin was dependent on the Na/K ATPase. To identify the mechanism of digoxin selectivity, we infected CD4 T cells with WT or N74D virus in the presence of digoxin then analysed in parallel the cellular transcriptional profile by RNAseq and integration site selection by deep sequencing. RNAseq showed that digoxin up-regulated 221 genes and down-regulated 336 genes ≥fourfold. Within the *up*-*regulated* genes, the main biological functions affected by digoxin were regulation of cell cycle, chromatin remodeling, RNA processing, cell survival. Within the *down*-*regulated* gene group, the main biological functions impinged by digoxin were antigen presentation, T cell activation and metabolism. Two main gene networks down-regulated by the drug were CD40L and CD38 markers of T cell activation.

We examined >400,000 unique integration sites and discovered that WT virus had a stronger bias relative to N74D virus to integrate within or near genes susceptible to down-regulation by digoxin. Of those, integration within or near genes involved in T cell activation was twofold more frequent for WT virus than N74D virus and 3.8-fold more frequent than integration near any gene. Thus WT virus is more sensitive to digoxin than N74D because it integrates more frequently within or near genes down-regulated by the drug. We discovered a functional connection between integration preference and T cell activation and metabolism that is dependent on CA, which may affect the establishment of latency and the control of viral reactivation.

#### O9 Characterisation of new RNA polymerase III and RNA polymerase II transcriptional promoters in the Bovine Leukemia Virus genome

##### Anthony Rodari^1^, Benoit Van Driessche^1^, Mathilde Galais^1^, Nadége Delacourt^1^, Sylvain Fauquenoy^1^, Caroline Vanhulle^1^, Anna Kula^1^, Arsène Burny^2^, Olivier Rohr^3^, Carine Van Lint^1^

###### ^1^University of Brussels, Molecular Virology, Gosselies, Belgium; ^2^University of Brussels, Laboratory of Experimental Hematology, Brussels, Belgium; ^3^University of Strasbourg, Institut Universitaire de Technologie (IUT) Louis Pasteur de Schiltigheim, Schiltigheim, France

**Correspondence:** Anthony Rodari

*Retrovirology* 2016, **13(Suppl 1)**: O9

Bovine leukemia virus (BLV), the etiologic agent of enzootic bovine leucosis, is a B-lymphotropic oncogenic retrovirus closely related to the human T cell leukemia virus I and II (HTLV-I and II). It is widely accepted that BLV latency, due to the RNA polymerase II (RNAPII) 5′LTR-driven transcriptional and epigenetic repression, is a viral strategy used to escape from the host immune system and contribute to tumor development. However, by deep sequencing and bioinformatics analysis, a highly expressed BLV micro-RNA (miRNA) cluster has been recently reported, suggesting that the silencing dogma in BLV transcriptional regulation is only partially correct. In addition, these viral miRNAs are produced through a non-canonical process, involving RNA polymerase III (RNAPIII).

In this report, we used chromatin immunoprecipitation assays to demonstrate the in vivo recruitment of a *bona fide* RNAPIII complex to the BLV miRNA cluster both in BLV-latently infected cell lines and in ovine BLV-infected primary cells, through a canonical type 2 RNAPIII promoter. In addition, by specific knockdown of the RPC6 RNAPIII subunit, we showed a direct functional link between RNAPIII transcription and BLV miRNAs expression. Furthermore, in BLV-latently infected cell lines and in ovine BLV-infected primary cells, we showed that both the tumor- and the quiescent-related isoforms of RPC7 RNAPIII subunits were recruited to the miRNA cluster, consistent with previous studies showing that the viral miRNAs are transcribed at all stages of BLV disease. Epigenetically, we demonstrated that the BLV miRNA cluster was enriched in positive epigenetic marks in agreement with the high expression level of the viral miRNAs previously reported. Interestingly, we also demonstrated the in vivo recruitment of RNAPII at the 3′LTR/host genomic junction, associated with positive epigenetic marks. Functionally, we showed that the BLV LTR exhibited a strong antisense promoter activity and identified *cis*-acting elements of an RNAPII-dependent promoter. Finally, we provided evidence for an in vivo collision between RNAPIII and RNAPII convergent transcriptions.

Taken together, our results provide new insights into alternative ways used by BLV to counteract silencing of the viral 5′LTR promoter.

#### O10 Tissue-specific dendritic cells differentially modulate latent HIV-1 reservoirs

##### Thijs van Montfort^1^, Renee van der Sluis^1^, Dave Speijer^2^, Ben Berkhout^1^

###### ^1^Academic Medical Center, Medical Microbiology, Amsterdam, Netherlands; ^2^Academic Medical Center, Medical Biochemistry, Amsterdam, Netherlands

**Correspondence:** Thijs van Montfort

*Retrovirology* 2016, **13(Suppl 1)**: O10

**Method:** Early after HIV-1 acquisition, cellular viral reservoirs are formed containing latent, but infectious, HIV-1. Cells belonging to these latent reservoirs can, when activated, reestablish new viral infections in the absence of antiretroviral therapy (ART). How latently infected cells are activated in patients is still unclear. To examine whether cellular signaling between cells can activate latent HIV-1, we co-cultured tissue-specific immune cells, including a large panel of dendritic cell subsets with latently infected cells. Next, we measured reversal of HIV-1 latency using our in vitro latency model. Moreover, by using specific signaling pathway inhibitors we determined which intracellular signaling pathways are important for reversion: making latent HIV replication competent upon cell–cell contact.

**Results:** Our results demonstrate that blood or genital tract dendritic cells do not activate latent provirus in effector T-cells, whereas gut or lymphoid dendritic cells induce virus production from latently infected effector T-cells. Dendritic cells were also able to activate latent HIV-1 in resting T cells from patients as measured with the virus outgrowth assay. We could show that activation was not triggered via classical T-cell stimulation pathways, but that activation occurred via the mTORC1 pathway.

**Conclusion:** In this study, we show that HIV-1 provirus residing in the effector T-cells is activated from latency by tissue-specific dendritic cell subsets and other immune cells with remarkably different efficiencies. Our results suggest that the observed rapid depletion of T cells in the gut can be attributed to the large quantities of gut dendritic cells that constantly promote virus propagation from latently infected cells.

### Session 4: RNA trafficking & packaging

#### O11 A novel ***cis***-acting element affecting HIV replication

##### Bo Meng^1^, Andrzej Rutkowski^1^, Neil Berry^2^, Lars Dölken^1,3^, Andrew Lever^1^

###### ^1^University of Cambridge, Division of Infectious Diseases, Cambridge, Great Britain; ^2^National Institute for Biological Standards and Control, Division of Virology, Potters Bar, Great Britain; ^3^Julius-Maximilians-Universität Würzburg, Institute for Virology and Immunbiology, Würzburg, Germany

**Correspondence:** Bo Meng

*Retrovirology* 2016, **13(Suppl 1)**: O11

**Background:** HIV RNA is known to contain a large number of *cis*-acting sequences such as the TAR stem loop, packaging signal and the Rev responsive element with which HIV controls its lifecycle.

**Methods:** Bioinformatics analysis across HIV sequences has identified regions with high sequence homology to motifs associated with subcellular trafficking of RNA in other systems. The motifs were synonymously mutated in HIV and viral replication kinetics examined.

**Results:** Upon disruption of these elements, we observed a phenotypic effect on virus replication manifest as a slow virus growth rate but showed cell type specificity, being most apparent in physiologically relevant T cells but not commonly used cell lines. This effect seems to act at the early stage of the virus life cycle as the overall production of HIV-1 Gag protein is reduced leading to a diminished amount of intracellular viral protein.

**Conclusions:** Studies to date implicate a transcriptional or post-transcriptional defect involving members of the ESCRT group of cellular proteins.

#### O12 Tolerance of HIV’s late gene expression towards stepwise codon adaptation

##### Thomas Schuster, Benedikt Asbach, Ralf Wagner

###### ^1^Institute of Medical Microbiology and Hygiene, University of Regensburg, Regensburg, Germany

**Correspondence:** Thomas Schuster

*Retrovirology* 2016, **13(Suppl 1)**: O12

Different organisms show differences in the frequency of occurrence of synonymous codons (codon usage bias). In contrast to humans, HIV uses in general the most A-rich codon for a certain amino acid. Codon usage influences protein production on various levels, starting from gene expression over RNA metabolism to translation.

Previous studies of our group showed that adapting the *gag* gene to human codon usage (huGag) led not only to a significantly increased protein production but also caused independency of Rev, an accessory protein of HIV which mediates the export of unspliced and incompletely spliced HIV mRNAs. The aim of this work is to gain insight into the effects of codon adaptation on gag expression, especially regarding length and position. For this, subgenomic gag reporter constructs were generated that extend the humanized part of the gene stepwise from 5′end to 3′end as well as from 3′end to 5′end. Those constructs were then transfected into HEK293T cells. Gag expression was investigated on protein level by p24 ELISA as well as on RNA level by Northern blot analysis and qPCR.

It became apparent that humanization of the very 5′ end is necessary for enhanced protein production and Rev-independent expression. Moreover, increasing the length of the humanized sequence starting from the 5′ end directly correlated with p24 levels. Contrary to that, such a correlation is lacking for constructs humanized progressively in 3′ to 5′ direction. Interestingly, even humanization of the whole *gag* except of the 5′part remained Rev-dependent and thus did not show enhanced gag expression. Inhibition of Rev-mediated export with LMB confirmed the Rev-independency of huGag and showed that Rev-dependency decreased with increasing length of the optimized sequence part. Relative quantification of the RNA levels corroborated the results obtained on protein level. The existence of cryptic splicing products could be ruled out by Northern Blot analysis.

In summary, codon adaptation of the 5′ part of HIV gag seems to be necessary for enhanced and Rev-independent Gag expression. Clarification of the underlying molecular mechanisms could help to understand how HIV benefits from the deviant codon usage.

### Session 5: Assembly & release

#### O13 Importance of the tax-inducible actin-bundling protein fascin for transmission of human T cell leukemia virus Type 1 (HTLV-1)

##### Christine Gross^1^, Veit Wiesmann^2^, Martina Kalmer^1^, Thomas Wittenberg^2^, Jan Gettemans^3^, Andrea K. Thoma-Kress^1^

###### ^1^Institute of Clinical and Molecular Virology, Friedrich-Alexander-Universität Erlangen-Nuremberg, Erlangen, Germany; ^2^Fraunhofer Institute for Integrated Circuits IIS, Erlangen, Germany; ^3^Ghent University, Department of Biochemistry, Faculty of Medicine and Health Sciences, Campus Rommelaere, Ghent, Belgium

**Correspondence:** Christine Gross

*Retrovirology* 2016, **13(Suppl 1)**: O13

Transmission of Human T-cell leukemia virus type 1 (HTLV-1) between CD4^+^ T-cells requires cell–cell contacts and remodeling of the host cell cytoskeleton. The viral transactivator Tax is crucial for formation of the virological synapse (VS), a specialized cell–cell contact. At the VS, polarized budding of virions into synaptic clefts and transfer of viral biofilms to target cells takes place. Furthermore, HTLV-1 is transmitted via cellular protrusions. The actin-bundling protein Fascin is Tax-dependently upregulated in HTLV-1-infected T-cells and important for the formation of protrusive structures. Here, we report that Fascin is required for HTLV-1 transmission. Repression of endogenous Fascin by short hairpin RNAs or delocalization of Fascin by nanobodies impaired virus transmission and release using single-cycle replication-dependent HTLV-1 reporter vectors in 293T cells. Beyond, HTLV-1 release and cell-to-cell transmission were enhanced by Tax-induced Fascin in Jurkat T-cells in co-culture with Raji/CD4^+^ B-cells. In chronically HTLV-1-infected T-cells, virus release, transfer of gag p19 to target cells and transactivation of co-cultured reporter T-cells was decreased upon knockdown of Fascin. Spotting the mechanism, confocal laser scanning microscopy revealed that Fascin and actin accumulate at cell–cell contacts suggesting that Fascin enhances T-cell conjugate formation. However, Fascin was not necessary for Tax-induced T-cell aggregation as exploited by automatic image analysis and flow cytometry-based aggregation assays. Yet, Fascin potentially regulates dissemination of infected cells as knockdown of Fascin reduced adhesion of HTLV-1-infected MT-2 cells. Immunofluorescence analysis of cell–cell-contacts between HTLV-1-positive MS-9 cells co-cultured with Jurkat T-cells revealed the following: (1) short Fascin-containing protrusions extend from infected cells and clutch target cells, (2) Fascin and gag localize in long-distance cellular protrusions in close proximity or partially co-localize, and (3) clusters of Fascin are interspersed by clusters of gag reminiscent of viral biofilms. Taken together, we found that endogenous and Tax-induced Fascin is crucial for HTLV-1 transmission, potentially by redirecting viral proteins to budding sites.

#### O14 Lentiviral nef proteins antagonize TIM-mediated inhibition of viral release

##### Minghua Li^1^, Eric O. Freed^2^, Shan-Lu Liu^1^

###### ^1^The Ohio State University, Center for Retrovirus Research, Columbus, GA, United States; ^2^National Cancer Institute, HIV Dynamics and Replication, Frederick, MD, United States

**Correspondence:** Shan-Lu Liu

*Retrovirology* 2016, **13(Suppl 1)**: O14

We recently reported that the T cell immunoglobulin and mucin domain (TIM) proteins inhibit release of HIV-1 and other enveloped viruses by interacting with virion- and cell-associated phosphatidylserine (PS) (Li et al., PNAS 111, 2014). In this study, we demonstrate that the Nef proteins of HIV-1 and other lentiviruses antagonize TIM-mediated restriction. We show that TIM-1 exhibits stronger inhibition of the release of Nef-deficient relative to Nef-expressing HIV-1 particles and that ectopic expression of Nef relieves this restriction. Consistent with this finding, knockdown of endogenous TIM-3 in human PBMCs effectively enhances the production of Nef-deficient HIV-1 particles. HIV-1 Nef does not appear to downregulate TIM-1 expression on the cell surface, nor does it disrupt TIM-1 incorporation into HIV-1 virions. Interestingly, we observed that coexpression of SERINC3 and SERINC5 potentiates TIM-1 inhibition of HIV-1 release, and that depletion of SERINC proteins in viral-producer cells rescues TIM-mediated inhibition of HIV-1 release. These results suggest that SERINCs are involved in TIM-mediated restriction of HIV-1 release. In addition to HIV-1 Nef, the Nef proteins of simian immunodeficiency virus (SIV) strains and HIV-2 also antagonize the antiviral activity of TIM-1, suggesting an evolutionarily conserved role of the lentiviral *nef* gene in antagonizing TIMs. Collectively, our work reveals a new role for lentiviral Nef in antagonizing TIM, and highlights a complex interplay between lentiviral Nef and cellular restriction by TIMs and SERINCs.

### Session 6: Pathogenesis & evolution

#### O15 SEVI and semen prolong the half-life of HIV-1

##### Janis Müller, Jan Münch

###### ^1^Institute of Molecular Virology, Ulm, Germany

**Correspondence:** Janis Müller

*Retrovirology* 2016, **13(Suppl 1)**: O15

Human Immunodeficiency Virus Type 1 (HIV-1), the causative agent of Acquired Immunodeficiency Syndrome (AIDS), has currently infected 35 million people and caused 1.5 million deaths in 2013 with no cure available. 85 % of all new infections occur following sexual intercourse where semen is the main vector of HIV-1 transmission. Instead of being a passive carrier, semen enhances HIV infectivity, an activity that is attributed to amyloid fibrils present in semen. These fibrils self-assemble from peptides derived from prostatic acidic phosphatase (PAP) or semenogelins where SEVI (Semen-derived Enhancer of Virus Infection) formed from PAP248–286 is the best characterised. The cationic surface of semen amyloid allows capturing negatively charged HIV-1 virions and increases attachment to and thus infection of target cells. Interestingly, HIV-1 particles are relatively labile and have a reported half-life of only a few hours in serum, probably due to gp120 shedding and membrane rupture induced by shearing forces. We hypothesized that amyloid, by binding and concentrating, may stabilize virions resulting in a prolonged infectious half-life. We thus performed HIV-1 decay kinetics in buffer, and in the presence of SEVI or human semen at 37 °C. We found that the infectious half-life of HIV-1 in buffer was 2.5 ± 1.3 h, independent on the virus concentration used. Incubation with physiological concentrations of SEVI increased infection rates and additionally extended the viral half-life to 12.1 ± 2.7 h (p < 0.0001). This effect was observed using lab adapted NL4-3 as well as transmitted/founder (T/F) HIV-1 variants. Furthermore, HIV-1 incubated in semen reached half-lives up to 10.2 ± 3.2 h (p < 0.0001). This effect was abrogated by depleting semen of amyloid, suggesting it accounts for the observed phenomenon. Normalizing for initial infection rates revealed that the extended half-lives in the presence of SEVI or semen are independent of infectivity enhancing effects. Conclusively, semen increases HIV infection not only by promoting attachment of virions to target cells but also by increasing the infectious half-life. Consequently, antagonizing semen amyloid might not only lower the infectivity but also the stability of HIV-1 particles in semen, and might find application in microbicides designed to impede HIV spread.

#### O16 CD169^+^ macrophages mediate retrovirus ***trans***-infection of permissive lymphocytes to establish infection in vivo

##### Xaver Sewald^1,2^, Pradeep Uchil^2^, Mark Ladinsky^3^, Jagadish Beloor^4^, Ruoxi Pi^2^, Christin Herrmann^2^, Nasim Motamedi^1,2^, Thomas Murooka^5^, Michael Brehm^6^, Dale Greiner^6^, Thorsten Mempel^5^, Pamela Bjorkman^3^, Priti Kumar^4^, Walther Mothes^2^

###### ^1^Max von Pettenkofer Institute, LMU Munich, Dept. of Virology, Munich, Germany; ^2^Yale University, School of Medicine, Dept. of Microbial Pathogenesis, New Haven, CT, United States; ^3^California Institute of Technology, Division of Biology and Biological Engineering, Pasadena, CA, United States; ^4^Yale University, School of Medicine, Department of Medicine, New Haven, CT, United States; ^5^Harvard Medical School, Center for Immunology and Inflammatory Diseases, Boston, MA, United States; ^6^University of Massachusetts Medical School, Program in Molecular Medicine, Worcester, MA, United States

**Correspondence:** Xaver Sewald

*Retrovirology* 2016, **13(Suppl 1)**: O16

**Background:** Retroviruses can infect an organism at mucosal surfaces after sexual and mother to child transmission. They can also be transmitted horizontally through contact with body fluids such as blood, semen and saliva of retrovirus-infected individuals. Irrespective of the path retroviruses take to establish an infection within the host, retroviruses pass through secondary lymphoid organs such as lymph nodes and the spleen. Unfortunately, the critical events and the mechanism leading to the initial infection and subsequent spread of retroviruses at secondary lymphoid organs are currently unknown.

**Results:** Here, we show that sinus-lining macrophages of secondary lymphoid tissue contribute to establish infection by lymph- and blood-derived human immunodeficiency virus and murine leukemia virus. We identify the I-type lectin CD169/Siglec-1 to mediate Env-independent capture of retroviral particles followed by efficient trans-infection of permissive lymphocytes in peripheral lymph nodes and spleen. Using blocking antibodies and mice lacking CD169, we demonstrate that CD169-dependent trans-infection is required for the establishment of viral infection in mice. Applying intravital microscopy and EM tomography we visualize the formation of infectious synapses between CD169+ macrophages and target cells and subsequent virus transmission across cell–cell contacts in vivo.

**Conclusions:** Our results highlight the central role of CD169-expressing macrophages in retrovirus transmission and identify trans-infection as an important mechanism to establish retrovirus infection in vivo. Given the strategic position of CD169+ macrophages at the interface between extracellular body fluids such as lymph and blood to lymphoid tissues, our data suggest that retroviruses spread in vivo by a combination of cell-free spread within extracellular fluid followed by the CD169-dependent capture of cell-free virus and efficient trans-infection of permissive lymphocytes.

**Reference**

1. Sewald X*, Ladinsky M, Uchil P, Beloor J, Pi R, Herrmann C, Motamedi N, Murooka T, Brehm M, Greiner D, Shultz L, Mempel T, Bjorkman P, Kumar P*, Walther Mothes*. Retroviruses use CD169-mediated trans-infection of permissive lymphocytes to establish infection. Science. 2015;350(6260):563–7 (*corresponding author).

#### O17 Efficient replication of a ***vpu*** containing SIVagm construct in African Green Monkeys requires an HIV-1 *nef* gene

##### Simone Joas^1^, Erica Parrish^2^, Clement Wesley Gnanadurai^1,3^, Edina Lump^1^, Christina M. Stürzel^1^, Nicholas F. Parrish^2^, Ulrike Sauermann^4^, Katharina Töpfer^4^, Tina Schultheiss^4^, Steven Bosinger^5^, Guido Silvestri^5^, Cristian Apetrei^6^, Nicholas Huot^7^, Michaela Müller-Trutwin^7^, Daniel Sauter^1^, Beatrice H. Hahn^2^, Christiane Stahl-Hennig^4^, Frank Kirchhoff^1^

###### ^1^Ulm University Medical Center, Institute of Molecular Virology, Ulm, Germany; ^2^University of Pennsylvania, Departments of Medicine and Microbiology, Philadelphia, PA, United States; ^3^University of Georgia, Department of Pathology, Athens, GA, United States; ^4^German Primate Centre, Göttingen, Germany; ^5^Emory University, Emory Vaccine Center and Yerkes National Primate Research Center, Atlanta, GA, United States; ^6^University of Pittsburgh, Center for Vaccine Research, Pittsburgh, PA, United States; ^7^Institut Pasteur, Unité de Régulation des Infections Rétrovirales, Paris, France

**Correspondence:** Simone Joas

*Retrovirology* 2016, **13(Suppl 1)**: O17

The presence of a *vpu* gene and the lack of Nef-mediated downmodulation of the T cell receptor (TCR) CD3 distinguish HIV-1 and its simian precursors from primate lentiviruses that replicate efficiently in their natural host without causing disease. For example, SIVagm from African Green Monkeys (AGMs), which does not encode *vpu* but downmodulates CD3, is generally not pathogenic in its natural host species. Here, we generated HIV-1-like derivatives of SIVagm containing a functional *vpu* and/or an HIV-1 *nef* allele. Although all chimeric viruses replicated with similar efficiencies in cell culture, their replication fitness in infected AGMs was strikingly different. While insertion of *vpu* alone prevented SIVagm infection in vivo, insertion of HIV-1 *nef* attenuated viral replication after acute infection. SIVagm containing both *vpu* and HIV-1 *nef* maintained high viral loads for more than 4 years without causing disease, whereas individual introduction of *vpu* disrupted and of HIV-1 *nef* impaired viral replicaton in AGMs. Thus, Vpu and Nef cooperate to allow efficient viral replication in vivo and host factors seem to play a key role in the non-pathogenic course of SIVagm infection in AGMs.

To study the genetic and functional evolution of Vpu and Nef in the AGMs infected with SIVagm or the chimeric variants thereof, the 3′ halves of the viral genomes were amplified by single-genome amplification (SGA). The great majority of the PCR fragments contained intact *vpu* and *nef* ORFs, and sequence alignments revealed that these accessory genes were highly conserved. All Vpus tested were active in downmodulating CD4, NTB-A and CD1d. Furthermore, all AGM-derived *vpu* alleles suppressed NF-kB activation, reduced AGM Tetherin cell surface expression and promoted infectious virus release. The *nef* alleles maintained the functional properties of the parental SIVagm and HIV-1 *nef* genes. Notably, none of the HIV-1 *nef* alleles became active in downmodulating CD3 or in counteracting AGM tetherin, irrespectively of the presence of a *vpu* gene.

#### O18 Reprogramming initiates mobilization of endogenous mutagenic LINE-1, ***Alu*** and SVA retrotransposons in human induced pluripotent stem cells with consequences for host gene expression

##### Gerald Schumann^1^, Sabine Jung-Klawitter^1^, Nina V. Fuchs^1^, Kyle R. Upton^2^, Martin Muñoz-Lopez^3^, Ruchi Shukla^2^, Jichang Wang^4^, Marta Garcia-Canadas^3^, Cesar Lopez-Ruiz^3^, Daniel J. Gerhardt^2^, Attila Sebe^1^, Ivana Grabundzija^4^, Patricia Gerdes^2^, Sylvia Merkert^5^, Andres Pulgarin^3^, Anja Bock^1^, Ulrike Held^1^, Anett Witthuhn^5^, Alexandra Haase^5^, Ernst J. Wolvetang^6^, Ulrich Martin^5^, Zoltán Ivics^1^, Zsuzsanna Izsvák^4^, J. Garcia-Perez^3^, Geoffrey J. Faulkner^2^

###### ^1^Paul-Ehrlich-Institut, Medical Biotechnology, Langen, Germany; ^2^Mater Research Institute, Brisbane, Australia; ^3^GENYO, Granada, Germany; ^4^Max-Delbrück-Center for Molecular Medicine, Berlin, Germany; ^5^Hanover Medical School, Hanover, Germany; ^6^Australian Institute for Bioengineering and Nanotechnology, Brisbane, Australia

**Correspondence:** Gerald Schumann

*Retrovirology* 2016, **13(Suppl 1)**: O18

Human induced pluripotent stem cells (hiPSCs) can differentiate into every cell type of the adult body and hold substantial promise for regenerative medicine and as in vitro models of disease and development. However, reprogramming and subsequent cultivation of hiPSCs can induce genetic and epigenetic abnormalities that can result in tumorigenic hiPSCs. Thus, it is unclear if hiPSCs or their derivatives are safe for administration. Genomic mutations may undermine their use in regenerative medicine. Activation of the human endogenous mobile retrotransposons LINE-1 (Long Interspersed Element-1, L1), *Alu* and SVA has the potential to cause such mutations. In differentiated cells, L1 is primarily suppressed by methylation of its CpG-rich promoter, but we show that reprogramming triggers transcription of functional L1 elements via demethylation and specific transcription factors that are absent from differentiated cells. To investigate if the observed L1 activation in hiPSCs leads to L1-mediated mobilization, we applied retrotransposon capture-sequencing (RC-seq) to 8 hiPSC lines, their parental cells and human embryonic stem cell lines (hESCs). We identified, mapped and validated individual L1, *Alu* and SVA *de novo* retrotransposition events that occurred during reprogramming into hiPSCs and cultivation of hiPSCs and hESCs, and timed the period during hiPSC cultivation when individual *de novo* insertions occurred. Each hiPSC was estimated to carry ~1 L1 *de novo* insertion. As ~50 % of all *de novo* retrotransposition events occurred in protein-coding genes that are actively transcribed in hiPSCs, including genes playing roles in oncogenesis, development or signal transduction, we investigated effects of these intronic insertions on host gene expression in hiPSCs. To exemplify the consequences of even short intronic L1 *de novo* insertions, we analysed effects of the 390-bp L1 *de novo* insertion L1-dn13 in CADPS2 intron 7 on CADPS2 transcription in hiPSCs, and demonstrate significant interference of L1-dn13 with allelic CADPS2 gene expression. Our experiments demonstrate incidence, and functional impact of reprogramming-activated endogenous retrotransposition in hiPSCs and imply consequences for the biological safety of hiPSC-derived cell therapies.

#### O19 NF-κB activation induces expression of human endogenous retrovirus and particle production

##### Tara Hurst, Aris Katzourakis, Gkikas Magiorkinis

###### ^1^University of Oxford, Zoology, Oxford, Great Britain

**Correspondence:** Tara Hurst

*Retrovirology* 2016, **13(Suppl 1)**: O19

**Question:** Human endogenous retroviruses (HERVs) are prevalent in the human genome, mainly as defective proviruses or solo long terminal repeats (LTRs). The presence of HERVs can potentially alter human gene expression since the LTRs contain binding sites for numerous transcription factors, as well as for steroid hormone and nuclear receptors. While these sites have been predicted by sequence analysis, they have not been shown to be functional in vivo.

**Methods:** In order to assess the responsiveness of these binding sites, we used a cell line that is known to be permissive for HERV expression. The human embryonic carcinoma cell line, NCCIT, has been demonstrated to express HERVs and to produce mature particles. This is likely facilitated by the loss of the usual epigenetic suppression of HERV expression, such as by global hypomethylation that is frequently observed in cancer cells. The cells were treated with drugs, hormones and cytokines, then HERV-K *env* and *pol* expression was analysed by quantitative PCR (qPCR).

**Results:** We found that the cytokines interleukin-1α (IL-1α) and tumour necrosis factor α (TNFα), as well as the Toll-like receptor-4 (TLR4) ligand lipopolysaccharide (LPS), increased HERV transcription in the NCCITs. Further, these ligands dramatically increased HERV-K particle production by these cells.

**Conclusions:** Since these ligands activate innate immune signalling pathways to the transcription factor nuclear factor-κB (NF-κB), we hypothesise that the HERV-K LTRs are responsive to NF-κB binding. Thus, pro-inflammatory conditions could lead to increased HERV-K expression if the LTRs are de-repressed for other reasons, such as in the context of cancer.

### Session 7a and b: Innate sensing & intrinsic immunity

#### O20 Identification of the phosphatase acting on T592 in SAMHD1 during M/G_1_ transition

##### Kerstin Schott^1^, Rita Derua^2^, Janna Seifried^1^, Andreas Reuter^3^, Heike Schmitz^1^, Christiane Tondera^1^, Alberto Brandariz-Nuñez^4^, Felipe Diaz-Griffero^4^, Veerle Janssens^2^, Renate König^1,5^

###### ^1^Paul-Ehrlich-Institute, Host-Pathogen Interactions, Langen, Germany; ^2^KU Leuven, Department of Cellular and Molecular Medicine, Laboratory of Protein Phosphorylation and Proteomics, Leuven, Belgium; ^3^Paul-Ehrlich-Institute, Division of Allergology, Langen, Germany; ^4^Albert Einstein College of Medicine, Department of Microbiology and Immunology, New York City, NY, United States; ^5^Sanford Burnham Prebys Medical Discovery Institute, Immunity and Pathogenesis Program, La Jolla, CA, Germany

**Correspondence:** Kerstin Schott

*Retrovirology* 2016, **13(Suppl 1)**: O20

SAMHD1 is a critical HIV-1 restriction factor in non-dividing, myeloid and resting CD4^+^ T cells. As a dNTPase, SAMHD1 reduces dNTP levels below those required for reverse transcription. The restrictive activity of SAMHD1 is negatively regulated by phosphorylation: in cycling cells, SAMHD1 is phosphorylated at T592 and unable to restrict HIV-1 infection. Upon entry into a non-cycling state, T592 phosphorylation is lost and SAMHD1 rendered active against HIV-1.

SAMHD1 is phosphorylated by CDKs/cyclin A2, but the phosphatase acting on SAMHD1 is currently unknown. Using tandem affinity purification followed by MS analysis, we identified the Aα subunit of protein phosphatase 2A (PP2A) to potentially interact with SAMHD1. PP2A holoenzymes are composed of a scaffolding (A), catalytic (C) and variable regulatory (B) subunit, which can be recruited out of 4 different families. Specific PP2A holoenzymes are known to reverse CDK1-mediated phosphorylation events at the end of mitosis—consistent with the fact that SAMHD1 loses T592 phosphorylation rapidly upon entry into G_1_, as we could observe in synchronized HeLa ‘Kyoto’ cells using a thymidine block/release protocol.

After pull-down of PP2A-B55α trimers, SAMHD1 was specifically enriched. In line with this observation, only PP2A-B55α trimers were able to efficiently remove SAMHD1 phosphorylation at T592 in in vitro-dephosphorylation assays. We verified the specificity as (i) the inhibitor okadaic acid prevented dephosphorylation by PP2A and (ii) the protein phosphatase 1 (PP1) had no influence. Upon incubation with equilibrated units of *de novo* purified PP2A dimers or PP1, MS-based quantification of (non-)phosphorylated SAMHD1 peptides revealed that only PP2A dimers were able to remove phosphorylation at T592 in vitro. Furthermore, silencing of PP2A-B55α trimers in vivo using specific siRNAs led to increased T592 phosphorylation in HeLa ‘Kyoto’ cells. To characterise dephosphorylation at T592 at M/G_1_ transition in more detail, we chemically induced exit from mitosis in HeLa ‘Kyoto’ cells and observed impaired T592 dephosphorylation upon silencing of PP2A-B55α trimers. Taken together, we determined PP2A-B55α holoenzymes responsible for dephosphorylating SAMHD1 during M/G_1_ transition and rendering SAMHD1 active against HIV-1.

#### O21 Vpx overcomes a SAMHD1-independent block to HIV reverse transcription that is specific to resting CD4 T cells

##### Hanna-Mari Baldauf^1,2^, Lena Stegmann^2^, Sarah-Marie Schwarz^2^, Maud Trotard^3^, Margarethe Martin^2^, Gina Lenzi^4^, Manja Burggraf^5^, Xiaoyu Pan^3^, Oliver I. Fregoso^6^, Efrem S. Lim^6^, Libin Abraham^3^, Elina Erikson^2^, Laura Nguyen^4^, Ina Ambiel^2,3^, Frank Rutsch^7^, Renate König^5^, Baek Kim^4^, Michael Emerman^6^, Oliver T. Fackler^3^, Oliver T. Keppler^1,2^

###### ^1^Max von Pettenkofer Institute, Virology, Munich, Germany; ^2^Institute for Medical Virology, Frankfurt a. M., Germany; ^3^Department of Infectious Diseases, Integrative Virology, Heidelberg, Germany; ^4^Center for Drug Discovery, Department of Pediatrics, Atlanta, GA, United States; ^5^Paul-Ehrlich-Institute, Host-Pathogen-Interactions, Langen, Germany; ^6^Fred Hutchinson Cancer Research Center, Seattle, WA, United States; ^7^Department of General Pediatrics, Münster, Germany

**Correspondence:** Hanna-Mari Baldauf

*Retrovirology* 2016, **13(Suppl 1)**: O21

In contrast to activated CD4 T cells, resting CD4 T cells from peripheral blood are highly resistant to productive HIV-1 infection. Infection of resting CD4 T cells is restricted at the level of reverse transcription. This block can be overcome by virion-packaged Vpx proteins that target SAMHD1 for proteasomal degradation and elevate cellular dNTP pools. Here we find that Vpx proteins from distinct SIV lineages, SIV_rcm_ and SIV_mnd-2_, enhanced the HIV-1 susceptibility in the absence of SAMHD1 degradation or elevation of dNTP pools in resting CD4 T cells, but not macrophages. This infection enhancement was paralleled by a dramatic increase of RT intermediates and total HIV-1 cDNA. Single amino acid changes enabled the prototypic SAMHD1-degrading Vpx_mac239_ to enhance early post-entry step of HIV infection in rcm/mnd2-like fashion. Importantly, Vpx enhanced HIV infection of resting PMBCs of an AGS patient that lacks expression of functional SAMHD1. These results demonstrate that Vpx, in addition to SAMHD1, overcomes a previously unappreciated post-entry restriction of HIV-1 that is specific to resting CD4 T cells.

#### O22 The role of SAMHD1 in antiviral restriction and immune sensing in the mouse

##### Sabine Wittmann^1^, Rayk Behrendt^2^, Bianca Volkmann^1^, Kristin Eissmann^1^, Thomas Gramberg^1^

###### ^1^Universitätsklinikum Erlangen, Institute of Virology, Erlangen, Germany; ^2^Technical University Dresden, Institute for Immunology, Dresden, Germany

**Correspondence:** Thomas Gramberg

*Retrovirology* 2016, **13(Suppl 1)**: O22

SAMHD1 acts as a dNTP triphosphohydrolase and blocks the replication of retroviruses and retroelements in nondividing cells. Here, we use SAMHD1 knockout mice to analyze the regulation and the mechanism of SAMHD1 restriction and to determine the impact of SAMHD1 on antiviral sensing. Previously, we found that the lack of SAMHD1 triggers a spontaneous upregulation of IFN-inducible genes (ISGs) in various murine cell types. We therefore infected SAMHD1 knockout mice lacking the type I IFN receptor (IFNAR) with HIV reporter virus and analysed splenocytes of the mice 72 h postinfection. We found a strong increase in infected splenocytes of SAMHD1/IFNAR KO mice compared to SAMHD1 KO mice, suggesting an IFN-depended antiviral mechanism in the absence of SAMHD1 in vivo. In addition, we found that, similar to human SAMHD1, the antiviral activity of mouse SAMHD1 is regulated through phosphorylation and is limited to nondividing cells. Comparing the susceptibility to infection with intracellular dNTP levels and SAMHD1 phosphorylation showed that both functions are important determinants of the antiviral activity of murine SAMHD1. In contrast, we found the proposed RNase activity of SAMHD1 to be less important and could not detect any effect of mouse or human SAMHD1 on the level of incoming viral RNA. Our findings show that murine SAMHD1 blocks retroviral infection at the level of reverse transcription and is regulated through a cell cycle-dependent phosphorylation at threonine 603. Together, we show that the antiviral block mediated by murine SAMHD1 is mechanistically similar to what is known for the human protein, making the SAMHD1 KO mouse model a valuable tool to study the impact of SAMHD1 on the replication of different viruses in vivo.

#### O23 T cells expressing reduced restriction factors are preferentially infected in therapy naïve HIV-1 patients

##### Sebastian Bolduan^1^, Herwig Koppensteiner^1^, Stefanie Regensburg^1^, Ruth Brack-Werner^1^, Rika Draenert^2^, Michael Schindler^1,3^

###### ^1^Helmholtz Center Munich, Institute of Virology, Neuherberg, Germany; ^2^Ludwig-Maximilians-University Munich, Munich, Germany; ^3^University Hospital Tuebingen, Institute of Medical Virology, Tuebingen, Germany

**Correspondence:** Michael Schindler

*Retrovirology* 2016, **13(Suppl 1)**: O23

**Question:** So-called host cell restriction factors (RFs) potently suppress HIV-1 replication in cell lines and primary cell culture models. In theory, RFs might represent attractive targets for the development of novel antiviral treatment strategies but their importance for virus control in vivo is controversial.

**Methods:** Here, we profiled the expression of RFs including p21, SAMHD1 and Tetherin in primary blood-derived mononuclear cells (PBMC) from untreated, therapy-naïve HIV-1 patients and quantified cellular infection levels.

**Results:** There was overall no correlation between the expression of the individual RFs and HIV-1 control versus progression in patients. However, we identified a T cell population that was negative for intracellular CD2 and expresses low levels of SAMHD1, p21 and the recently identified inhibitor of HIV-1 infectivity SerinC5. CD2-negative T-cells with low RF expression levels were highly HIV-infected in comparison to their CD2+ counterparts and the extent of CD2-negative T-cell infection was a marker of HIV-1 progression. Altogether, we report an association of RF expression levels with the extent of HIV-1 infection in primary T cells directly isolated from untreated patients.

**Conclusions:** Our study supports the in vivo importance of RFs for HIV-1 control and highlights RFs as promising targets for therapeutic intervention.

#### O24 cGAS-mediated innate immunity spreads through HIV-1 env-induced membrane fusion sites from infected to uninfected primary HIV-1 target cells

##### Aurélie Ducroux, Shuting Xu, Aparna Ponnurangam, Sergej Franz, Angelina Malassa, Ellen Ewald, Christine Goffinet

###### Twincore, Experimental Virology, Hanover, Germany

**Correspondence:** Christine Goffinet

*Retrovirology* 2016, **13(Suppl 1)**: O24

Upon HIV infection, reverse transcribed DNA present in the cytoplasm can be sensed by cellular pattern recognition receptors, resulting in the induction of antiviral type I IFN and expression of interferon-stimulated genes that exert numerous antiviral functions. Specifically, upon DNA binding, the protein cyclic GMP-AMP Synthase (cGAS) synthesizes the unique second messenger cGAMP that binds to STING to activate downstream IFN signaling. It has been proposed that this signaling pathway is engaged upon HIV infection, especially in the absence of TREX1, a cellular exonuclease that degrades excess viral DNA that escapes the viral core. However, HIV-1 perfectly inhibits and evades intrinsic immune responses. Most of these studies have been performed with cell-free HIV-1 particles.

We established an HIV-1 cell-to-cell transmission model using HIV-1-infected human PBLs and autologous macrophages as donor and target cell populations, respectively. Upon cell-to-cell transmission, HIV-1 infection led to a robust interferon response, an observation which was not recapitulated in assays using cell-free HIV-1 particles although the resulting percentage of infected target macrophages was in a similar range as in the condition of cell-to-cell transmission. Specifically, IFN-beta mRNA expression and release of bioactive type I IFN in the culture supernatant were induced.

To understand the specific signatures of the cellular responses to each infection mode, the expression of known cellular key players was decreased using siRNA in target macrophages showing that this IFN induction required STING, but not cGAS. Moreover, direct contact of donor and target cells and functional HIV-1 Env-mediated fusion were necessary. HIV-1-infected PBLs, but not their cell-free virions, contained SVPDE-sensitive, IFN-inducing small molecules, most conceivably cGAMP. These data are in line with intercellular transfer of cGAMP from infected PBLs to target macrophages, where cGAMP binds to STING and triggers a type I IFN induction in a fashion that bypasses the necessity of functional cGAS in macrophages.

We propose that, in HIV-1-infected tissues, cGAMP-mediated innate immunity spreads predominantly via direct cell–cell contacts that result in cytoplasmic mixing of HIV-1-infected and neighboring target cells.

#### O25 Perturbation of innate RNA and DNA sensing by human T cell leukemia virus type 1 oncoproteins

##### Sin-Yee Fung^1^, Ching-Ping Chan^1^, Chun-Kit Yuen^2^, Kin-Hang Kok^2^, Chin-Ping Chan^1^, Dong-Yan Jin^1^

###### ^1^The University of Hong Kong, School of Biomedical Sciences, Pokfulam, Hong Kong; ^2^The University of Hong Kong, Department of Microbiology, Pokfulam, Hong Kong

**Correspondence:** Dong-Yan Jin

*Retrovirology* 2016, **13(Suppl 1)**: O25

Human T-cell leukemia virus type 1 (HTLV-1) chronically infects 5–20 million people worldwide, causing adult T-cell leukemia (ATL) and tropical spastic paraparesis in a small subset of them. Interferon (IFN) α is a key innate immune effector and its combination with zidovudine, which is a nucleoside reverse transcriptase inhibitor, is the recommended standard first-line therapy for ATL. It is thought that viral RNA and proviral genome in HTLV-1-infected cells are sensed by the host sensor proteins to induce type I IFNs. Exactly how HTLV-1 perturbs this induction remains to be fully understood. In this study we report on the perturbation of innate RNA and DNA sensing by HTLV-1 oncoproteins Tax and HBZ. The induction of IFN-β production was blunted in HTLV-1-transformed ATL cells and in T lymphocytes freshly infected with HTLV-1. Interestingly, HTLV-1 oncoproteins Tax and HBZ displayed differential activity to perturb innate RNA sensing mediated by RIG-I and PACT as well as innate DNA sensing mediated by cGAS, cGAMP and STING. The perturbation occurred at a step prior to IRF3 activation since neither Tax nor HBZ was capable of suppressing the activity of a dominant active phosphomimetic IRF3 mutant IRF3-5D. Tax and HBZ were found to associate with TBK1, IKKε, STING and IRF3. *In vitro* phosphorylation assay indicated the suppression of TBK1-mediated phosphorylation of IRF3 and other substrates by Tax. Taken together, our findings suggested that HTLV-1 oncoproteins Tax and HBZ differentially modulate innate RNA and DNA sensing in infected cells. Our findings might reveal new strategies and compounds that could be used to improve IFN-based anti-HTLV-1 and anti-ATL therapy. Supported by HKRGC (HKU171091/14M, HKU271215/15 and C7011-15R), HMRF (HKM-15-M01 and 15140682) and SK Yee Medical Research Fund (2011).

#### O26 Induction and anti-viral activity of Interferon α subtypes in HIV-1 infection

##### Ulf Dittmer

###### University Hospital Essen, Institute for Virology, Essen, Germany

**Correspondence:** Ulf Dittmer

*Retrovirology* 2016, **13(Suppl 1)**: O26

HIV-1 is transmitted primarily across mucosal surfaces and rapidly spreads within the intestinal mucosa during acute infection. The type I interferons (IFNs) likely serve as a first line of defense, but the relative expression and antiviral properties of the 12 IFNα subtypes against HIV-1 infection remain unknown. We evaluated the expression of all IFNα subtypes in HIV-1-exposed plasmacytoid dendritic cells and PBMC of HIV-infected individuals. We also determined the relative antiviral potency of each IFNα subtype ex vivo using the human intestinal Lamina Propria Aggregate Culture or an PBMC model. IFNα8, IFNα6, IFNα14, IFNα17, and IFNα21 were the most potent in restricting HIV-1 infection in vitro. IFNα2, the clinically-approved subtype, and IFNα1 were both highly expressed but exhibited relatively weak antiviral activity. The relative potencies correlated the induction levels of HIV-1 restriction factors Mx2 and Tetherin/BST-2.

We also demonstrate in a humanized mouse model that, when delivered at the same high clinical dose, the human IFNα14 subtype has very potent anti-HIV-1 activity in vivo whereas IFNα2 does not. In both post-exposure prophylaxis and treatment of acute infections, IFNα14 but not IFNα2 significantly suppressed HIV-1 replication and proviral loads. Whereas ineffective IFNα2 therapy was associated with CD8+ T cell activation, successful IFNα14 therapy was associated with increased intrinsic and innate immunity including significantly higher induction of tetherin and MX2, increased APOBEC3G signature mutations in HIV-1 proviral DNA, and higher frequencies of TRAIL + NK cells. These results identify IFNα14 as a potent new therapeutic that operates via mechanisms distinct from antiretroviral drugs. The ability of IFNα14 to reduce both viremia and proviral loads in vivo suggests that it has strong potential as a component of a cure strategy for HIV-1 infections.

#### O27 Vpu-mediated counteraction of tetherin is a major determinant of HIV-1 interferon resistance

##### Dorota Kmiec^1^, Shilpa Iyer^2^, Christina Stürzel^1^, Daniel Sauter^1^, Beatrice Hahn^2^, Frank Kirchhoff^1^

###### ^1^Ulm University, Ulm, Germany; ^2^University of Pennsylvania, Philadelphia, PA; United States

**Correspondence:** Dorota Kmiec

*Retrovirology* 2016, **13(Suppl 1)**: O27

HIV-1 groups M, N, O and P are the result of independent zoonotic transmissions of SIVs infecting great apes in Africa. Among these, only Vpu proteins of pandemic HIV-1 group M strains evolved potent activity against the restriction factor tetherin which inhibits virus release from infected cells. Thus, effective Vpu-mediated tetherin antagonism may have been a prerequisite for the global spread of HIV-1. To determine whether this particular function enhances primary HIV-1 replication and interferon resistance, we introduced mutations into the *vpu* gene of HIV-1 group M and N strains to specifically disrupt their ability to antagonize tetherin, but not other Vpu functions, such as degradation of CD4, down-modulation of CD1d and NTB-A, and suppression of NF-κB activity. Lack of particular human-specific adaptations reduced the ability of HIV-1 group M Vpu proteins to enhance virus production and release from primary CD4+ T cells at high levels of type I IFN from about fivefold to twofold. Interestingly, transmitted-founder HIV-1 strains exhibited higher virion release capacity than chronic control HIV-1 strains irrespective of Vpu function, and group M viruses produced higher levels of cell-free virions than an N group HIV-1 strain. Thus, efficient virus release from infected cells seems to play an important role in the spread of HIV-1 in the human population and requires a fully functional Vpu protein that counteracts human tetherin.

#### O28 DNA repair protein Rad18 restricts HIV-1 and LINE-1 life cycle

##### Yasuo Ariumi^1^, Mariko Yasuda-Inoue^1^, Koudai Kawano^1^, Satoshi Tateishi^2^, Priscilla Turelli^3^

###### ^1^Kumamoto University, Center for AIDS Research, Kumamoto, Japan; ^2^Kumamoto University, Institute of Molecular Embryology and Genetics, Kumamoto, Japan; ^3^EPFL, Lausanne, Switzerland

**Correspondence:** Yasuo Ariumi

*Retrovirology* 2016, **13(Suppl 1)**: O28

**Introduction:** Long interspersed element type 1 (LINE-1, L1) is a mobile genetic element comprising about 17 % of the human genome. L1 utilizes an endonuclease to insert L1 cDNA into the target genomic DNA, which induces double-strand DNA breaks (DSBs). Likewise, human immunodeficiency virus-1 (HIV-1) integration induces DSBs in the human genome and activates DNA damage signaling pathway resulting in a recruitment of DNA repair protein(s). This may facilitate HIV-1 and L1 integration in the human genome. Therefore, host DNA repair machinery has been involved in both HIV-1 and L1 life cycle.

**Results:** In this study, we have demonstrated that Rad18 post-replication repair protein restricts both HIV-1 and L1 life cycle. Notably, HIV-1 infection or L1 retrotransposition efficiency was enhanced in the Rad18 deficient or the knockdown cells. In contrast, overexpression of Rad18 strongly suppressed L1 retrotransposition as well as L1-mediated Alu retrotransposition. The RING-finger and the Rad6 (E2 ubiquitin-conjugated enzyme)-binding domains but not the Polη-binding domain were required for the inhibitory effect on L1 retrotransposition, suggesting that the ubiquitin E3 ligase activity of Rad18 is important for regulation of L1 mobility. Furthermore, Rad18 sequestered L1 ORF1p in RAD18-nuclear bodies and bound with L1 ORF1p. Similarly, HIV-1 integrase colocalized with Rad18 in Rad18-nuclear bodies and bound with Rad18, indicating an interaction of Rad18 with HIV-1 integrase. Moreover, we found that Rad18 suppressed the late step of HIV-1 replication.

**Conclusion:** Altogether, these results suggest a potential role of Rad18 DNA repair protein in HIV-1 life cycle and L1 retrotransposition process.

#### O29 Natural mutations in ***IFITM3*** allow escape from post-translational regulation and toggle antiviral specificity

##### Alex Compton, Nicolas Roy, Françoise Porrot, Anne Billet, Nicoletta Casartelli, Jacob Yount, Chen Liang, Oliver Schwartz

###### Institut Pasteur, Paris, France

**Correspondence:** Alex Compton

*Retrovirology* 2016, **13(Suppl 1)**: O29

The interferon-induced transmembrane (IFITM) proteins protect host cells from diverse virus infections. IFITM also incorporate into HIV-1 virions and inhibit virus fusion and cell-to-cell spread, with IFITM3 showing the greatest potency. Here we report that amino-terminal mutants of IFITM3 preventing ubiquitination and endocytosis are more abundantly incorporated into virions and exhibit enhanced inhibition of HIV-1 fusion. An analysis of primate genomes revealed that *IFITM3* is the most ancient antiviral family member of the *IFITM* locus and has undergone repeated duplication in independent host lineages. Some *IFITM3* genes in non-human primates, including those that arose following gene duplication, carry amino-terminal mutations that modify protein localization and function. This suggests that “runaway“IFITM3 variants could be selected for altered antiviral activity. Furthermore, we show that adaptations in *IFITM3* result in a trade-off in antiviral specificity, as variants exhibiting enhanced activity against HIV-1 poorly restrict Influenza A virus. Overall, we provide the first functional evidence that variation in *IFITM3* genes may boost the antiviral coverage of host cells and provide selective functional advantages.

### Session 8: Adaptive immunity & immune evasion

#### O30 Observing evolution in HIV-1 infection: phylogenetics and mutant selection windows to infer the influence of the autologous antibody response on the viral quasispecies

##### Carsten Magnus^1^, Lucia Reh^2^, Penny Moore^3^, Therese Uhr^2^, Jacqueline Weber^2^, Lynn Morris^3^, Alexandra Trkola^2^

###### ^1^ETH Zurich, D-BSSE, Computational Evolution, Basel, Switzerland; ^2^University of Zurich, Instiute of Medical Virology, Zurich, Switzerland; ^3^University of the Witwatersrand, Faculty of Health Sciences, Johannesburg, South Africa

**Correspondence:** Carsten Magnus

*Retrovirology* 2016, **13(Suppl 1)**: O30

**Question:** During HIV-1 infection, a constant interplay of antibodies and the autologous viral quasi-species shapes the developing antibody response and potentially leads to viral escape. A better understanding of these co-evolutionary processes is urgently needed both for designing a vaccine scheme eliciting an effective antibody response and for preventing viral escape in passive immunisation with broadly neutralising antibodies (bnAbs). Of particular importance is the right dosage of these bnAbs as too low concentrations may lead to fast escape of the virus. Thus, we study here at which antibody concentrations a viral escape variant can outcompete its viral ancestor, referred to as mutant selection window (MSW).

**Methods:** To determine the MSWs of an ancestor/escape pair in respect to a specific bnAb, we analysed experimentally derived inhibition measures (IC50) with a mathematical model. As within-host viral transmission can happen via free-virus transmission and through direct spread between infected and target cells (cell–cell transmission), we calculated the MSW for both transmission routes. With the MSW framework, we characterised the selective pressure of a bnAb lineage leading to viral escape in the HIV-infected individual CAP256 using in vivo-derived longitudinally sampled virus strains and isolated autologous bnAbs. We identified potential CAP256 ancestor/escape pairs based on the viral phylogeny.

**Results:** We found that escape mutants can out-compete their sensitive ancestral strains for wide concentration ranges in both transmission pathways. The MSW of these viral pairs in respect to the autologous bnAbs additionally allowed us to identify (i) which autologous virus strain will be out-competed (ii) which strain could be a possible ancestor of an escape variant and (iii) whether escape predominantly occurs via free virus or cell–cell transmission.

**Conclusions:** Our method provides important implications for antibody-based treatment strategies and vaccine design. Not only will the MSW framework allow the selection of the appropriate antibody dosage to suppress the development of escape mutants but, in addition, may help to design a vaccine scheme that mimics the co-evolutionary processes of a natural HIV-1 infection leading to a protective broadly neutralising antibody response in uninfected individuals.

#### O31 Dose and subtype specific analyses of the anti-HIV effects of IFN-alpha family members

##### Rashel V. Grindberg^1^, Erika Schlaepfer^1^, Gideon Schreiber^2^, Viviana Simon^3^, Roberto F. Speck^1^

###### ^1^University Hospital Zurich, Infectious Disease and Hospital Epidemiology, Zurich, Switzerland; ^2^Weizmann Institute of Science, Department of Biological Chemistry, Rehovot, Israel; ^3^Icahn School of Medicine at Mount Sinai, Department of Microbiology, New York City, NY, United States

**Correspondence:** Rashel V. Grindberg

*Retrovirology* 2016, **13(Suppl 1)**: O31

Interferons (IFN) are cytokines that are fundamental to innate and adaptive immune responses and are named so by their ability to “interfere” with viral replication. The family of human IFN-alpha’s (IFN-α) is encoded on chromosome 9 and comprises 13 different subtypes [1]. These molecules signal through the IFN-α receptors 1 and 2, prompting Jak/Stat activation and induction of interferon stimulated genes (ISGs) [2]. IFN-α 1 and 2 were the first two alpha subtypes characterised [3]. IFN-α2 has a higher specificity and activity than IFN-α1 and so became the prototype IFN for most subsequent studies. Data reporting the anti-viral effects of the other subtypes is sparse.

Here we analysed 12 IFN family members and 6 mutant IFN variants, engineered to have various binding efficiencies, for their ability to inhibit HIV-1 replication in primary human cells such as peripheral blood mononuclear cells, purified CD4+ T-cells as well as monocyte derived macrophages. We tested a range of concentrations (10 U/ml, 100 U/ml and 1000 U/ml) for each of the IFNs. We found a significant difference in activities of the natural IFN variants at lower dosages, which was lost at higher concentrations. Similarly, at lower dosages, the IFN mutants with stronger (60×) binding affinity showed a higher inhibition than the mutants with lower (40×) affinity. However, this difference was also reduced with increasing concentrations, indicating that differential antiviral efficacies between IFN subtypes can be compensated for with higher amounts. These results are consistent with RNA-seq, differential expression and biological network analyses of IFNα-2, IFNα-14 and IFN mutant stimulated macrophages at high and low doses.

Together, these observations bring into focus the importance of understanding putative differential IFN antiviral activity as a function of binding affinity, potency and dosage.

**References**

1. Gibbert K, et al. Br J Pharmacol. 2013;168(5):1048–58.
2. Hyrcza MD, et al. J Virol. 2007;81(7):3477–86.
3. Paul F, Pellegrini S, Uze G. Gene. 2015;567(2):132–7.

### Session 9: Novel antiviral strategies

#### O32 LEDGIN-mediated inhibition of the integrase-LEDGF/p75 interaction reduces reactivation of residual latent HIV

##### Zeger Debyser^1^, Lenard Vranckx^1^, Jonas Demeulemeester^1^, Suha Saleh^1^, Eric Verdin^2^, Anna Cereseto^2,3^, Frauke Christ^1^, Rik Gijsbers^1^

###### ^1^KU Leuven, Leuven, Belgium; ^2^Gladstone Institute of Virology and Immunology, University of California, San Francisco, CA, United States; ^3^Centre for Integrative Biology (CIBIO), Trento, Italy

**Correspondence:** Zeger Debyser

*Retrovirology* 2016, **13(Suppl 1)**: O32

Persistence of latent, replication-competent HIV provirus is the main impediment towards a cure for HIV/AIDS. Therefore, different therapeutic strategies to eliminate this latent reservoir are currently being explored. Persistence is in part a consequence of proviral integration. LEDGF/p75 acts as the pivotal chromatin tethering-factor targeting HIV integration into active transcription units through its interaction with HIV-1 integrase. We investigated the role of integration site selection in the establishment of HIV persistence employing LEDGF/p75 knockout cells. To evaluate whether the reactivation potential of the quiescent proviral reservoir was dependent on integration site selection during reservoir establishment, LEDGF/p75 WT and KD/KO cell lines were infected with HIV_NL4.3-tCD34_ or a double reporter virus that allowed direct visualization of the quiescent pool by FACS analysis. We show for the first time that LEDGF/p75 depletion hampers HIV-1 reactivation in cell culture.

Next we demonstrate that LEDGINs, a novel class of integration inhibitors inhibiting the interaction between HIV integrase and the LEDGF/p75 host cofactor, relocate 3D nuclear location and retarget HIV proviral integration out of transcription units, resulting in a HIV reservoir that is refractory to reactivation by different latency-reversing agents both in cell lines an primary CD4+ T-cells. We here propose a novel strategy to reduce the functional HIV reservoir during primary HIV infection by means of drug-induced retargeting of HIV integration and support the potential of these drugs to reduce the likeliness of viral rebound. Pushing the provirus into quiescence could drive the basic reproduction number of HIV below a threshold required for sustained infection even after treatment interruption.

#### O33 NKG2D-mediated clearance of reactivated viral reservoirs by natural killer cells

Permission for the publication has not been granted.

#### O34 Inhibition of HIV reactivation in brain cells by AAV-mediated delivery of CRISPR/Cas9

Permission for the publication has not been granted.

#### O35 CRISPR-Cas9 as antiviral: potent HIV-1 inhibition, but rapid virus escape and the subsequent design of escape-proof antiviral strategies

##### Ben Berkhout, Gang Wang, Na Zhao , Atze T. Das

###### Academic Medical Center of the University of Amsterdam, Laboratory of Experimental Virology, Amsterdam, Netherlands

**Correspondence:** Ben Berkhout

*Retrovirology* 2016, **13(Suppl 1)**: O35

Several recent studies demonstrated that the CRISPR-associated endonuclease Cas9 can be used for guide RNA (gRNA)-directed, sequence-specific cleavage of HIV proviral DNA in infected cells. We here demonstrate profound inhibition of HIV-1 replication by harnessing T cells with Cas9 and an antiviral gRNA. However, the virus rapidly and consistently escaped from this inhibition. Sequencing of the HIV-1 escape variants revealed nucleotide insertions, deletions and substitutions around the Cas9/gRNA cleavage site that are typical for DNA repair by the non-homologous end-joining (NHEJ) pathway. We thus demonstrate the potency of CRISPR-Cas9 as an antiviral approach, but any therapeutic strategy should consider these viral escape options. We will present several combinatorial therapeutic approaches designed to block virus escape. More specifically, we tested the simultaneous attack by different guide RNAs on HIV-1 DNA, but also the combinatorial attack on both HIV-1 RNA and DNA forms by combining RNAi and CRISPR-Cas approaches.

### Session 10: Recent advances in HIV vaccine development

#### O36 Priming with a potent HIV-1 DNA vaccine frames the quality of T cell and antibody responses prior to a poxvirus and protein boost

##### Benedikt Asbach^1^, Josef Köstler^2^, Beatriz Perdiguero^3^, Mariano Esteban^3^, Bertram L. Jacobs^4^, David C. Montefiori^5^, Celia C. LaBranche^5^, Nicole L. Yates^5^, Georgia D. Tomaras^5^, Guido Ferrari^5^, Kathryn E. Foulds^6^, Mario Roederer^6^, Gary Landucci^7^, Donald N. Forthal^7^, Michael S. Seaman^8^, Natalie Hawkins^9^, Steven G. Self^9^, Sanjay Phogat^10^, James Tartaglia^10^, Susan W. Barnett^11^, Brian Burke^11^, Anthony D. Cristillo^12^, Song Ding^13^, Jonathan L. Heeney^14^, Giuseppe Pantaleo^15^, Ralf Wagner^1^

###### ^1^University Regensburg, Institute for Medical Microbiology and Hygiene, Regensburg, Germany; ^2^University Regensburg, Institute for Clinical Microbiology and Hygiene, Regensburg, Germany; ^3^Centro Nacional de Biotecnología, Madrid, Spain; ^4^Arizona State University, Biodesign Institute, Tempe, AZ, United States; ^5^Duke University Medical Center, Durham, NC, United States; ^6^National Institutes of Health, Vaccine Research Center, Bethesda, MD, United States; ^7^University of California, Irvine, CA, United States; ^8^Beth Israel Deaconess Medical Center, Center for Virology and Vaccine Research, Boston, MA, United States; ^9^Fred Hutchinson Cancer Research Center, Statistical Center for HIV/AIDS Research and Prevention, Seattle, WA, United States; ^10^Sanofi Pasteur, Swiftwater, PA, United States; ^11^Novartis Vaccines and Diagnostics, Inc., Cambridge, MA, United States; ^12^Advanced BioScience Laboratories, Inc., Rockville, MD, United States; ^13^EuroVacc Foundation, Lausanne, Switzerland; ^14^University of Cambridge, Department of Veterinary Medicine, Cambridge, Great Britain; ^15^University of Lausanne, Centre Hospitalier Universitaire Vaudois, Lausanne, Switzerland

**Correspondence:** Benedikt Asbach

*Retrovirology* 2016, **13(Suppl 1)**: O36

The use of heterologous immunisation regimens, and the employment of various improved vector systems as well as antigen designs, has proven to lead to vigorous increases in immunogenicity in the assessment of HIV-1 vaccine candidates in non-human primates. In order to resolve interdepencies between different delivery modalities, we compared three different poxvirus boost regimens after a DNA prime. Three groups of rhesus macaques were each immunized with the same DNA vaccine encoding for Gag, PolNef, and gp140 at weeks 0, 4 and 8. At week 20, the groups were boosted either (i) by administering the poxviral replication-competent NYVAC-KC-vector by scarification, or (ii) by i.m. injection, or (iii) by i.m. injection of the replication-deficient NYVAC-vector, carrying the same antigens. Finally, macaques were boosted with adjuvanted, recombinant gp120 protein at weeks 28, 32, and 49 in order to enhance humoral responses. The regimen elicited very potent CD4+ and CD8+ T cell responses in a well-balanced manner, peaking 2 weeks after the NYVAC-boost. T cell responses subsequently declined and were hardly influenced by subsequent protein boosts. T-cells were broadly reactive and polyfunctional, with high fractions of cells secreting all three cytokines assessed. All animals exhibited antigen-specific humoral responses already after the poxvirus boost, that by trend slightly increased following protein administration. Polyclonal reactivity of IgG antibodies was highest against C clade Env-proteins, yet with substantial cross-reactivity towards other clades. Serum IgA responses were absent. Substantial ADCC activity, and very high ADCVI activity were observed in sera obtained after the last protein boost.

As no differences were evident between the groups, it can be concluded that the potent priming induced by the DNA vaccine initially framed the epitope specificity and polyfunctionality of the T cell responses in a way that the subsequent poxvirus boost only led to an increase in the response magnitudes without skewing the quality. This emphasizes the importance of selecting the best mixture of vector systems in heterologous vaccination regimens.

#### O37 Passive immunisation with a neutralising antibody against HIV-1 Env prevents infection of the first cells in a mucosal challenge rhesus monkey model

##### Christiane Stahl-Hennig^1^, Viktoria Stab^2^, Armin Ensser^3^, Ulrike Sauermann^1^, Bettina Tippler^2^, Dennis Burton^4^, Matthias Tenbusch^2^, Klaus Überla^3^

###### ^1^German Primate Center, Infection Models, Göttingen, Germany; ^2^Ruhr-University Bochum, Department of Molecular and Medical Virology, Bochum, Germany; ^3^Friedrich-Alexander University Erlangen-Nuremberg, Institute of Clinical and Molecular Virology, Erlangen, Germany; ^4^The Scripps Research Institute, Department of Immunology and Microbial Science, La Jolla, CA, United States

**Correspondence:** Christiane Stahl-Hennig

*Retrovirology* 2016, **13(Suppl 1)**: O37

Pretreatment with antibodies targeting the HIV-1 Env protein has been shown to prevent systemic infection in non-human primate models for AIDS. To investigate whether these antibodies can block infection of the “first cell” at the viral portal of entry, genetically tagged challenge viruses based on simian immunodeficiency virus (SIV) were constructed. They use HIV-1 Env for entry into target cells during the first replication cycle, but switch to SIV Env for all subsequent rounds of infection. Macaques were passively immunized with the HIV-1 Env specific neutralising antibody PGT121 a day before rectal exposure to the switching challenge virus. Ten days after viral inoculation a 100-fold reduction of HIV-1 Env-mediated infection events was observed in various organs and blood compared to control animals treated with a HIV-unrelated antibody. A lesser level of inhibition of infection was also detected against a HIV Env pseudotype, which is resistant to PGT121 neutralisation. Thus, antiviral antibodies can reach sufficient levels at mucosal surfaces to provide sterilizing immunity in a very strict sense.

#### O38 HIV antibody Fc-glycoforms drive B cell affinity maturation

##### Galit Alter^1^, Giuseppe Lofano^2^, Anne-Sophie Dugast^1^, Viraj Kulkarni^3^, Todd Suscovich^1^

###### ^1^Ragon Institute of MGH, MIT and Harvard, Cambridge, MA, United States; ^2^Novartis Vaccines and Diagnostics S.r.l. (a GSK Company), Research Center, Siena, Italy; ^3^National Cancer Institute, Center for Cancer Research, Frederick, MD, United States

**Correspondence:** Galit Alter

*Retrovirology* 2016, **13(Suppl 1)**: O38

HIV broadly neutralising antibodies (bNAbs) confer protection following passive immunisation, but the mechanisms that allow these humoral immune responses to evolve in a fraction of infected individuals is unclear. Features of bNAbs suggest that extensive germinal center (GC) reactions are required to drive these unusual super-high affinity responses required to drive broad viral neutralisation. However, the mechanisms that underlie this extensive affinity maturation are poorly understood. Given that antigens are delivered to follicular dendritic cells (FDCs), the key antigen presenting cells in the GC, in the form of antibody immune complexes, here we speculated that key features of immune complexes may drive enhanced affinity maturation. Thus using a set of high-throughput, comprehensive Fc-characterizing assays that capture the remarkable biodiversity of antibody Fc-effector functions, linked to multivariate computational tools, we profiled differences in immune complex (IC) biology among individuals that develop neutralising antibodies (“neutralizers”) in an unbiased manner. “Neutralizers” possessed higher Fc-mediated antibody effector functions, HIV-specific antibody titers, and overall enhanced binding to Fc-receptors as compared to subjects that did not possess broadly neutralising antibodies. Interestingly, enhanced antibody effector function in “Neutalizers” was not associated with overall changes in antibody subclass distribution but was associated with the selective production of antibodies that were more highly sialylated. Interestingly, ICs generated with “neutralizer” antibodies drove enhanced antibody class switch and affinity maturation following immunisation of mice compared to ICs generated with “non-neutralizer” antibodies. Moreover, ICs generated with solely sialylated antibodies demonstrated en equally enhanced capacity to drive B cell maturation by class switch, to expand GC B cell numbers, and drive enhanced antibody affinity maturation. These data argue that the generation of particular antigen-specific sialylated Fc-profiles drives enhanced antibody maturation, potentially contributing the prolonged affinity maturation required for the evolution of broadly neutralising antibody responses. Thus rational vaccine design strategies that induce enhanced sialylated HIV-specific antibodies may enhance affinity maturation and therefore accelerate the induction of broadly neutralising antibodies against HIV.

## Poster presentations

### Topic 1: Entry & uncoating

#### P1 Dynein light chain is required for murine leukemia virus infection

##### Tatiana Opazo^1,2^, Felipe Barraza^1,2^, Diego Herrera^1,2^, Andrea Garces^1^, Tomas Schwenke^1,2^, Diego Tapia^1^, Jorge Cancino^1^, Gloria Arriagada^1,2^

###### ^1^Universidad Andres Bello, Ciencias Biologicas, Viña del Mar, Chile; ^2^Millenium Nucleus Biology of Neuropsiquiatric Disorders NuMIND, Valparaiso, Chile

**Correspondence:** Gloria Arriagada

*Retrovirology* 2016, **13(Suppl 1)**: P1

**Question:** How Murine Leukemia virus (MLV) travels from the cell membrane towards the nucleus, and the mechanism of nuclear entry of MLV viral DNA in dividing cells, still remain unclear. It seems likely that the MLV preintegration complex (PIC) interacts with cellular proteins to perform those tasks. We have recently published that microtubule motor cytoplasmic dynein complex (DC) and its regulator proteins interact with MLV preintegration complex at early stages of infection, suggesting a direct interaction between the incoming viral particles and the DC, and have shown an essential role for the dynein regulators dynactin and NudEL on MLV infection.

**Methods:** Here we show a shRNA screening of the dynein chains on MLV infection.

**Results:** We found that silencing or over expressing a specific light chain of the cytoplasmic DC profoundly reduced the efficiency of infection and increases the infection of MLV in a dose dependent manner, respectively. The block of restriction was determined to be in a step after reverse transcription, but before nuclear entry, by MLV reporter viruses, without altering traffic of cellular components.

**Conclusions:** We propose that the light chains of the cytoplasmic DC are an important piece of the host machinery needed for MLV infection.

#### P2 Peptide paratope mimics of the broadly neutralising HIV-1 antibody b12

##### Christina Haußner^1^, Dominik Damm^2^, Anette Rohrhofer^2^, Barbara Schmidt^2^, Jutta Eichler^1^

###### ^1^Friedrich-Alexander-University Erlangen-Nuremberg, Department of Chemistry and Pharmacy, Erlangen, Germany; ^2^Institute of Microbiology and Hygiene, Clinical Virology and Infection Immunology, Regensburg, Germany

**Correspondence:** Barbara Schmidt

*Retrovirology* 2016, **13(Suppl 1)**: P2

**Introduction:** Broadly neutralising antibodies (bnAbs) against envelope structures of HIV-1 are promising candidates for antiviral prophylaxis or therapy. We hypothesized that peptides mimicking the complementarity-determining regions (CDRs) of the bnAb b12, which recognizes the CD4 binding site of the HIV-1 envelope glycoprotein gp120, may be able to inhibit the entry of HIV-1 into target cells in a b12-related fashion.

**Methods:** Peptides presenting the sequences of the heavy chain CDRs of b12 were synthesized by solid-phase synthesis. The virus neutralising activity of b12 itself, as well as the paratope mimetic peptides, was tested using a reporter cell line. To further clarify the mechanism of inhibition, the proviral clone HIV-1_NL4-3_ was sequentially passaged in increasing concentrations of either b12 or paratope mimetic peptides. After obtaining resistant viruses, they were sequenced and analysed for cross-resistance against each inhibitor.

**Results:** The paratope mimetic peptides were found to specifically recognize the b12 antigen, i.e. gp120 from b12-susceptible HIV-1, but not gp120 from b12-resistant HIV-1. Furthermore, the peptides were able to inhibit HIV-1 infection in cellular infections assays, at micromolar concentrations. A peptide with mutations at key amino acids within the three CDR loops was inactive. Viruses resistant against b12 and the paratope mimetic peptide, respectively, occurred after 15 and 6 passages, respectively. b12 selected one mutation (V370E), located directly in the CD4 binding site, while the mutations induced by the paratope mimetic peptide were in the vicinity of this site. The b12-resistant virus was also resistant against the paratope mimetic peptide. Vice versa, cross-resistance was also evident, but less pronounced.

**Conclusion:** Peptides which mimic the CDRs of the broadly neutralising antibody b12, exhibit antiviral activity. Ongoing chemical and structural optimization of the peptides is expected to enhance their antiviral activity, demonstrating the therapeutic potential of antibody paratope mimics.

#### P3 Investigating cellular pathways involved in the transmission of HIV-1 between dendritic cells and T cells using RNAi screening techniques

##### Rebecca Midgley, James Wheeldon, Vincent Piguet

###### Cardiff University, Dermatology, Cardiff, Great Britain

**Correspondence:** Rebecca Midgley

*Retrovirology* 2016, **13(Suppl 1)**: P3

**Background:** Dendritic cells (DC) are thought to be amoung the earliest targets of HIV-1 infection and act as a ‘Trojan Horse’ concealing the virus from the innate immune system and delivering it directly to T-cells via virological synapses to promote infection.

**Question:** DC studies have led to the identification of several restriction factors and cellular structures that aid viral transmission, however work still needs to be done on how the virus is trafficked through the cell to the virological synapse and how the virus evades degradation within DC.

**Method:** Advancements in RNAi screening libraries allows the investigation of multiple pathways which could potential be involved in viral transmission such as cell signalling. A high-throughput method has been developed using On-Target SMART pool siRNA (Dharmacon) in Monocyte derived dendritic cells (MDDC) to investigate the transfer of HIV-1 from DC to T-cells.

**Results:** Initial membrane traffic screening results analysed using network mapping software (Cytoscape 3.3.0) implicates a role of several genes involved in vesicle mediated transport at both the plasma and vesicle membrane and a role for actin cytoskeleton organisation in DC to T-cell HIV transfer.

**Conclusion:** The discovery of potential cellular targets involved in HIV-1 transmission between DC and T-cells could potentially lead to the discovery of potential drug targets to be developed to combat HIV-1 infection in the future.

#### P4 Co-receptor tropism in HIV-1, HIV-2 monotypic and dual infections

##### Priyanka Khopkar^1,2^, Megha Rohamare^3^, Smita Kulkarni^1^

###### ^1^National AIDS Research Institute, Virology, Pune, India; ^2^Symbiosis International University, Department of Health and Biomedical Sciences, Pune, India; ^3^National Institute of Virology, Academic Department, Pune, India

**Correspondence:** Priyanka Khopkar

*Retrovirology* 2016, **13(Suppl 1)**: P4

**Background:** HIV entry is mediated through the retroviral envelope, CD4 and chemokine receptors (majorly CXCR4, CCR5). Co-receptor tropism plays a crucial role in HIV transmission and pathogenesis. HIV-1 co-receptor tropism is extensively studied; however scarce data exists on tropism exhibited during HIV-2 and HIV-1&2 dual infections from endemic regions. In the present study, we determined co-receptor tropism amongst Indian drug naïve patients with HIV-1 (n = 10); HIV-2 (n = 12) and HIV-1&2 dual infection (n = 13) confirmed by ELISA and Western Blot.

**Fig. 1:**
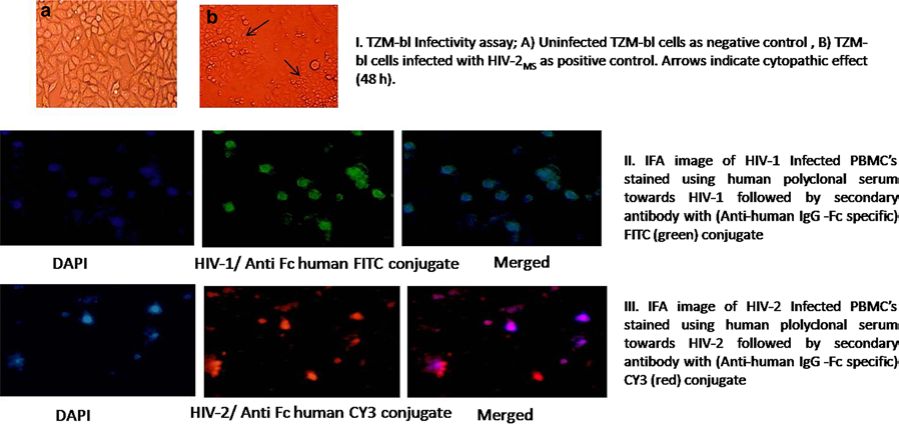
Confirmation of HIV-2 and HIV-1 and 2 virus isolations

**Methods:** PBMC co-cultures using patient’s PBMCs were carried out and virus growth confirmed by various assays viz. for i) HIV-1: HIV-1 p24 antigen capture ELISA on culture supernatants and IFA on infected cells; ii) HIV-2: ExaVir™ load, TZM-bl infectivity assay on culture supernatants for CPE and IFA on infected cells; iii) HIV-1&2 dually infected viral cultures: presence of HIV-1 was confirmed by HIV-1 p24 antigen capture ELISA; independent IFAs were carried out for HIV-1 and HIV-2 and overall infection was assessed using TZM-bl infectivity assays and ExaVir™ load (Fig. 1). Co-receptor tropism was determined using GHOST cell assays in co-receptor specific cell lines (GHOST CXCR4; GHOST CCR5); the results were confirmed microscopically for syncytium formation and on FACS using FLOWJO software for green florescence protein production under the control of HIV *tat*.

**Fig. 2:**
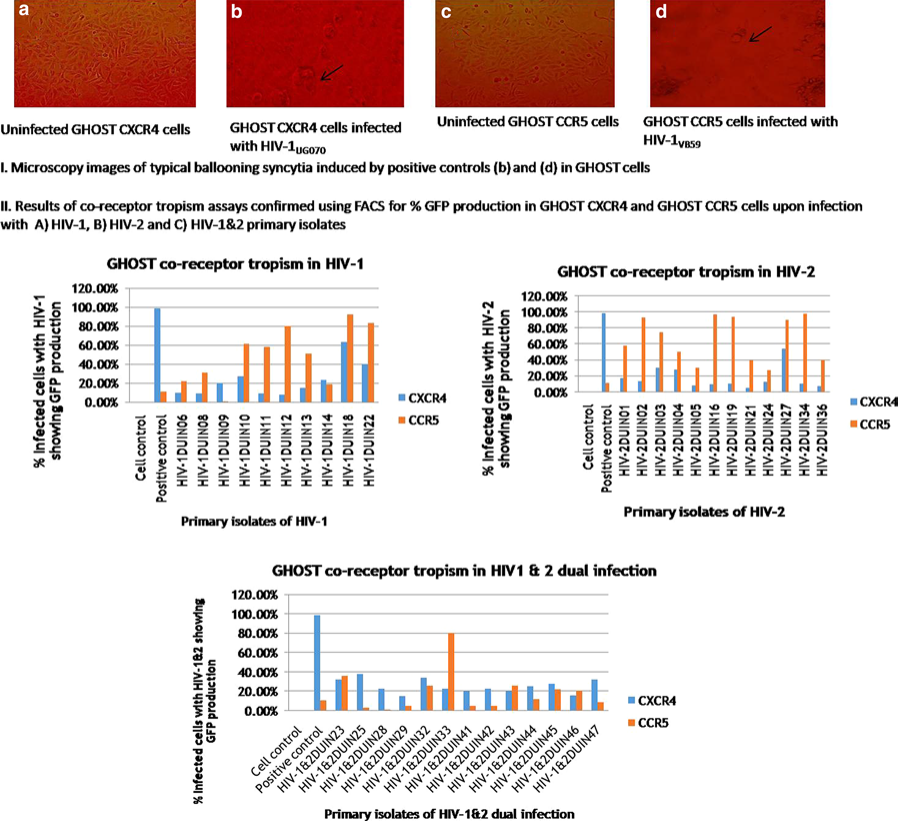
Results of co-receptor tropism

**Results:** Our results suggest that amongst ten HIV-1 drug naïve primary isolates one isolate was CXCR4 tropic, four were CCR5 tropic and five were dual tropic viruses. Of twelve HIV-2 drug naïve primary isolates, eight were CCR5 tropic and four were dual tropic. In case of HIV-1&2 dually infected primary isolates, seven were CXCR4 tropic and six were dual tropic viruses (Fig. 2).

**Conclusion:** During the early stage of HIV infection, the transmitting virus is almost invariably CCR5 tropic. In contrast to these findings our data suggests 50 % existence of dual tropism in Indian HIV-1 and HIV-2 infection. Although we report in vitro phenotypic co-receptor tropism for the first time on Indian HIV-1&2 dual infections, it is difficult to attribute our findings to the independent tropism of the two viruses, as it is a preliminary baseline finding that needs further genotypic confirmation.

#### P5 Characterisation of the role of CIB1 and CIB2 as HIV-1 helper factors

##### Ana Godinho-Santos^1^, Allan Hance^2^, Joao Goncalves^1^, Fabrizio Mammano^2^

###### ^1^Research Institute for Medicines, University of Lisbon, Lisbon, Portugal; ^2^INSERM, University Paris-Diderot, Paris, France

**Correspondence:** Fabrizio Mammano

*Retrovirology* 2016, **13(Suppl 1)**: P5

**Questions:** Understanding how cellular proteins participate in HIV-1 replication provides insights into the mechanisms of individual steps of retroviral replication, and may identify potential novel antiviral targets. In this study we evaluated the contribution of the calcium- and integrin-binding 2 (CIB2) protein, previously identified by RNAi screening as a potential helper factor, and its homolog, CIB1.

**Methods:** Jurkat cells and primary CD4+ T-lymphocytes were transduced by lentiviral vectors expressing shRNA targeting either CIB1 or CIB2, resulting in efficient and specific knockdown of expression of these proteins.

**Results:** HIV-1 replicated with significantly lower efficiency in transduced cells, identifying CIB1 and CIB2 as non-redundant HIV-1 helper factors. By exploring individual steps of virus replication in CIB1- or CIB2-knockdown cells, we determined that CIB1 and CIB2 are required for efficient HIV-1 entry into target cells, both for CCR5- and CXCR4-tropic viruses. Also, both the cell-free and cell-associated entry pathways were affected by CIB proteins depletion. In contrast, knockdown of CIB1 and CIB2 had no impact on the infectivity of HIV-1 virions pseudotyped with the VSV-G envelope in either Jurkat cells or CD4+ T-lymphocytes. We found no evidence that the level of CIB1 and CIB2 expression influenced cell viability, cell proliferation, receptor-independent viral binding to the cell surface, later steps involved in the transport and uncoating of the viral capsid, nuclear import and integration, or the production, export and infectivity of progeny virions. CIB1 and CIB2 knockdown were found to reduce the expression of surface receptors implicated in HIV-1 infection, including CCR5, CXCR4 and integrin α4β7.

**Conclusions:** In this study we have shown CIB1 and CIB2 knockdown significantly inhibited HIV-1 replication, specifically affecting the viral entry step. CIB1 and CIB2 knockdown reduced the expression of surface receptors implicated in HIV-1 infection, suggesting at least one mechanism through which these proteins may promote viral infection.

#### P6 Buffering deleterious polymorphisms in the highly constrained C2 region of HIV-1 envelope by the flexible V3 domain

##### Romain Gasser^1^, Meriem Hamoudi^1^, Martina Pellicciotta^1^, Zhicheng Zhou^2^, Clara Visdeloup^3^, Philippe Colin^2^, Martine Braibant^3^, Bernard Lagane^2^, Matteo Negroni^1^

###### ^1^Institut de Biologie Moleculaire et Cellulaire, Strasbourg, France; ^2^Institut Pasteur, Paris, France; ^3^Université François Rabelais, Tours, France

**Correspondence:** Matteo Negroni

*Retrovirology* 2016, **13(Suppl 1)**: P6

Covariation is an essential evolutionary process leading to the coevolution of parts of proteins and genomes. In organisms that are subject to strong selective pressure, coevolution is central to keep the balance between the opposite requirements of antigenic variation and retention of functionality. Being the viral component the most exposed to the environment, the envelope glycoprotein gp120 of HIV-1 constitutes the main target of the immune response raised against this virus. This is also reflected by the fact that its more external portions are characterised by extensive sequence heterogeneity leading to broad antigenic variation. We are interested in the study of coevolution within the HIV-1 envelope through the functional characterisation of chimerical envelopes of primary isolates of HIV-1 group M.

We observe that a single polymorphism, present at the level of the viral population in the conserved internal region C2, is sufficient to totally abolish Env functionality when inserted in an exogenous envelope backbone. Two main alterations of the functionality of the envelope are responsible for the loss of functionality: a decrease in the proportion of trimeric forms and a post-CCR5 binding defect, likely due to an interference with the subsequent conformational changes that lead to membrane fusion. We also observed that a complete restoration of functionality can be achieved by compensatory polymorphisms introduced at the level of the external and hypervariable region V3. Interestingly, this is accompanied by a change in antigenic profile and in the response to treatment with membrane-fusion inhibitors.

Altogether, these results indicate that (1) coevolution between V3 and C2 can control the formation of trimeric spikes on the viral particles, (2) even if two V3 loops bind CCR5 with similar affinity, the subsequent steps required to carry out membrane fusion can have markedly different outcomes, suggesting that the modes of binding can be different. (3) Finally, these results suggest that variable regions, besides harbouring intrinsic extensive antigenic diversity themselves, can also contribute to sequence diversification in more structurally constrained parts of the gp120, further increasing the genetic flexibility of the protein.

This research was supported by Sidaction and the ANRS.

#### P7 Entry inhibition of HERV-K(HML-2) by an Env-IgG fusion protein

##### Jula Wamara, Norbert Bannert

###### Robert Koch Institute, Department 1, FG18, Berlin, Germany

**Correspondence:** Jula Wamara

*Retrovirology* 2016, **13(Suppl 1)**: P7

The recognition of a cell-surface protein or a group of surface proteins and their specific interaction with the viral envelope protein represent the first stage and one of the key events of the viral infection process. For the human endogenous retrovirus HERV-K(HML-2), the cellular receptor(s) which mediate entry of the virus have not been yet identified.

We have generated a fusion protein comprising a codon-optimized version of the reconstituted envelope glycoprotein of HERV-K113 (OriCoEnvgp42) and the Fc-region of the human immunoglobulin protein (h-IgG). This fusion-protein is secreted from transfected cells and can be easily purified. OriCoEnvgp42-IgG interferes profoundly with the HERV-K(HML-2) virus/receptor interaction and inhibits virus entry in a concentration-dependent manner. In order to shed light on the receptor binding site of HERV-K(HML-2) Env, we aligned the protein sequence with the Env sequence of the most closely related Betaretrovirus, mouse mammalian tumor virus (MMTV). This allowed the putative HERV-K(HML-2) Env receptor binding site (RBS) to be predicted, based on the MMTV Env RBS. RBS mutants were then generated from a C-terminal truncated HERV-K(HML-2) Env and the OriCoEnvgp42. The RBS mutants Env_D139A and Env_Δ144–153 were expressed at wild-type levels, but in contrast to the reconstituted original Env did not facilitate entry of pseudotyped lentiviruses, indicating a defect in receptor binding. However, mutants derived from the OriCoEnvgp42-IgG fusion protein still interfered with the entry of reporter viruses pseudotyped with the reconstituted HERV-K(HML2) Env.

These data suggest the presence of additional, relevant RBS sequences and should help the complete RBS of HERV-K(HML-2) to be characterised. The OriCoEnvgp42-IgG fusion protein will be a very useful tool in elucidating the mechanisms of cell-entry by the virus.

### Topic 2: Reverse transcription & integration

#### P8 The R263K/H51Y resistance substitutions in HIV integrase decreases levels of integrated HIV DNA over time

##### Thibault Mesplede, Nathan Osman, Kaitlin Anstett, Jiaming Calvin Liang, Hanh Thi Pham, Mark Wainberg

###### Jewish General Hospital, Lady Davis Institute, McGill AIDS Centre, Montreal, Canada

**Correspondence:** Mark Wainberg

*Retrovirology* 2016, **13(Suppl 1)**: P8

**Background:** HIV DNA that is integrated into cells can persist indefinitely within HIV-positive individuals, even when they are successfully treated with antiretroviral therapy (ART). This persistence of integrated HIV DNA within reservoirs contributes to an inability to achieve viral eradication.

No patient treated with the integrase inhibitor dolutegravir (DTG) in first-line therapy has ever developed resistance to this drug. Our group showed in culture that DTG can select for a R263K mutation in Integrase that confers low-level DTG resistance. Although rare, failure in treatment-experienced, integrase inhibitor-naïve individuals who are treated with DTG is associated with the emergence of the R263K substitution in integrase as well as plasma viral loads that are lower than those observed when treatment failure occurs with ART regimens that do not contain DTG. This is likely due to the fact that R263K confers only low-level resistance against DTG and also decreases both viral replication capacity and viral integrase activity in short-term infectivity assays. We sought to determine the effect of the DTG-specific R263K resistance substitution on integration during long-term infection.

**Methods:** We measured HIV integration by Alu-mediated QPCR over 5 weeks of infection of Jurkat cells with WT, R263K and H51Y/R263K viruses. Levels of integration were measured every week and expressed relative to integration of the WT virus after week 1. Means ± standard deviations were calculated and Student’s *t* test was used to evaluate significance of differences.

**Results:** The R263K substitution impaired HIV integration over time and was associated with a progressive decline in levels of integrated HIV DNA in peripheral blood mononuclear cells. Even further impairments were noted if both the R263K and H51Y substitutions were simultaneously present and this is because H51Y further impairs viral replication and integrase activity at the same time that it only slightly increases levels of drug resistance against DTG.

**Conclusions:** This raises the possibility that emergence of the R263K/H51Y substitutions in individuals who experience treatment failure with DTG might result in a progressive decline in the size of the viral reservoir. Further studies to study this hypothesis are underway in SIV-infected macaques.

#### P9 The Retrovirus Integration Database (RID)

##### Wei Shao^1^, Jigui Shan^1^, Mary Kearney^2^, Xiaolin Wu^3^, Frank Maldarelli^2^, John Mellors^4^, Brian Luke^1^, John Coffin^5^, Stephen Hughes^2^

###### ^1^Leidos Biomedical Research, Inc, Advanced Biomedical Computing Center, Frederick, MD, United States; ^2^National Cancer Institute, HIV Dynamics and Replication Program, Frederick, MD, United States; ^3^Leidos Biomedical Research, Inc, Frderick National Lab for Cancer Research, Frederick, MD, United States; ^4^University of Pittsburgh, Division of Infectious Disease, Pittsburgh, PA, United States; ^5^Tuffs University, Boston, MA, United States

**Correspondence:** Wei Shao

*Retrovirology* 2016, **13(Suppl 1)**: P9

Retrovirus replication requires that the virus integrate a DNA copy of its genome into the host chromosomal DNA. Although there are numerous published studies that describe the distribution of retrovirus integration sites, there is no large publicly available centralized database that contains the available integration site information. Currently, most of the retrovirus integration site information is found in supplementary materials, which makes retrieving it for meta-analyses difficult. Thus, a comprehensive database that includes information about integration sites is critically needed.

**Fig. 3:**
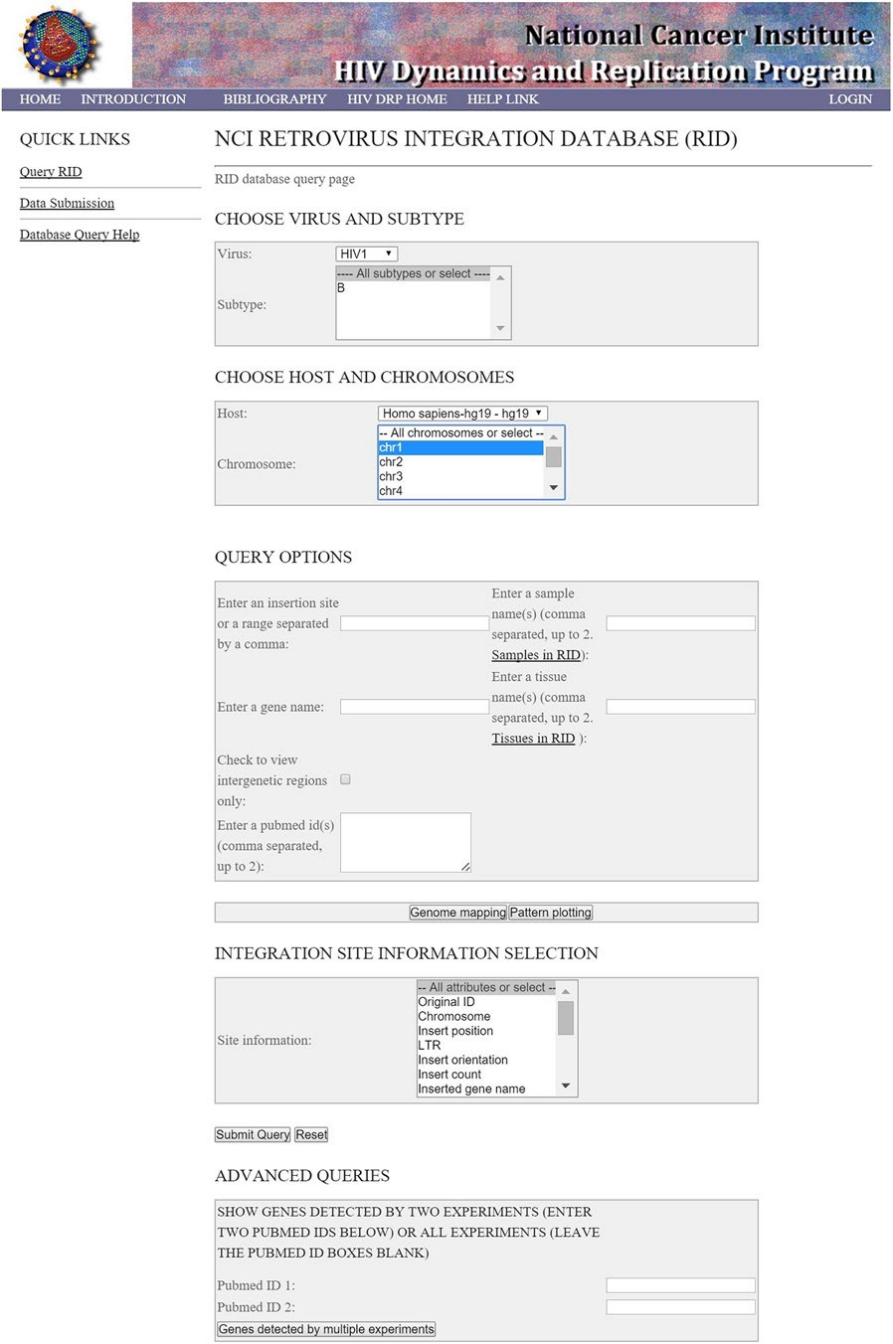
NCI Retrovirus Integration Database

**Fig. 4:**
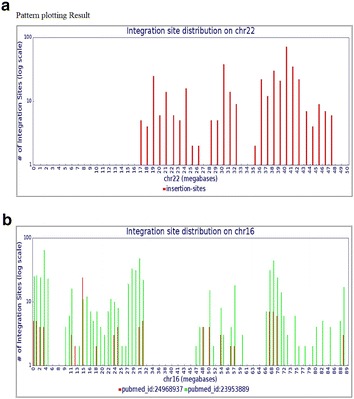
Pattern plotting result. **a** One dataset, **b** comparison of two datasets

We have built the NCI Retrovirus Integration Database (RID, Fig. 3) to record integration site information for all retroviruses, including HIV-1, HTLV, and MLV. RID is an in-progress MySql based relational database. Briefly, it has tables to store host, virus and subtypes, sample/patient and tissue and demographics information without (for integration sites in humans) revealing personally identifiable information. Chromosome, integration site, associated genes, exon/intron information, provirus orientation, and the references from which the information was collected are provided on the database. Additionally, we built several tools into the database to facilitate mapping of the integration sites to USCS genome browser, to plot the integration site patterns on a chromosome (Fig. 4), and to display provirus LTRs in their inserted genome sequence for PCR/probe design. We also created a robust, user friendly website that allows users to query the database and analyze the data dynamically. All the integration sites are mapped to human genome build hg19 for easy comparison between different datasets.

In conclusion, we have created a relational database to store comprehensive retrovirus integration information. This information will facilitate the retrieval and analysis of the published integration datasets. The database is available for public use. For a link, see https://rid.ncifcrf.gov.

#### P10 The small molecule 3G11 inhibits HIV-1 reverse transcription

##### Thomas Fricke^1,2^, Silvana Opp^1^, Caitlin Shepard^3^, Dmitri Ivanov^4^, Baek Kim^3^, Jose Valle-Casuso^1^, Felipe Diaz-Griffero^1^

###### ^1^Albert Einstein College of Medicine, Department of Microbiology & Immunology, New York City, NY, United States; ^2^International Institute of Molecular and Cell Biology, Laboratory of Structural Biology, Warszawa, Poland; ^3^Emory University, Department of Pediatrics, Atlanta, GA, United States; ^4^University of Texas Health Science Center, Department of Biochemistry, San Antonio, TX, United States

**Correspondence:** Thomas Fricke

*Retrovirology* 2016, **13(Suppl 1)**: P10

**Fig. 5:**
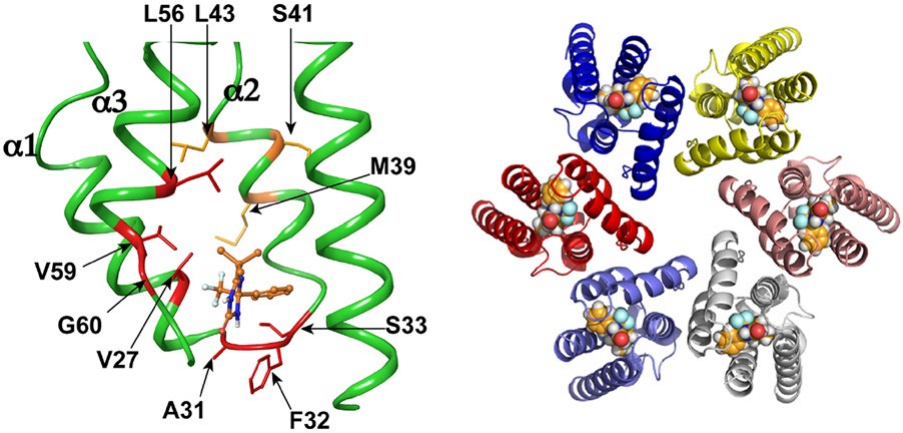
(*Left*) model of 3G11 bound to HIV-1 capsid. Most affected residues are shown in *red* and less affected in *orange*. (*Right*) model of the 3G11-bound CA hexamer viewed from the interior of the core particle

**Fig. 6:**
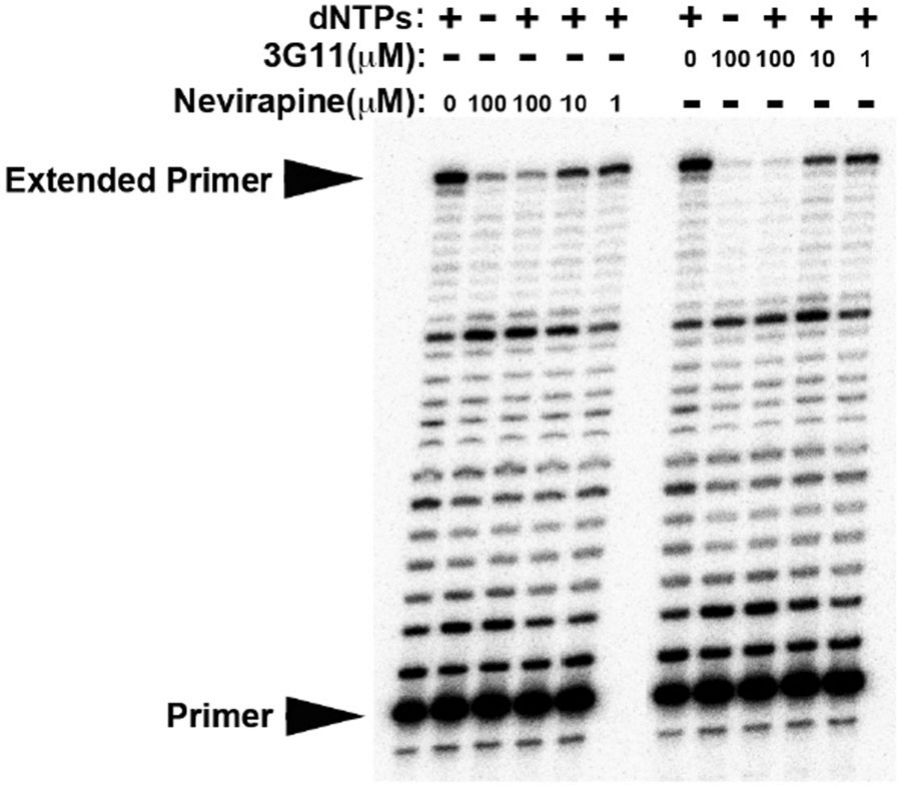
3G11 blocks HIV-1 reverse transcriptase activity. 32P-labeled 17-mer primer annealed to 40-mer RNA template was extended by HIV-1 RT with varying concentrations of 3G11, Nevirapine was also used for comparison

The small molecule 6-(tert-butyl)-4-phenyl-4-(trifluoromethyl)-1H,3H-1,3,5-triazin-2-one (3G11) was discovered as a small molecule that potentially targets capsid and inhibits infection of replication competent HIV-1 on a cell-based screen using the T cell line MT-4. Here we showed that 3G11 specifically and potently blocks HIV-1 infection. By contrast, 3G11 did not block other related retroviruses such as HIV-2, simian immunodeficiency virus (SIVmac), bovine immunodeficiency virus (BIV), feline immunodeficiency virus (FIV), equine infectious anemia virus (EIAV), N-tropic murine leukemia virus (N-MLV), B-tropic murine leukemia virus (B-MLV) and Moloney murine leukemia virus (Mo-MLV). Although NMR experiments revealed that 3G11 binds to the HIV-1 capsid (Fig. 5), functional experiments (fate of the capsid assay and capsid stability assay) suggested that capsid is not the viral determinant for sensitivity to 3G11. Analysis of DNA metabolism by real-time PCR revealed that 3G11 blocks the formation of HIV-1 late reverse transcripts during infection. In agreement, an in vitro primer extension assay revealed that 3G11 blocks the enzymatic activity of HIV-1 reverse transcriptase as strong as nevirapine (Fig. 6) Overall, we described a novel non-nucleoside reverse transcription inhibitor (NNRTI) that blocks HIV-1 infection.

#### P11 Dual and opposite regulation of HIV-1 integration by hRAD51: impact on therapeutical approaches using homologous DNA repair modulators

##### Vincent Parissi^1,2^

###### ^1^CNRS, UMR5234 MFP Lab, Bordeaux, France; ^2^Associated international laboratory (LIA) microbiology and Immunology, CNRS/Université de Bordeaux/Heinrich Pette Institute-Leibniz Institute for Experimental Virology, Bordeaux/Hamburg, France

**Correspondence:** Vincent Parissi

*Retrovirology* 2016, **13(Suppl 1)**: P11

**Background:** HIV-1 integration is regulated by cellular cofactors acting at early and late steps of the process. The cellular DNA repair recombinase hRAD51 has been shown to interact with HIV-1 integrase and restrict integration both in vitro and in vivo. This finding paved the way for the development of new antiviral strategies based on the stimulation of hRAD51 recombination activity. However, hRAD51 has also been shown to stimulate HIV-1 expression by enhancing LTR transcription. This complicates any therapeutic strategies based on hRAD51 modulation without pre-existing knowledge of the regulatory functions of this recombinase in HIV-1 replication. In order to better determine the role of hRAD51 in virus replication, we here performed biochemical and pharmacological analyses on its regulatory activity during the step of HIV-1 integration.

**Fig. 7:**
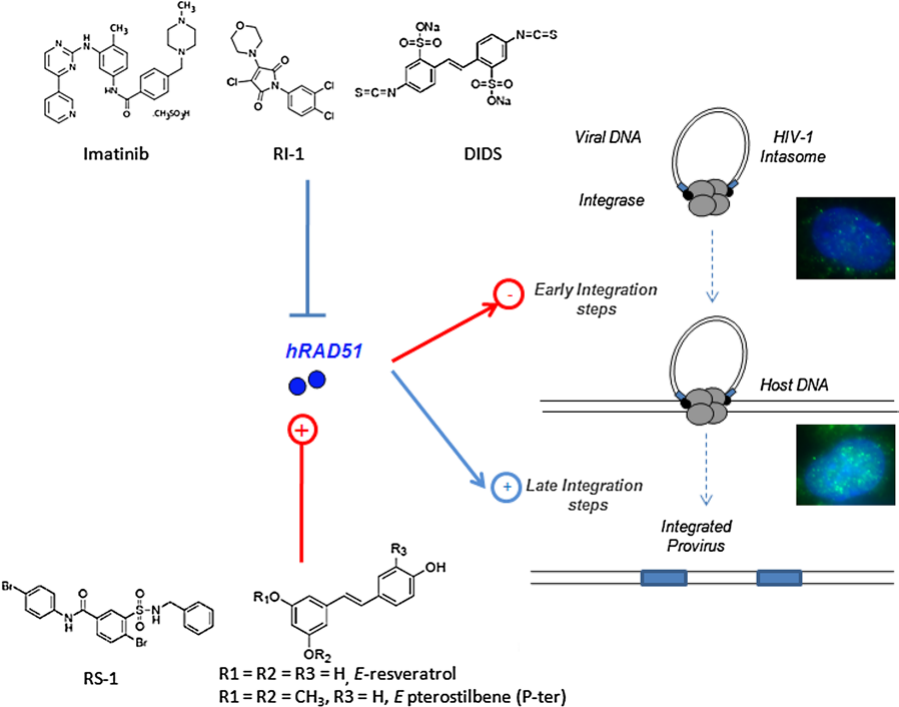
Activation of hRAD51

**Results:** We show here in in vitro experiments that activation of hRAD51 inhibits the viral integrase. This effect on integrase activity is abolished when the recombinase is inhibited. This indicates that hRAD51-mediated inhibition of integration is closely linked to the promotion of DNA repair and recombination activities of the protein. Interestingly pharmacological cellular analyses demonstrate that cells with high intracellular hRAD51 concentrations or activity prior *de novo* infection are more resistant to early steps of the integration process whereas when hRAD51 was activated during integration this step was strongly promoted (Fig. 7).

**Conclusions:** The presented data indicate an unexpected opposite regulatory function of hRAD51 on early and late stages of the integration process. In addition to providing new information about the regulation of HIV-1 integration by hRAD51 our data may form the basis for improved and novel antiviral approaches aiming to modulate intracellular hRAD51 activity or concentrations.

#### P12 A flexible motif essential for integration by HIV-1 integrase

##### Marine Kanja, Pierre Cappy, Matteo Negroni, Daniela Lener

###### Institut de biologie moléculaire et cellulaire, Strasbourg, France

**Correspondence:** Marine Kanja

*Retrovirology* 2016, **13(Suppl 1)**: P12

Carrying out the integration of the viral DNA into the host cell genome, HIV-1 integrase (IN) is crucial for viral replication. As such, it constitutes an appealing target for the development of antiviral drugs. However, the architecture of the IN tetramer has so far only been inferred by combining information obtained on individual parts of the protein. Retention of functionality in proteins presenting sequence variability, as HIV proteins, relies on the existence of coevolution networks that allow counterbalancing the potential deleterious effects of one mutation by the introduction of compensatory mutations in other positions of the protein. Since the positions harbouring the mutations are structurally and functionally related, this provides information about the arrangement of the protein.

We study coevolution networks in the IN in order to understand the architecture of the tetramer. To this end, we constructed chimerical IN between primary isolates of HIV-1 groups M and O and analysed their functionality by infection in culture. The rationale is that, if coevolution networks were perturbed, functionality could be impaired. We observed a decrease of integration efficiency for certain IN chimeras. Systematic replacement of residues that differ between wt and chimerical IN defined a motif of 4 residues, constituted of 2 K residues and 2 polar amino acids, that is required for integration. The motif is conserved within group M and within group O but differs between the two groups. When less than 2 K are present, the functionality of the protein is severely impaired, while for 2 or more K functionality is restored, irrespective of their positions. Structural data suggest that they could interact with the target DNA.

These results are relevant from at least two standpoints. One is the definition of an essential motif for IN functionality that could be responsible for the recognition of the cellular DNA. The other is the remarkable possibility of permutation of the position where the K residues can be present without affecting IN functionality. This feature allows conciliating sequence diversification in HIV-1 and preservation of functionality. Establishing the function of these motifs appears now essential for improving our understanding of the mechanisms of HIV integration.

This research was supported by Sidaction.

#### P13 Interaction between HIV-1 integrase and the host protein Ku70: identification of the binding site and study of the influence on integrase-proteasome interplay

##### Ekaterina Knyazhanskaya^1^, Andrey Anisenko^2^, Timofey Zatsepin^1^, Marina Gottikh^3^

###### ^1^Lomonosov Moscow State University, Chemical Department, Moscow, Russian Federation; ^2^Lomonosov Moscow State University, Faculty of bioengineering and Bioinformatics, Moscow, Russian Federation; ^3^Lomonosov Moscow State University, Belozersky Institute of Physical and Chemical Biology, Moscow, Russian Federation

**Correspondence:** Ekaterina Knyazhanskaya

*Retrovirology* 2016, **13(Suppl 1)**: P13

The human Ku heterodimer consists of two subunits: Ku70 and Ku80. Its main function is the binding of DNA ends produced by double-strand DNA brakes during the first steps of the NHEJ repair process. Ku can also take part in transcription regulation, telomere maintenance, protein turn-over and cytoplasmic DNA-sensing. Reportedly, Ku participates in the HIV-1 replication and favors different stages of the HIV-1 life cycle, such as the formation of 2-LTR circles, integration and transcription of proviral DNA. Viral replication is reduced in cells depleted of either component of Ku and this effect is more pronounced during the early stages of viral replication (Zheng 2011; Manic 2013). However, an exact mechanism by which Ku affects the replication of HIV-1 is unclear. It has been proposed, that the binding of Ku70 to HIV-1 integrase (IN) protects the latter from proteasomal degradation.

**Fig. 8:**
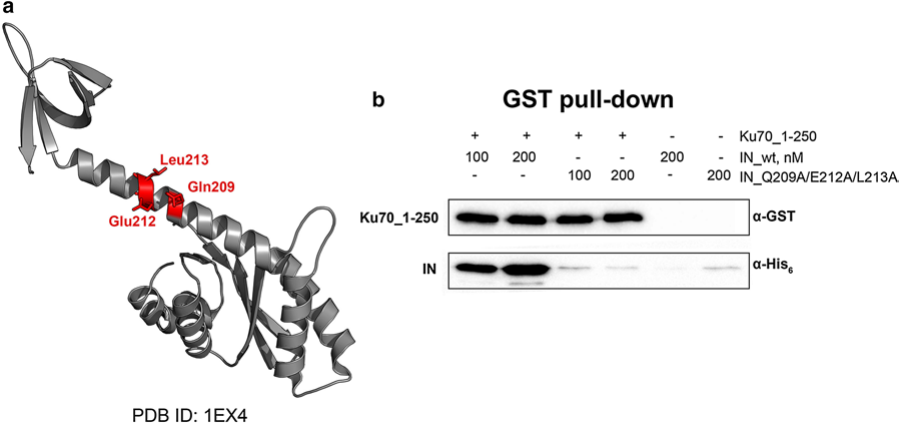
Triple HIV-1 IN mutant Q209A/E212A/L213A loses its ability to bind Ku70. **a** IN catalytic and C-terminal domains with mutated amino acids marked in *red*. **b** Western Blot analysis of the complex formation between recombinant GST-Ku70(1–250) and two recombinant His_6_-IN variants: WT and mutant

We have shown that a stable complex can be formed between recombinant Ku70 and IN with a K_d_ ~ 70 nM. Using a set of *E. coli* expressed deletion mutants of both Ku70 and IN and the GST pull-down assay we localized the binding sites within both proteins. The binding of Ku70 with IN relies at least on two sites in the proteins structure. Specifically, the Ku70(1–250) contacts with an α-helix located in the 160–230 IN region. This observation is supported by the fact that IN with alanine substitutions in positions Q209, E212 and L213 shows a weaker binding with Ku70 and does not bind Ku70(1–250) completely (Fig. 8).

The data obtained from experiments on recombinant purified proteins were confirmed by expressing C-terminal HA-tagged full-length IN and its various deletion mutants in HEK 293T cells with or without a WT Ku70-3FLAG and truncated variants. The coexpression with Ku70 stabilized IN, while the IN expression in cells that were knocked down of Ku70 was reduced. Also, the overexpression of Ku70 diminishes the association of IN with 20S proteasome as shown by immunoprecipitation of transfected cell lysates.

The work was supported by RFBR grant 14-04-00833

#### P14 Normalisation based method for deep sequencing of somatic retroelement integrations in human genome

##### Alexander Komkov^1^, Anastasia Minervina^1^, Gaiaz Nugmanov^1^, Vadim Nazarov^2^, Konstantin Khodosevich^3^, Ilgar Mamedov^1^, Yuri Lebedev^1^

###### ^1^Shemyakin-Ovchinnikov Institute of Bioorganic Chemistry of the Russian Academy of Sciences, Moscow, Russian Federation; ^2^National Research University Higher School of Economics, Moscow, Russian Federation; ^3^University of Copenhagen, Copenhagen, Denmark

**Correspondence:** Anastasia Minervina

*Retrovirology* 2016, **13(Suppl 1)**: P14

**Fig. 9:**
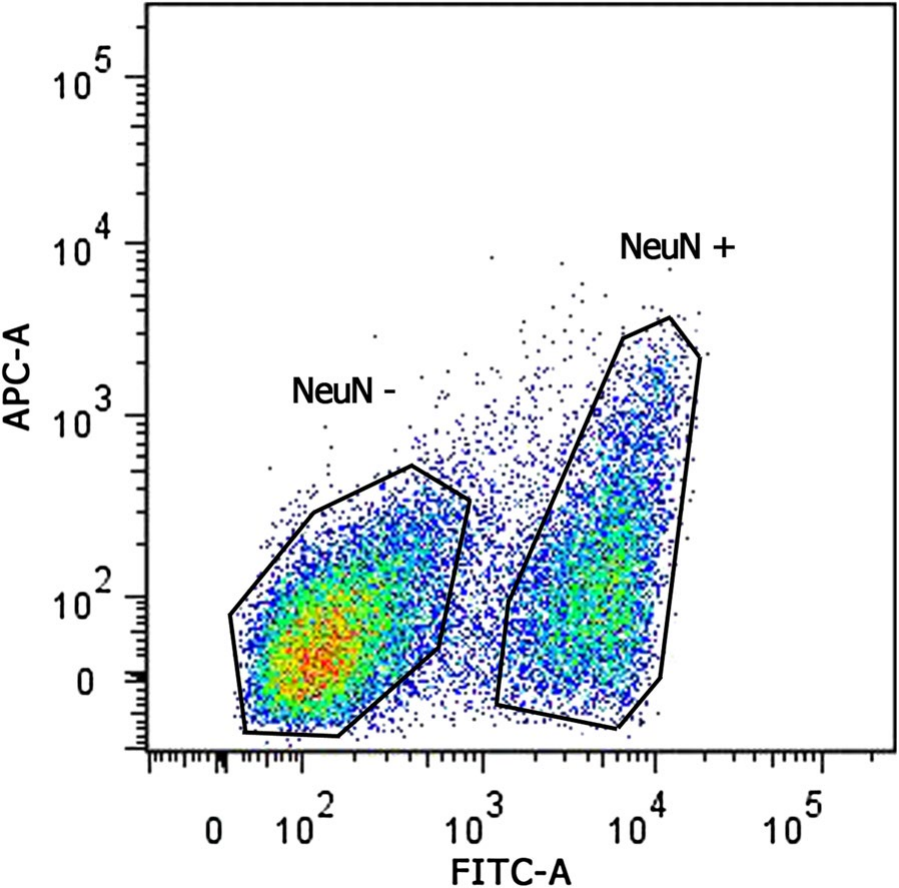
Immunofluorescent sorting of nuclei from human dentate gyrus

**Fig. 10:**
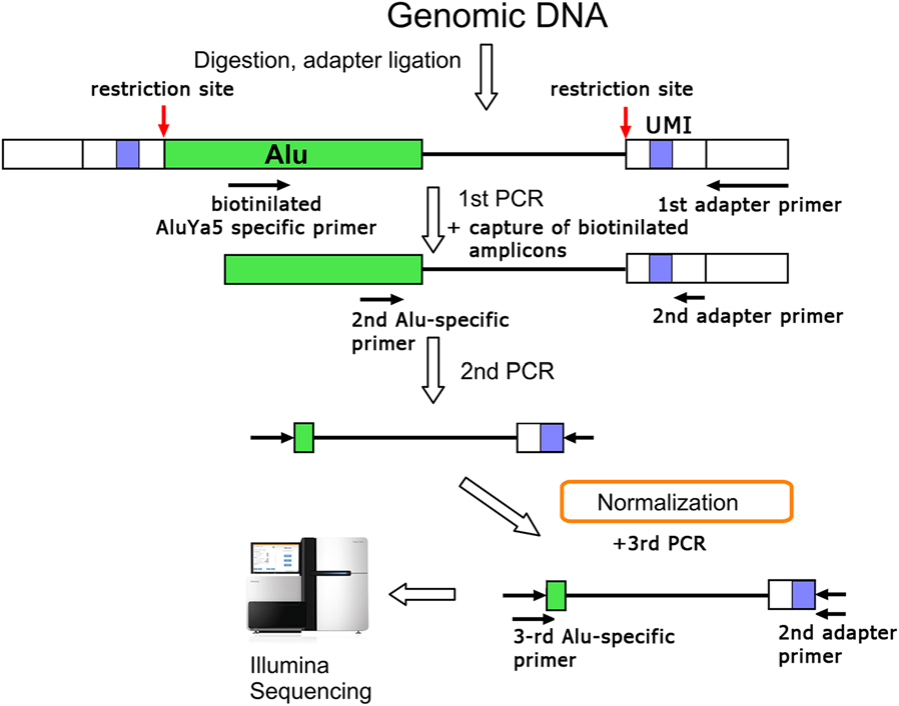
Scheme of DNA libraries preparation

Retroelements’ (RE) activity is a huge source of genetic innovation in human somatic cells. At the present time somatic RE insertions were found in normal and malignant cells of different origin. Despite of rapid development of high-throughput sequencing technologies all current methods for somatic RE insertions identification still have two major limitations: (1) ability to analyze only small number of cells due to low enrichment of DNA library by target sequences; (2) high rate of false positive results because of ligation and amplification procedures. In this study we developed a new method for identification of rare somatic RE insertions in human genome. Applying of DNA Normalisation procedure with duplex-specific DNAse from Kamchatka Crab provides additional (to PCR or capture enrichment) 20× enrichment for somatic insertions. Fragmentation genomic DNA before amplification by restriction nucleases instead sonication enables accurate filtering of false positive. Introduction of Unique Molecular Identifiers (UMI) in each target molecule before all steps of enrichment enables to unambiguously quantify the number of cells bearing a certain somatic insertion. This method was used for identification of somatic insertions of AluYa5 elements in 25,000 nuclei from NeuN+ (neurons) and NeuN− (glia) fraction from human dentate gyrus. We found 32 highly confident somatic insertions in NeuN+ fraction and 84 somatic insertions in NeuN− fraction. Our results show that somatic insertions of AluYa5 elements occurs only in small percent of cells and probably more frequently in glia cells then in neurons. Thus, the developed method could be employed to identify somatic insertions which are present in a small subpopulation of cells (Figs. 9, 10).

*The reported study was funded by RFBR according to the research project No.* 16-34-01100-mol_a and No. 16-04-00779 and Russian President’s Fallowship No. SP-4059.2016.4.

### Topic 3: Transcription and latency

#### P15 BCA2/RABRING7 restricts HIV-1 transcription by preventing the nuclear translocation of NF-κB

##### Marta Colomer-Lluch, Ruth Serra-Moreno

###### Texas Tech University, Biological Sciences, Lubbock, TX, United States

**Correspondence:** Ruth Serra-Moreno

*Retrovirology* 2016, **13(Suppl 1)**: P15

BCA2 (breast cancer-associated gene 2) is an E3 ubiquitin ligase that serves as a co-factor in the restriction imposed by Tetherin on HIV-1. We recently demonstrated that BCA2 also has Tetherin-independent activity. In particular, BCA2 targets HIV-1 Gag for lysosomal degradation, impairing virus assembly. Since many antiviral factors modulate the NF-κB pathway, we sought to explore if BCA2 is harnessing this innate signaling cascade to further limit HIV-1.

Here we show for the first time that *BCA2* is induced by NF-κB-activating cytokines and that its up-regulation provides a negative feedback on NF-κB. Mutagenesis analyses indicated that the catalytic domain of BCA2 is critical to suppress NF-κB signaling. Besides being an E3 ubiquitin-ligase, BCA2 may also act as an E3 SUMO-ligase, since it physically interacts with UBC9—an E2 SUMO enzyme. UBC9 mediates the SUMOylation of IκBα, which in turn impairs the nuclear translocation of NF-κB. To explore if BCA2 participates in this process, we assessed IκBα’s post-translational modifications and the subcellular distribution of NF-κB components. Remarkably, the levels of SUMOylated IκBα increased in cells overexpressing BCA2 whereas its phosphorylation levels diminished. Conversely, depletion of UBC9 or BCA2 led to a significant reduction of SUMOylated IκBα and a corresponding increase in its phosphorylation levels. *In vitro* SUMOylation studies revealed that BCA2 enhances IκBα SUMOylation, demonstrating for the first time that BCA2 serves as a SUMO-ligase in the regulation of the NF-κB pathway. Consistent with this, BCA2 blocked the nuclear translocation of NF-κB.

**Fig. 11:**
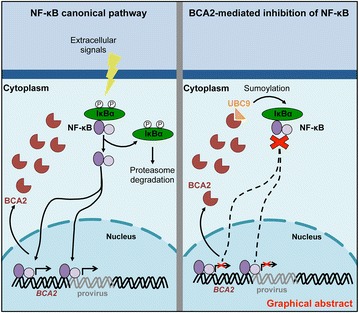
Biological implication of the BCA2

Since HIV-1 needs NF-κB to enhance its replication, we examined the biological implication of the BCA2-dependent inhibition of this pathway in HIV-1 infectivity. BCA2 reduced the transcriptional activity of HIV-1 by twofold 6 h post-infection and this effect was more pronounced at later time points. In fact, the BCA2-mediated inhibition of NF-κB accounts for 70 % of its overall antiviral activity and causes a fivefold defect in virus replication. Thus, our findings demonstrate that BCA2 is an important barrier to HIV-1 by affecting multiple steps of its replication cycle. Not only does BCA2 prevent assembly and release of nascent virions, but also impairs HIV-1 at the transcriptional level (Fig. 11).

#### P16 MATR3 post-transcriptional regulation of HIV-1 transcription during latency

##### Ambra Sarracino^1^, Anna Kula^2^, Lavina Gharu^1^, Alexander Pasternak^3^, Carine Van Lint^2^, Alessandro Marcello^1^

###### ^1^ICGEB, Trieste, Italy; ^2^Université Libre de Bruxelles, Belgium, Belgium; ^3^University of Amsterdam, CINIMA AMC, Amsterdam, Netherlands

**Correspondence:** Alessandro Marcello

*Retrovirology* 2016, **13(Suppl 1)**: P16

Post-transcriptional regulation of HIV-1 replication is finely controlled by viral and host factors. Among the former, Rev controls the export of unspliced viral RNAs from the nucleus and their expression in the cytoplasm or incorporation into new virions. Taking advantage of a novel proteomic approach we previously identified the nuclear matrix protein MATR3 as a Rev co-factor (Kula et al. Retrovirology 2011; Kula et al. Virology 2013). To investigate the functional role of MATR3 in viral replication we depleted or over-expressed MATR3 in Jurkat cells and primary PBLs in the context of HIV infection. We confirmed that MATR3 is a positive regulator of viral replication acting at a post-transcriptional level by affecting nuclear-cytoplasmic translocation of Rev-dependent transcripts. By applying the same approach to J-Lat cells, a well-established model for the study of latency, we observed that MATR3 depletion was not affecting transcriptional reactivation of the integrated provirus upon TNFα stimuli, but was causing a defect in Gag production. Following these observations, we hypothesized that MATR3 could be involved in the establishment of HIV-1 post-integrative latency. Indeed, mechanisms acting at the post-transcriptional level have been greatly overlooked in favour of transcriptional pathways. Experiments in resting PBLs confirmed that MATR3 was almost undetectable in resting PBLs but could be promptly upregulated upon cellular stimulation with PHA. However, drugs such as SAHA or DSF, which are potent transcriptional activators of HIV-1 transcription, were poor inducers of MATR3 providing a rationale for their inability to fully reactivate the virus. These data have been confirmed in cells derived from patients under cART and are being evaluated in a model of latency based on direct infection of resting PBLs.

#### P17 HIV-1 tat intersects the SUMO pathway to regulate HIV-1 promoter activity

##### Ann Marie McCartin, Anurag Kulkarni, Valentin Le Douce, Virginie Gautier

###### University College Dublin, School of Medicine, College of Health and Agricultural Sciences, UCD-Centre for Research in Infectious Diseases, Dublin, Ireland

**Correspondence:** Ann Marie McCartin

*Retrovirology* 2016, **13(Suppl 1)**: P17

**Introduction:** To comprehensively characterise the intricate role of the viral-host interface in HIV-1 gene expression and silencing, we performed a series of system-wide proteomic screenings, which collectively revealed cross-talk between HIV-1 Tat, the key viral regulatory protein and transactivator, and the SUMOylation system. Given that Small Ubiquitin-like Modifier (SUMO) is a reversible covalent post-translational modification (PTM) targeting chromatin-associated factors and regulating epigenetic silencing, we investigated the mechanisms by which HIV-1 Tat could intersect with the SUMO pathway to promote HIV-1 gene reactivation.

**Results:** The HIV-1 promoter is dynamically associated with SUMO. We investigated the SUMO chromatin profile of the integrated HIV-1 Promoter in J-LAT cells using ChIP-qPCR. Under basal conditions, the silenced HIV-1 promoter is enriched in SUMO-1 and SUMO-2/3, while TNF-alpha stimulation or SAHA-mediated de-repression resulted in a decrease of SUMO-1 and SUMO-2/3 from the HIV-1 promoter.

SENP3 is a co-factor of Tat-mediated reactivation of the HIV-1 promoter. We previously identified SENP3, a cysteine protease that catalyses SUMO removal, to be part of the Tat nuclear interactome. Here, we describe that SENP3 depletion has no impact on basal HIV-1 gene expression in J-LAT models, while SENP3 knockdown impaired HIV-1 promoter reactivation by up to 40 % in a Tat-dependent context.

Tat physically recruits SENP3 to the HIV-1 promoter. Co-immunoprecipitation in the presence and absence of SUMO protease inhibitors suggests that SUMO PTMs enhance the stability of the Tat-SENP3 complex formation. In parallel, ChIP-qPCR revealed that Tat promotes SENP3 association with the HIV-1 promoter, where it can control the level of SUMO2/3 associated with the HIV-1 promoter.

**Conclusion:** Our findings expand the repertoire of PTMs at the HIV-1 LTR and suggest that HIV gene silencing can be regulated by dynamic SUMOylation at the epigenetic level. In this context, we propose that Tat intersects with the SUMO pathway via interacting and recruiting SENP3 to the HIV-1 promoter, where it can de-SUMOylate chromatin associated factors and support Tat-mediated transactivation of the HIV promoter.

#### P18 Conservation in HIV-1 Vpr guides tertiary gRNA folding and alternative splicing

##### Ann Baeyens^1^, Evelien Naessens^1^, Anouk Van Nuffel^1^, Karin Weening^1^, Anne-Marie Reilly^1^, Eva Claeys^1^, Wim Trypsteen^2^, Linos Vandekerckhove^2^, Sven Eyckerman^3^, Kris Gevaert^3^, Bruno Verhasselt^1^

###### ^1^Ghent University, Clinical Chemistry, Microbiology, and Immunology, Ghent, Belgium; ^2^Ghent University and Ghent University Hospital, Internal Medicine, Ghent, Belgium; ^3^VIB Medical Biotechnology Center, Ghent, Belgium

**Correspondence:** Ann Baeyens

*Retrovirology* 2016, **13(Suppl 1)**: P18

Vpr is a pleiotropic accessory protein, dispensable for HIV-1 propagation in T cell lines, but important to establish infection of resting cells, like macrophages. Despite this apparent redundancy, Vpr is highly conserved among different isolates. To study Vpr-host protein interactions in a fully replicating virus, we constructed an NL4-3 HA/FLAG-Vpr virus. Surprisingly, viral production and replication were defective, due to aberrant splicing of genomic RNA. This defect was not protein-but RNA-based and sequence dependent, thus proposes that not only protein, but also RNA sequence conservation is imposed on the Vpr encoding region of HIV. Simulation of genomic RNA folding suggests that introduction of the tag sequence induced an alternative folding structure in a region enriched in splice sites and splicing regulatory sequences. To test this, alternative tagging strategies were evaluated in silico and NL4-3 HA/His6-Vpr was selected as a valid alternative. Indeed, in vitro infectivity and mRNA splice pattern improved although did not return to wild-type values. This implies that sequence-specific modifications may interfere with tertiary mRNA folding to skew the alternative splicing balance. To test if tertiary mRNA folding is conserved in the RNA sequence, we studied NL4-3 Vpr U213C, a silent mutation in all three reading frames. The U213 site is 99 % conserved and its mutation affected mRNA folding, mRNA splicing balance and infectivity. In long-term culture, this mutation reversed, which restored infectivity. From these results we conclude that sequence conservation in Vpr preserves tertiary mRNA folding, important to balance viral splicing and replication.

#### P19 The majority of reactivatable latent HIV are genetically distinct

##### Hoi Ping Mok, Nicholas Norton, Axel Fun, Jack Hirst, Mark Wills, Andrew Lever

###### University of Cambridge, Department of Medicine, Cambridge, Great Britain

**Correspondence:** Hoi Ping Mok

*Retrovirology* 2016, **13(Suppl 1)**: P19

The clonality of latent HIV has been inferred from viral sequences derived from various sources, including proviruses, residual viraemia of patients stable on treatment, and rebound viraemia in patients who have undergone treatment interruption. It has also been deduced from the integration sites of infected cells. It is unclear whether these data accurately reflect reactivatable latent viruses.

We studied viruses reactivated from latently infected cells. Resting CD4 T cells isolated from a patient stable on treatment underwent limiting dilution and were subsequently activated with PHA, IL-2 and irradiated PBMC followed by co-culture with SupT1-CCR5 feeder cells for 21 days. The supernatant was harvested for viral RNA. Amplicons were generated from a region in Gag and one in Env and analysed by Sanger sequencing. To control for sequence variations acquired during culture, SupT1-CCR5 cells were infected with NL4-3 and underwent the same limiting dilution, culture, RNA isolation and sequencing. Pairwise comparisons were performed to obtain p-distances. The p-distances obtained from NL4-3 infected SupT1-CCR5 cells were used as references. Each pair of patient derived viral sequences was considered different if the p-distance was higher than that of the corresponding region of the reference sequences.

We obtained 32 sequences of reactivated latent viruses from a single patient.19 distinct sequences could be distinguished from the Gag region. The remaining 13 sequences segregated into five groups containing up to four sequences. However, when the Env regions of these 13 sequences were analysed, only one ‘clonal’ group of two sequences remained. 30/32 reactivated latent viruses were distinct. If the threshold p-distance for two sequences to be considered distinct was set at the maximal (rather than average) p-distance observed in the reference set, 26/32 of reactivated latent viruses could still be considered distinct.

These data suggest that the majority of reactivatable latent viruses are genetically distinct. Our data show that the phylogenetic structure of reactivatable latent viruses is wholly different from that of residual viraemia, where a single ‘predominant plasma clone’ can account for over 50 % of all sequences observed.

#### P20 Do mutations in the *tat* exonic splice enhancer contribute to HIV-1 latency?

##### Nicholas Norton, Hoi Ping Mok, Jack Hirst, Andrew Lever

###### University of Cambridge, Department of Medicine, Cambridge, Great Britain

**Correspondence:** Nicholas Norton

*Retrovirology* 2016, **13(Suppl 1)**: P20

Latent infection of long-lived memory CD4+ T cells is a major barrier to the eradication of HIV-1. Reactivation of these transcriptionally silent proviruses is the basis of therapeutic approaches aimed at cure, but despite maximal activation a significant proportion of viruses are not reactivated from latency (Ho et al. 2013).

We examined published sequences from non-induced proviruses found in patient samples. All sequences contained mutations in a recently described exonic splice enhancer (ESE) involved in the regulation of *tat* mRNA splicing (Erkelenz et al. 2015). By comparison with over 2000 subtype B sequences deposited in the Los Alamos database we identified mutations that are highly enriched in the latent sequences. We hypothesised that mutations in this region could result in inefficient splicing of *tat* mRNA preventing the establishment of the Tat-TAR feedback loop and leading to silencing.

One of the mutations corresponding to a G to A mutation at position 5817 in HXB2 was found in 11/18 (60 %) of the latent sequences but only 10 % of subtype B sequences.

To investigate this further, we cloned this and other mutations affecting splicing into the HIV proviral clone NL4-3 and an NL4-3 based vector expressing GFP in *env*, and examined the replicative capacity of the mutant viruses compared to wild type. We also studied the effect of these mutations on viral RNA splicing patterns and the dynamics of viral gene expression in a primary cell model of latency. Results of our studies will be presented.

**References**

1. Erkelenz S, et al. Balanced splicing at the Tat-specific HIV-1 3′ss A3 is critical for HIV-1 replication. Retrovirology. 2015;12(1):29.
2. Ho Y-C, et al. Replication-competent noninduced proviruses in the latent reservoir increase barrier to HIV-1 cure. Cell. 2013;155(3):540–51.

#### P21 Culture-to-Ct: A fast and direct RT-qPCR HIV gene reactivation screening method using primary T cell culture

##### Valentin Le Douce, Ann Marie McCartin, Virginie Gautier

###### University College Dublin, School of Medicine, College of Health and Agricultural Science, Centre for Research in Infectious Diseases, Dublin, Ireland

**Correspondence:** Valentin Le Douce

*Retrovirology* 2016, **13(Suppl 1)**: P21

**Background:** To accelerate HIV cure research and the identification of new Latency Reversing Agents (LRAS), we need to develop new tools with high-screening capacity. Here we report a new specific and highly sensitive RT-qPCR method to measure variation in HIV gene expression directly from a mixture of latently infected primary T cells and supernatant bypassing the need of cumbersome cell isolation, and RNA extraction and purification steps.

**Specificity and versatility:** Our method is based on a one-step RT-PCR followed by a Taqman probe qPCR (RT-PCR/qPCR) strategy targeting either regions flanking Tat intron or the 3′ polyadenylated LTR of the viral RNA genome. Our assay was optimal to accurately measure changes in the production of total polyadenylated and/or multiply-spliced viral RNAs with high specificity and reproducibility.

**Medium/high-throughput format:** Primary T cell model of HIV-1 latency adapted from the Planelles Model were cultured in a 96-well-plate format with 100,000 cells per well and treated with LRAs or activators. With this layout we successfully achieved quantification of HIV-1 RNAs directly from 10 µl (10,000 cells) of unprocessed mixture cell/supernatants or cell free supernatant with PCR efficiency steadily and strictly above 1.8.

**Sensitivity:** The sensitivity of our assay can be adjusted to low pro-viral load by increasing the number of PCR cycles during the first RT-PCR step. We successfully detected LRA-mediated reactivation of HIV gene expression with as little as 1 % of HIV infected cells.

Our assay facilitates medium/high throughput LRA screening in a 96-well plate format, using as little as 10,000 primary T cells latently infected with HIV per point, and bypassing costly and laborious RNA extraction/purification while capitalising on the fast, sensitive and reproducible RT-PCR/qPCR quantification system.

#### P22 A novel approach to define populations of early silenced proviruses

##### Dalibor Miklik, Filip Senigl, Jiri Hejnar

###### Institute of Molecular Genetics of the ASCR, Laboratory of Viral and Cellular Genetics, Prague, Czech Republic

**Correspondence:** Dalibor Miklik

*Retrovirology* 2016, **13(Suppl 1)**: P22

**Background:** Integration of retroviral genome into the host DNA is a key step of retroviral replication cycle ensuring efficient expression of retroviral genes. Proviral expression is influenced by numerous factors including epigenetic environment at the site of proviral integration being one of the most important factors affecting proviral transcriptional activity. Transcription of the integrated provirus is frequently silenced resulting in the establishment of latently infected cells. The silencing of proviral transcription can be classified as early and late according to the timing when the silencing occurs. Here we present novel approach designed to study the population of early silenced proviruses.

**Methods:** Our approach for detection of early silenced proviruses consists of reporter cell line and specially designed retroviral vector. Both, the reporter cell line and the vector encode marker for retroviral expressional activity. While vector-encoded marker refers to actual expressional activity of the provirus, cell line-encoded marker is activated early after start of the proviral expression and can be detected independently of current proviral expressional activity. We refer to the single cell line-encoded marker-positive cells as early silenced provirus-bearing cells. Nondefective, expression-competent proviruses can be also detected in the double negative population corresponding to immediately silenced proviruses. Using this approach, we define, isolate and study populations of early silenced proviruses of vectors derived from diverse retroviruses including ASLV, MLV and HIV-1. Defining

**Conclusion:** This novel assay provides a tool for quantification of immediately, early and late silenced proviruses. Further analysis of these distinct populations of integrated proviruses offers a valuable dataset for detailed analysis of factors involved in these various means of provirus transcriptional silencing.

### Topic 4: RNA trafficking & packaging

#### P23 Functional analysis of the structure and conformation of HIV-1 genome RNA DIS

##### Jun-ichi Sakuragi^1^, Sayuri Sakuragi^1^, Masaru Yokoyama^2^, Tatsuo Shioda^1^, Hironori Sato^2^

###### ^1^RIMD, Osaka University, Dept. Viral Infections, Osaka, Japan; ^2^National Institute of Infectious Diseases, Pathogen Genomics Center, Tokyo, Japan

**Correspondence:** Jun-ichi Sakuragi

*Retrovirology* 2016, **13(Suppl 1)**: P23

**Background:** The dimer initiation site/dimer linkage sequence (DIS/DLS) region of HIV is located on the 5′ end of the viral genome and suggested to form complex secondary/tertiary structures. Within this structure, stem-loop 1 (SL1) is believed to be most important and an essential key to dimerization, since the sequence and predicted secondary structure of SL1 are highly stable and conserved among various virus subtypes. In particular, a 6-base palindromic sequence is always present at the hairpin loop of SL1 and the formation of kissing-loop structure at this position between the two strands of genomic RNA is suggested to trigger dimerization. Although the higher-order structure model of SL1 is well accepted and perhaps even undoubted lately, there could be stillroom for consideration to depict the functional SL1 structure while in vivo (in virion or cell).

**Results:** In this study, we performed several analyses to identify the nucleotides and/or basepairing within SL1 which are necessary for HIV-1 genome dimerization, encapsidation, recombination and infectivity. We unexpectedly found that some nucleotides that are believed to contribute the formation of the stem do not impact dimerization or infectivity. On the other hand, we found that one G–C basepair involved in stem formation may serve as an alternative dimer interactive site (DIntS). We also report on our further investigation of the roles of the palindromic sequences on viral replication. Collectively, we aim to assemble a more-comprehensive functional map of SL1 on the HIV-1 viral life cycle.

**Conclusions:** We discovered several possibilities for a novel structure of SL1 in HIV-1 DLS. The newly proposed structure model suggested that the hairpin loop of SL1 appeared larger, and genome dimerization process might consist of more complicated mechanism than previously understood. Further investigations would be still required to fully understand the genome packaging and dimerization of HIV.

#### P24 Regulation of foamy viral *env* splicing controls *gag* and *pol* expression

##### Jochen Bodem^1^, Rebecca Moschall^1^, Sarah Denk^1^, Steffen Erkelenz^2^, Christian Schenk^1^, Heiner Schaal^2^

###### ^1^University Würzburg, Institute for Virology and Immunbiology, Würzburg, Germany; ^2^Heinrich-Heine-University Düsseldorf, Institute for Virology and Immunbiology, Würzburg, Germany

**Correspondence:** Jochen Bodem

*Retrovirology* 2016, **13(Suppl 1)**: P24

The foamy viral genome encodes four central purine-rich elements localized in integrase encoding sequence of *pol*. Previously, we have shown that the first two of these RNA elements are required for protease dimerization and activation. The third element was described to be essential for *gag* expression indicating that it might serve as RNA export element. Here, we show that this element is not responsible for the export of genomic and *pol* RNAs, but encodes a splice enhancer element, which acts negatively on *env* splicing. Thus, recruitment of a positive splice factor surprisingly results in intron retention, whereas the deletion of this element promotes complete splicing of almost all LTR derived transcripts to an *env* 3′ splice site localized in *pol*. We show that this inhibition of splicing is achieved by overlapping splice enhancer and branch point sequences, which influence branch point recognition and SF1/mBBP binding. Separation of branch point and the splice enhancer sequences by insertion of a few nucleotides restored splicing to the *env* 3′ splice site similar to the deletion of the enhancer element in the wild-type context. In summary, we provide evidence that splice enhancer might act negatively on splicing by blocking the recognition of essential splice signals.

### Topic 5: Assembly & release

#### P25 Transfer of HTLV-1 p8 to target T cells depends on VASP: a novel interaction partner of p8

##### Norbert Donhauser^1^, Ellen Socher^2^, Sebastian Millen^1^, Heinrich Sticht^2^, Andrea K. Thoma-Kress^1^

###### ^1^Institute of Clinical and Molecular Virology, Friedrich-Alexander-Universität Erlangen-Nuremberg, Erlangen, Germany; ^2^Institute of Biochemistry, Division of Bioinformatics, Erlangen, Germany

**Correspondence:** Andrea K. Thoma-Kress

*Retrovirology* 2016, **13(Suppl 1)**: P25

The Human T-cell leukemia virus type 1 (HTLV-1)-encoded accessory protein p8 induces cellular conduits, which are thought to facilitate transfer of p8 to target cells and virus transmission. Host factors interacting with p8, enhancing p8 transfer, and HTLV-1 transmission are unknown. Here, we report that vasodilator-stimulated phosphoprotein (VASP) is a novel interaction partner of p8. VASP contains an Ena/VASP homology 1 (EVH1) domain that targets the protein to focal adhesions. Functionally, VASP prevents elongating actin-filaments from capping thereby promoting filament elongation. Bioinformatics identified a proline-rich sequence stretch in p8, which may interact with EVH1 domains of host proteins like VASP. The predictions were verified by co-immunoprecipitation experiments. VASP and p8 co-precipitated not only after co-expression in 293T and Jurkat T-cells, but also after mixing of lysates expressing VASP and p8 individually. Co-precipitation could be blocked by peptides mimicking the predicted EVH1 binding motif in p8, but not by a control peptide, which covers also a proline-rich sequence stretch, but which was not predicted as an EVH1-binding motif. Mutational studies revealed that the EVH1-domain of VASP is necessary, but not sufficient for the interaction with p8. Beyond, deletion of the G- and F-actin binding domains within VASP significantly diminished co-precipitation of p8. Immunofluorescence analysis identified areas of partial co-localization of VASP with p8 at the plasma membrane and in protrusive structures between T-cells. Co-culture experiments revealed that p8 is transferred between Jurkat T-cells via VASP-containing conduits. Repression of both endogenous and overexpressed VASP by small hairpin RNAs strongly reduced p8 transfer. Taken together, we identified VASP as a novel interaction partner of p8, which is important for transfer of p8 to target cells and could thus contribute to the formation of cellular conduits to promote HTLV-1 transmission.

#### P26 COL4A1 and COL4A2 are novel HTLV-1 tax targets with a putative role in virus transmission

##### Christine Gross^1^, Sebastian Millen^1^, Melanie Mann^1,2^, Klaus Überla^1^, Andrea K. Thoma-Kress^1^

###### ^1^Institute of Clinical and Molecular Virology, Friedrich-Alexander-University Erlangen-Nuremberg, Erlangen, Germany; ^2^The Francis Crick Institute, London, Great Britain

**Correspondence:** Sebastian Millen

*Retrovirology* 2016, **13(Suppl 1)**: P26

Human T-cell leukemia virus type 1 (HTLV-1) infects CD4^+^ T-cells preferentially via cell-to-cell transmission requiring reorganization of the cytoskeleton as well as expression of the viral key-player and oncoprotein Tax. Collagens are not only part of the basal membrane but also represent an important component of the viral biofilm, which depicts a fundamental route of transmission. Thus, we asked, (1) if and what type of collagens are expressed in HTLV-1-infected T-cells and (2) whether these collagens are (up)regulated by Tax. Making use of microarrays, RT-PCR, qPCR, western Blot, luciferase assays and immunofluorescence analysis, we found that Col4α1 (COL4A1) and Col4α2 (COL4A2) are the only collagens to be upregulated in the presence of Tax in three independent systems. Being transcribed from a shared and common bidirectional promoter, *COL4A1* and *COL4A2* are translated into individual α-chains that finally assemble to heterotrimers (α1α1α2), underlying several posttranslational modification steps. Both *COL4A1* and *COL4A2* transcripts and Collagen4 protein (COL4) can be shown to be upregulated in HTLV-1-positive T-cell lines. However, COL4 protein was only detectable in HTLV-1-infected cell lines that produce virions, suggesting that COL4 either contributes to viral transmission or plays its major role upon cellular transformation. Mechanistically, we found that Tax induces *COL4A1* and *COL4A2*: (1) Repression of Tax in a Tax-transformed T-cell line led to a significant reduction of *COL4A1/A2*. (2) Overexpression of Tax in Jurkat T-cells led to an induction of *COL4A1/A2*. This finding was further supported by luciferase-based promoter studies indicating that Tax activates the COL4A1 and, to a less extent, the COL4A2 promoter. (3) *COL4A1/A2* increased in a time-dependent manner in a Tax-inducible T-cell line. Results obtained from Tax-mutants suggest that both Tax-induced CREB and NF-κB signaling are crucial for Tax-mediated transcriptional induction of *COL4A1/A2*. Though, merely the NF-κB pathway seems to play a predominant role in stabilizing and maintaining Tax protein itself whereas CREB-signaling appears to be dispensable. Taken together, we identified *COL4A1* and *COL4A2* as novel cellular targets of Tax, potentially leading to an improved understanding of HTLV-1 transmission.

#### P27 The C terminus of foamy virus gag protein is required for particle formation, and virus budding: starting assembly at the C terminus?

##### Guochao Wei^1^, Matthew J. Betts^2^, Yang Liu^1^, Timo Kehl^1,2^, Robert B. Russell^2^, Martin Löchelt^1^

###### ^1^DKFZ, F020, Heidelberg, Germany; ^2^University of Heidelberg, Heidelberg, Germany

**Correspondence:** Martin Löchelt

*Retrovirology* 2016, **13(Suppl 1)**: P27

Particle formation and budding from infected cells are fundamentally different in foamy viruses (FVs) compared to the orthoretroviruses. Unlike in other retrovirus, Env is required to interact with the Gag N terminus for particle release and Gag in capsids assembled at the microtubule organizing center (MTOC) and in released particles is not cleaved in the canonical MA, CA and NC proteins.

While Primate Foamy Virus (PFV) Gag has been characterised with respect to particle formation, genome packaging and budding, central and C-terminal sequences of non-primate FV Gag are less characterised and their contribution to capsid formation and release has so far not been analysed. We have recently shown that the feline FV (FFV) Gag N terminus is required for MTOC targeting, capsid assembly, RNA and Pol packaging and subsequent Gag processing at a C-terminal site. To study the importance of the Gag C-terminus for assembly and budding, C-terminally truncated FFV Gag variants were analysed. With the exception of a 7.5 kDa C-terminal domain, most of Gag is necessary for capsid assembly and Env-dependent particle release. Sucrose gradient analyses of cytosolic extracts of HEK293T cells transfected with wt and truncated Gag constructs allowed identification of different assembly and maturation intermediates like soluble Gag, capsomeres and capsids.

The intactness of the highly conserved FV Gag motif QPQRYG was essential for capsid assembly in truncated Gag expression constructs while amino acid replacement mutagenesis of QPQ and RYG of this motif plus conserved upstream Y and R residues in the FFV provirus showed wt capsid formation and budding but strongly reduced particle infectivity. We assume that gross deletions in the C-terminal nucleic-binding domain of Gag interfere with unspecific RNA binding required for capsid formation which is in other retroviruses orchestrated by membrane targeting of Gag. In contrast, the more subtle changes in the Gag QPQRYG motif and the adjacent chromatin binding site may for instance interfere only with genome encapsidation, reverse transcription and/or integration. Studies are underway to identify and characterise the underlying mechanisms.

#### P28 Generation of an antigen-capture ELISA and analysis of Rec and Staufen-1 effects on HERV-K(HML-2) virus particle production

##### Oliver Hohn, Saeed Mostafa, Kirsten Hanke, Stephen Norley, Norbert Bannert

###### Robert Koch Institute, FG18 HIV and other Retroviruses, Berlin, Germany

**Correspondence:** Oliver Hohn

*Retrovirology* 2016, **13(Suppl 1)**: P28

**Question:** The youngest family of human endogenous retroviruses, HERV-K(HML-2), is biologically active and is able to produce viral particles, at least under certain circumstances such as malignant diseases. Using a reconstituted HERV-K(HML-2) sequence (oriHERV-K113), we recently showed that human Staufen-1 protein is an interaction partner of the accessory Rec protein. Similar to the situation for HIV-1 Rev, the action of HERV-K(HML-2) Rec, together with host cell factors, is essential for virus particle production.

**Methods:** The capsid protein (p27-CA) domain from the oriHERV-K113 *gag* sequence was expressed in *E.coli* and used to immunize rabbits. A monoclonal antibody to HERV-K113 Gag (Boller et al. 2008) was used as the basis for a HERV-K(HML-2) p27-CA antigen-capture ELISA (AC-ELISA). HEK 293T cells were transfected with molecular clones of various exogenous retroviruses and of oriHERV-K113 and virus lysates analysed using the p27-CA AC-ELISA. Mutations preventing Rec splicing were introduced into the full length molecular clone of oriHERV-K113 by site-directed mutagenesis to generate oriHERV-K113Drec.

**Results:** The new AC-ELISA was shown to be sensitive and specific for the p27-CA of HERV-K(HML-2). Deletion of Rec resulted in drastically impaired virus production that could be restored by providing Rec *in trans*. Moreover, addition of Staufen-1 (a previously identified cellular interaction partner of Rec) together with Rec *in trans* resulted in even higher virus levels by oriHERV-K113Drec than those with oriHERV-K113 alone. Whereas co-transfection with a full-length HIV-1 molecular clone had no significant influence on oriHERV-K113 particle production, HIV-1 Rev alone *in trans* could rescue particle expression by oriHERV-K113Drec.

**Conclusions:** The HERV-K(HML-2) p27-CA AC-ELISA is a useful tool for monitoring virus production in vitro. Deletion of Rec significantly inhibits viral particle production but this can be overcome by Rec and also by HIV-1 Rev *in trans*. However, there is also evidence for an additional, Rec-independent mechanism of HERV-K(HML-2) enhancement by Staufen-1.

**Reference**

1. Boller et al., J Gen Virol. 2008;89:567–72.

#### P29 Antagonism of BST-2/tetherin is a conserved function of primary HIV-2 Env glycoproteins

##### Chia-Yen Chen^1^, Masashi Shingai^1^, Pedro Borrego^2^, Nuno Taveira^2^, Klaus Strebel^1^

###### ^1^NIH, NIAID, Bethesda, Great Britain; ^2^University of Lisbon, Faculty of Pharmacy, Lisbon, Portugal

**Correspondence:** Klaus Strebel

*Retrovirology* 2016, **13(Suppl 1)**: P29

Although HIV-2 does not encode a *vpu* gene, the ability to antagonize BST-2 is conserved in some HIV-2 isolates where it is controlled by the Env glycoprotein. We previously reported that a single amino acid difference between the lab-adapted ROD10 and ROD14 Envs controlled the Vpu-like activity. In this study we investigated how conserved the Vpu-like activity is in primary HIV-2 isolates. We found that almost half of the 35 tested primary HIV-2 Env isolates obtained from 8 different patients exhibited Vpu-like activity. Interestingly, each HIV-2 patient harbored a mixed population of viruses with or without Vpu-like activity. Vpu-like activity and envelope function varied significantly among Env isolates. However, there was no direct correlation between these two Env functions suggesting they evolve independently. In comparing the Env sequences from one HIV-2 patient we found that similar to the ROD10/ROD14 Envs a single amino acid change (T568I) in the ectodomain of the TM subunit was sufficient to confer Vpu-like activity to an inactive Env variant. Surprisingly, however, absence of Vpu-like activity was not correlated with absence of BST-2 interaction. Taken together, our data suggest that maintaining the ability to antagonize BST-2 is of functional relevance not only to HIV-1 but to HIV-2 as well. Our data show that as with Vpu, binding to BST-2 is important but not sufficient for antagonism. Finally, as observed before, the Vpu-like activity in HIV-2 Env can be controlled by very subtle changes in the TM subunit.

#### P30 Mutations in the packaging signal region of the HIV-1 genome cause a late domain mutant phenotype

##### Chris Hellmund, Bo Meng, Andrew Lever

###### University of Cambridge, Department of Medicine, Cambridge, Great Britain

**Correspondence:** Chris Hellmund

*Retrovirology* 2016, **13(Suppl 1)**: P30

During and after budding of the HIV-1 virion, the structural polyprotein Gag undergoes cleavage by the viral protease in a precisely coordinated manner, leading to structural maturation of the virus core, enabling the particle to become infectious. Mutation of an RNA stem-loop (SL1) in the 5′ untranslated region of the virus genome, which is involved in genome dimerization and packaging, causes a delay in the final step of Gag proteolytic processing, preventing correct particle maturation and severely reducing infectivity. To investigate whether dimerization is a requirement for correct Gag processing, as it appears to be in HIV-2, we analysed Gag processing in three SL1 mutants with minimal to severe dimerization defects, and found no correlation between the phenotypes. We found that mutation of a neighbouring structure (SL3), which is the key determinant of genome packaging, also exhibits a delay in Gag processing, suggesting that proper Gag processing is dependent on efficient packaging rather than genome dimerization. Impaired Gag processing is also a characteristic phenotype of late domain mutants, where deletions of conserved motifs in the p6 domain of Gag disrupt interactions with members of the host ESCRT machinery (TSG101 and ALIX) which are hijacked by the virus for budding. We observed that both SL1 and SL3 mutations cause budding defects, suggesting that genomic RNA packaging and virus budding are linked. It has been demonstrated that ALIX can bind to the nucleocapsid domain of Gag in an RNA-dependent manner. Using an assay where ALIX is overexpressed to rescue inefficient budding of a late domain mutant, we found that introduction of an SL1 mutation alongside the late domain mutation reduces the ability of ALIX to rescue the budding defect. These data are consistent with a model whereby genomic RNA packaging is important for efficient maturation and budding of virus particles.

#### P31 p6 regulates membrane association of HIV-1 gag

##### Melanie Friedrich^1^, Friedrich Hahn^1^, Christian Setz^1^, Pia Rauch^1^, Kirsten Fraedrich^1^, Alina Matthaei^1^, Petra Henklein^2^, Maximilian Traxdorf^3^, Torgils Fossen^4^, Ulrich Schubert^1^

###### ^1^Institute of Virology, Friedrich-Alexander University Erlangen-Nuremberg, Erlangen, Germany; ^2^Institute of Biochemistry, Charité Medical University Berlin, Berlin, Germany; ^3^Department of Otorhinolaryngology, Head and Neck Surgery, Friedrich-Alexander University Erlangen-Nuremberg, Erlangen, Germany; ^4^Department of Chemistry and Center for Pharmacy, University of Bergen, Bergen, Norway

**Correspondence:** Melanie Friedrich

*Retrovirology* 2016, **13(Suppl 1)**: P31

As the C-terminal part of the Pr55 Gag polyprotein, the HIV-1 p6 protein regulates the final abscission step of nascent virions from the cell surface by the action of its two late (L-) domains which recruit Tsg101 and ALIX, components of the endosomal sorting complex required for transport (ESCRT). Besides its essential role in virus budding there is increasing evidence that the 52 aa p6, in addition to the canonical membrane targeting signals in matrix, governs interaction of Gag with the cytoplasmic face of the plasma membrane. Mutation of the highly conserved Ser-40 to Phe (S40F) in p6, a mutation frequently occurring in patients with treatment failure, disturbs CA-SP1 processing, reduces infectivity and replication capacity, while virus release remains unaffected. Others found that S40F causes formation of filopodia-like structures enabling cell to cell transmission of the virus.

Furthermore, S40F-mutation augments K48-linked polyubiquitination of Gag, its entry into the ubiquitin proteasome system (UPS) and into the MHC-I antigen pathway. Phe-40, together with Tyr-36, causes the formation of a hydrophobic patch within the C-terminal α-helix of p6, providing a molecular rationale for the enhanced membrane association of S40F Gag, as shown by membrane flotation assays, NMR spectroscopy, and surface plasmon resonance studies.

Furthermore, we found that mutation of the 7 highly conserved glutamic acids within p6 to alanine (E0A), causes defective virus budding and, like the S40F mutant, leads to an enhanced polyubiquitination and subsequent entry of Gag into the UPS corresponding to an increased MHC-I antigen presentation of Gag derived epitopes. In addition, like for the S40F mutant, the CA-SP1 processing of the E0A mutant is impaired, also resulting in loss of infectivity and impaired virus replication.

The cumulative data support a model in which p6, either by hydrophobic (S40F) or electrostatic (E0A) interactions with the plasma membrane, acts, in addition to matrix, as a membrane targeting domain of Gag. The extended exposure to a membrane-resident E3-ligase complex, comprising, among others, CCDC8 and Cul7, might augment the polyubiquitination, entry into the UPS, and thus the immunogenicity of Gag. However, the localization and biological function of p6 after maturation of Gag still remains enigmatic.

### Topic 6: Pathogenesis & evolution

#### P32 Molecular and structural basis of protein evolution during viral adaptation

##### Aya Khwaja, Meytal Galilee, Akram Alian

###### Technion, Biology, Haifa, Israel

**Correspondence:** Akram Alian

*Retrovirology* 2016, **13(Suppl 1)**: P32

Random mutations, which afford virus evasion of intrinsic host and therapeutically administered opposition, rarely provide direct survival adaptations and more often have deleterious effects by directly perturbing interfaces central to *intrinsic* protein folding or *extrinsic* interactions essential for crafting viral protein assemblies. Mutant proteins may regain functional stability and folding by employing either, or both, an *intrinsic* mechanism, through the accumulation of *coevolved mutations* that induce compensatory conformational changes, or by an *extrinsic* mode via the interaction with molecular chaperoning proteins promoting the acquisition of the functional folded state.

Cross-family differences and interspecies subtleties can aid highlighting resistance-mechanisms accessible by mutational adaptation of challenged viruses. Exploring how related viruses coevolved in their natural environment can, therefore, highlight crucially conserved patterns and uncover conceivable latent escape routes potentially accessible to emergent viral strains. We determined the structure of capsid from feline immunodeficiency virus (FIV) and revealed that the functional structure is preserved through spatial correlations of coevolved substitutions, which when otherwise uncoupled and individually substituted into HIV-1 capsid impairs virus infectivity. This example illustrates an *intrinsic mechanism* during viral adaptation. The *extrinsic mechanism* of protein adaptation will be illustrated by discussing the structural and molecular basis of lentiviral Vif flexibility in exploiting various chaperoning cofactors during evasion of APOBEC3 cellular-restriction.

The ability to circumvent deleterious effects of mutations affords viruses an important survival advantage by exploiting alternative, but functionally equivalent, patterns when the default ones are blocked.

#### P33 HIV-1 enhancement and neutralisation by soluble gp120 and its role for the selection of the R5-tropic “best fit”

##### Birco Schwalbe, Heiko Hauser, Michael Schreiber

###### Bernhard Nocht Institute for Tropical Medicine, Virology, Hamburg, Germany

**Correspondence:** Michael Schreiber

*Retrovirology* 2016, **13(Suppl 1)**: P33

**Question:** HIV-1 entry into cells is linked to the ability of gp120 to use at least one of the coreceptors CCR5 (R5) or CXCR4 (X4). However, a mixed viral quasispecies is present in natural infection competing for permissive cells. During progression of the disease the shift from R5-to-X4 is explained by selectivity of cell reservoirs and selective immune responses but the direct impact of a single virus on the suppression or enhancement of other X4- or R5-viruses is not completely elucidated. Cells, productively infected by HIV-1 release viral particles as well as the gp120 envelope into body fluids. Thus, soluble gp120 (sgp120) will compete for receptor binding with other virus variants.

**Fig. 12:**
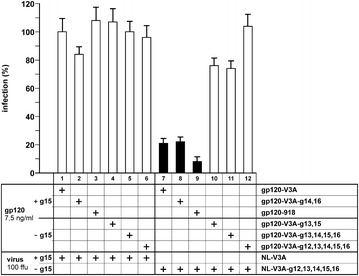
Suppression of viral infection by +g15 R5-topic viruses (subtype A V3 loop). Gp120 of the +g15 type was neutralizing the −g15 mutant (NL-V3A-g12, 13, 14, 15, 16, *black bars*). Such an effect was not observed using the −g15 gp120 on the +g15 virus. +g15 = presence of the *N*-glycan within the V3 loop; −g15 = lack of the V3 loop *N*-glycan, V3A = subtype A V3 loop, gp120–918 = a subtype B R5-tropic gp120 used for comparison

**Methods:** To study viral infection in the presence of soluble gp120 (sgp120) we have constructed pairs of R5- and X4-tropic viruses with high and low infectivity and the corresponding sgp120. We preferentially mutated *N*-glycosylation sites in and around the gp120 V3 loop and introduced arginine residues close to the *N*-glycosylation site present in the V3 loop (Fig. 12).

**Results:** For X4-tropic virus, mutated *N*-glycosylation sites lead to enhanced infectivity and for R5-tropic viruses, their lack leads to loss of R5-specific infectivity. We demonstrated, that R5-tropic virus was suppressed by heterologous sgp120 derived from R5-viruses of higher infectivity. Thus, the R5-tropic “best-fit” can block infection of other R5-tropic rivals. In X4-specific infection we also demonstrated neutralisation of viruses by sgp120 with the same coreceptor tropism. Here, viruses of a higher efficiency were able to enhance their X4-tropic rivals. Another support of infection was observed for R5-tropic infection in the presence of sgp120 derived from X4-virus. R5-tropic infection was strongly enhanced by X4-sgp120. This cross-enhancement was linked to the presence of heparan sulphate proteoglycans (HSPG) on the surface of CCR5+ target cells. Effects of R5-virus enhancement and suppression were studied for HIV-1 subtype B, A and C envelopes in the background of the NL4-3 laboratory strain. We demonstrated that the *N*-glycan g15 within the V3 loop played a role in selection of X4- and R5-tropic viruses and arginine amino acids surounding the g15 *N*-glycosylation site.

**Conclusion:** We propose, that soluble X4- and R5-gp120 is supporting the R5-tropic viral “best fit”.

#### P34 An insertion of seven amino acids in the Env cytoplasmic tail of Human Immunodeficiency Virus type 2 (HIV-2) selected during disease progression enhances viral replication

##### François Dufrasne, Mara Lucchetti, Patrick Goubau, Jean Ruelle

###### Université catholique de Louvain, AIDS Reference Laboratory, ARL, Bruxelles, Belgium

**Correspondence:** François Dufrasne

*Retrovirology* 2016, **13(Suppl 1)**: P34

**Question:** The cytoplasmic tail (CT) of the transmembrane envelope glycoprotein (gpTM) of HIV-2 includes amino acids (aa) sequences similar to lentiviral lytic peptides (LLP) described in other lentiviruses [1, 2]. Within the putative LLP-2 region, we previously observed insertions of 3 or 7 aa in sequences deduced from plasma viral RNA of symptomatic HIV-2 infected individuals [3]. Based on these observations, we reproduced the insertions in a molecular clone to assess their impact on replicatve fitness and cell death.

**Methods:** Using a molecular clone of the HIV-2 ROD reference strain, site-directed mutagenesis experiments allowed the generation of plasmids with the insertion I_787_ *SQS* or I_787_ *FRSLQRA* in Env. After transfection in HEK293T cells, the resulting viral particles were used to infect H9 cells. Viral release was quantified by RT-qPCR at three and six days post-infection. Cell viability was assessed with the percentage of living cells using a CASY cell counter.

**Fig. 13:**
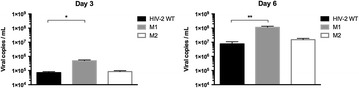
Absolute quantification of viral release from infected H9 cells at 3 and 6 days post-infection [n = 3 independent experiments, *p* value <0.05 (*) and <0.01 (**)]

**Fig. 14:**
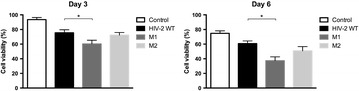
Percentage of living cells after 3 and 6 days post-infection (n = 3 independent experiments)

**Results:** Compared to the control wild-type ROD virus, the clone with a 7 aa insertion in the LLP-2 region (M1) enhanced viral release ten times (Fig. 13). Cell viability was 20 % more impaired compared to the wild type (Fig. 14). The effect of the 3 aa insertion (M2) was milder, with a non-significant trend to enhance viral release and cell death compared to the wild-type.

**Conclusions:** A 7 aa insertion including positively charged aa in the putative LLP-2 enhances viral replication and cell death in vitro. The insertions in the Env CT observed in vivo in samples from disease progressors may therefore be involved in the higher viral load observed in these individuals. Further investigations are needed to assess the effect of the lytic peptide on cell death. This study may open the way to the development of laboratory diagnostic tools related to disease progression.

**References**

1. Santos da Silva E, Mulinge M, Perez Bercoff D. The frantic play of the concealed HIV envelope cytoplasmic tail. Retrovirology. 2013;10:54.
2. Postler TS, Desrosiers RC. The tale of the long tail: the cytoplasmic domain of HIV-1 gp41. J Virol. 2013;87:2–15.
3. Bakouche N, Vandenbroucke AT, Goubau P, Ruelle J. Study of the HIV-2 Env cytoplasmic tail variability and its impact on Tat, Rev and Nef. PLoS One. 2013;8:e79129.

#### P35 Cell-associated HIV-1 unspliced to multiply spliced RNA ratio at 12 weeks ART correlates with markers of immune activation and apoptosis and predicts the CD4^+^ T-cell count at 96 weeks ART

##### Mirte Scherpenisse, Ben Berkhout, Alexander Pasternak

###### Academic Medical Center of the University of Amsterdam, Laboratory of Experimental Virology, Amsterdam, Netherlands

**Correspondence:** Alexander Pasternak

*Retrovirology* 2016, **13(Suppl 1)**: P35

Combination antiretroviral therapy (ART) suppresses HIV-1 replication and improves immune function. Considerable proportion of HIV-infected individuals receiving ART fail to increase CD4^+^ T-cell counts sufficiently. Incomplete restoration of CD4^+^ T-cell count during virologically successful ART is a major predictor of morbidity and mortality. For better understanding of HIV-1 pathogenesis and improved design of curative strategies, it is important to determine whether the degree of HIV-1 persistence, measured early on ART, can predict subsequent immunological response to the long-term therapy and whether HIV-1 persistence correlates with host biomarkers of immune dysfunction. Viral (total and episomal HIV-1 DNA, unspliced and multiply spliced [total and *tat/rev*] cell-associated HIV-1 RNA) and host (CD4^+^ and CD8^+^ T-cell activation, proliferation, senescence, apoptosis, exhaustion, thymic migration, and CD4^+^ and CD8^+^ T-cell subsets) biomarkers were longitudinally measured in a cohort of 28 HIV-infected patients at 0, 12, 24, 48, and 96 weeks of virologically suppressive ART. No baseline HIV-1 marker was predictive of CD4^+^ T-cell count at 96 weeks of ART. However, at 12 weeks of ART, cell-associated HIV-1 unspliced to multiply spliced-total (US/MS) RNA ratio, despite not being associated with baseline CD4^+^ T-cell count, strongly negatively correlated with both absolute CD4^+^ T-cell count at 96 weeks of ART (*rho* = −0.56, *P* = 0.004) and with relative increase in CD4^+^ T-cell count between baseline and 96 weeks of ART (*rho* = −0.55, *P* = 0.004). Moreover, US/MS RNA ratio at 12 weeks ART strongly positively correlated with markers of CD4^+^ T-cell activation (CD4^+^/CD38^+^/HLA-DR^+^: *rho* = 0.63, *P* = 0.001) and apoptosis (CD4^+^/Annexin-V^+^/FAS^+^: *rho* = 0.59, *P* = 0.002). Because HIV life cycle involves a temporal shift from the production of multiply spliced to the production of unspliced RNA species, higher US/MS RNA ratio in a patient might reflect the higher frequency of HIV-infected cells in the later stages of viral life cycle, which is characterised by expression of viral proteins and presentation of antigens. Such cells could exert pressure on the host immune system, causing persistent immune activation and apoptosis and contributing to poor immunological response to ART.

#### P36 Faster progression in non-B subtype HIV-1-infected patients than Korean subclade of subtype B is accompanied by higher variation and no induction of gross deletion in non-B *nef* gene by Korean red ginseng treatment

##### Young-Keol Cho, Jungeun Kim, Daeun Jeong

###### University of Ulsan College of Med., Microbiology, Seoul, South Korea

**Correspondence:** Young-Keol Cho

*Retrovirology* 2016, **13(Suppl 1)**: P36

**Question:** HIV-1 infections by subtype B account for about 12 % of infections worldwide. Evidence is rapidly accumulating that there is a significant difference in natural progression and response to HAART among subtypes. Gross deletion in the HIV *nef* gene (gΔ*nef*) is associated with slow progression in subtype B-infected patients (B). To date, there is no such data in patients infected with non-B subtypes (non-B).

**Fig. 15:**
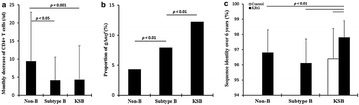
There were significant differences in monthly decrease of CD4, proportion of gΔ*nef*, and rate of variation among 3 subtypes

**Methods:** To investigate whether there is a difference in natural progression, response to Korean red ginseng (KRG), and proportion of gΔ*nef* among Korean subclade of subtype B (KSB) (n = 103), subtype B (n = 39), and non-B (n = 124) infected patients in the absence of HAART, we compared rate of decrease of CD4+ T cells (CD4) and the proportion of gΔ*nef* and also with control. We amplified *nef* gene by nested-PCR and determined 4508 *nef* sequences (Fig. 15).

**Results:** Monthly decrease of CD4 was significantly faster in non-B (9.4 ± 14/μL) than 4.1–4.2/μL in B and KSB-infected patients (P < 0.001). Overall proportion of gΔ*nef* was 4.4, 7.9, and 12.2 % in non-B, subtype B, and KSB infected patients, respectively (P < 0.01). One-hundred and sixty-three patients were treated with KRG (3797 ± 4901 g) over 85 ± 63 months and their monthly decrease of CD4 was significantly slower (4 ± 6/μL) than 10 ± 17/μL in 82 KRG-naïve patients (P < 0.001). KRG treatment significantly slowed decrease of CD4 in non-B and KSB patients and significantly increased proportion of gΔ*nef* from 2.1 and 3.7 % at baseline to 12.6 and 17.1 % after 12 months in subtype B and KSB patients, respectively (P < 0.001). In contrast, KRG treatment did not affect the gΔ*nef* in non-B. However, the proportion of sequences with premature stop codon was similar among 3 subtypes (0.5–2.0 %). Sequence identity over 6 years was significantly higher in KSB (97.8 ± 1.1 %) than subtype B (96.1 ± 1.6 %) (P < 0.01) and non-B (96.8 ± 1.5 %) (P < 0.01). The sequence identity 97.8 ± 1.1 % was also higher than 96.4 ± 2.0 % in KSB control (P < 0.01). Taken collectively, 23 long-term slow progressors (LTSPs) was detected in KRG treated patients only (P < 0.01) and its proportion was higher in KSB patients than in non-B patients (P < 0.01).

**Conclusions:** Decrease of CD4 and proportion of gΔ*nef* are affected by subtypes and KRG treatment. Faster decrease of CD4 in non-B patients than in KSB patients is supported by significantly lower gΔ*nef* and higher variation in non-B than KSB.

#### P37 Aberrant expression of ERVWE1 endogenous retrovirus and overexpression of TET dioxygenases are characteristic features of seminoma

##### Katerina Trejbalova^1^, Martina Benesova^1^, Dana Kucerova^1^, Zdenka Vernerova^2^, Rachel Amouroux^3^, Petra Hajkova^3^, Jiri Hejnar^1^

###### ^1^Institute of Molecular Genetics, Dpt. of Viral and Cellular Genetics, Prague 4, Czech Republic; ^2^Third Faculty of Medicine, Charles University in Prague, Department of Pathology, Prague, Czech Republic; ^3^Medical Research Council Clinical Sciences Centre, Imperial College London, London, Great Britain

**Correspondence:** Katerina Trejbalova

*Retrovirology* 2016, **13(Suppl 1)**: P37

**Background:** Germ cell tumors and particularly seminomas reflect the epigenomic features of their parental primordial germ cells, including the genomic DNA hypomethylation and expression of pluripotent cell markers. Because the DNA hypomethylation might be a result of TET dioxygenase activity, we examined expression of TET1–3 enzymes and the level of their product, 5-hydroxymethylcytosine, in a panel of histologically characterised seminomas and non-seminomatous germ cell tumors. Simultaneously, we analysed the expression of ERVWE1 endogenous retrovirus whose spliced form codes for envelope glycoprotein called Syncytin-1. Syncytin-1 has fusogenic ability and its expression is restricted to placenta under physiologic conditions.

**Results:** We found highly increased expression of TET1 dioxygenase in most seminomas and a strong TET1 staining in seminoma cells. Isocitrate dehydrogenase 1 and 2 mutations were not detected suggesting the enzymatic activity of TET1. The levels of 5-methylcytosine and 5-hydroxymethylcytosine in seminomas were found decreased in comparison to non-seminomatous germ cell tumors and healthy testicular tissue. Seminomas further displayed significant increase in both spliced and non-spliced forms of ERVWE1 in comparison to healthy controls. Importantly, the promoter of ERVWE1 in seminomas contained low levels of DNA methylation.

**Conclusions:** We propose TET1 expression as a marker of seminoma and mixed germ cell tumor. Furthermore, the endogenous retrovirus ERVWE1 was consistently overexpressed in seminomas. In contrast to the CpG island methylator phenotype observed in a fraction of tumors of various types, we suggest the anti-methylator phenotype in seminomas is maintained by TET1 demethylation activity.

#### P38 Life history of the oldest lentivirus: characterisation of ELVgv integrations and the ***TRIM5*** selection pattern in dermoptera

##### Daniel Elleder^1^, Tomas Hron^1^, Helena Farkasova^1^, Abinash Padhi^2^, Jan Paces^1^

###### ^1^Institute of Molecular Genetics, Prague, Czech Republic; ^2^University of Maryland, Department of Animal and Avian Sciences, College Park, MA, United States

**Correspondence:** Daniel Elleder

*Retrovirology* 2016, **13(Suppl 1)**: P38

Endogenous retroviruses are genomic elements formed by germline infiltration by originally exogenous viruses. These molecular fossils provide valuable information about the evolution of the retroviral family. Lentiviruses are an extensively studied genus of retroviruses infecting a broad range of mammals. Despite a wealth of information on their modern evolution, little is known about their origins. This is partially due to the scarcity of their endogenous forms. Recently, an endogenous lentivirus, ELVgv, was discovered in the genome of the Malayan colugo (order *Dermoptera*). This represents the oldest lentiviral evidence available and promises to lead to further insights into the history of this genus.

In this study, we analysed ELVgv integrations at several genomic locations in four distinct colugo specimens covering all the extant dermopteran species. We confirmed ELVgv integrations in all the specimens examined, which implies that the virus originated before the dermopteran diversification. Using a locus-specific dermopteran substitution rate, we estimated that the proviral integrations occurred 21–40 million years ago. Using phylogenetic analysis, we estimated that ELVgv invaded an ancestor of today’s *Dermoptera* more than 60 million years ago. We also provide evidence of selective pressure on the *TRIM5* antiviral restriction factor, something usually taken as indirect evidence of past retroviral infection. Interestingly, we show that *TRIM5* was under strong positive selection only in the common dermopteran ancestor and that this period could coincide with ELVgv activity. In summary, we describe the evolutionary history of the oldest known lentiviral lineage and propose its coevolution with the *TRIM5* host restriction factor.

#### P39 Characterisation of a highly divergent endogenous retrovirus in the equine germ line

##### Henan Zhu, Robert Gifford, Pablo Murcia

###### MRC-University of Glasgow Centre for Virus Research, Glasgow, Great Britain

**Correspondence:** Henan Zhu

*Retrovirology* 2016, **13(Suppl 1)**: P39

The general profile of endogenous retroviruses (ERVs) in the domestic horse (*Equus cabalus*) genome has been described, but a thorough Characterisation is lacking. We used an in silico approach based on data mining and phylogenetic analysis to profile equine ERVs in depth. We identified a total of 1384 ERV loci in the horse genome that disclosed a robust phylogenetic relationship to retroviral reverse transcriptase (RT) genes. Through phylogenetic and genomic analyses of these loci we derived an overview of equine ERV diversity. We inferred that there are at least 8 distinct, major lineages of ERVs in the equine germ line, and recovered consensus proviral genome structures for each of these. One highly divergent ERV lineage, which we provisionally refer to as ‘EqERV-u1’, was observed to be unique to the family *Equidae*. We show that EqERV-u1 is intermediate to Alpha- and Betaretroviruses in phylogenetic trees, and identify 46 distinct EqERV-u1 proviruses, including 17 with intact genomes. Interestingly, we observed two distinct genome structures among intact EqERV-u1 copies: a classical (type I) structure in which a *dUTPase* gene is located between the *pro* and *pol* coding domains, and a type II structure—unique to EqERV-u1—in which a *dUTPase* gene occurs upstream of *gag*. We dated the activity of the EqERV-u1 lineage over time using a molecular clock-based approach, revealing that it has been active relatively recently (i.e. within the past 1–5 million years), even though it may have entered the equid germline >18 million years ago. Analysis of published *E.cabalus* transcriptome data revealed that one EqERV-u1 provirus on chromosome 29 is actively transcribed in a tissue-specific manner. This provirus exhibits the unusual type II genome structure.

#### P40 The emergence of pandemic retroviral infection in small ruminants

##### Maria Luisa Carrozza ^1^, Anna-Maria Niewiadomska ^2^, Maurizio Mazzei ^3^, Mounir Abi-Said^4^, Joseph Hughes^5^, Stéphane Hué ^6^, Robert Gifford^5^

###### ^1^Scuola Normale Superiore, Pisa, Italy; ^2^Aaron Diamond AIDS Research Center, New York City, NY, United States; ^3^Università of Pisa, Pisa, Italy; ^4^Lebanese University, Al Fanar, Lebanon; ^5^MRC-University of Glasgow Centre for Virology, Glasgow, Great Britain; ^6^London School of Hygiene and Tropical Medicine, London, Great Britain

**Correspondence:** Robert Gifford

*Retrovirology* 2016, **13(Suppl 1)**: P40

During the 20th century a confluence of socio-epidemiological factors combined to facilitate the emergence of retrovirall pathogens in humans. However, the influence of anthropogenic factors on the emergence of retroviral infections in non-human species has not been evaluated to the same extent. Small ruminant lentiviruses (SRLVs) cause chronic, persistent infections in populations of domestic sheep (*Ovis aries*) and goats (*Capra hircus*) throughout the world. Here, we trace the origins and history the SRLV pandemic. To investigate the ancient history of SRLVs, we performed a serology and DNA sequencing-based investigation of SRLVs diversity in the Fertile Crescent region, where domestication of sheep and goats is thought to have originally occurred. Screening of 886 sheep and goats in Jordan and Lebanon revealed a relatively high prevalence of infection (~21 %) and an elevated level of viral genetic diversity compared to other regions of the world. Furthermore, using sequences obtained via this screen, we show that currently circulating SRLV genotypes reveal evidence of ancient, inter-genotype recombination. These data support the hypothesis that SRLVs disseminated out of Western Asia during the early Neolithic period. However, by using phylogenetic and phylogeographic approaches to analyze SRLV sequences sampled from 600 distinct infections in 30 different countries, and spanning a period of 64 years, we show that pandemic spread of SRLVs did not occur until the 20th century. We integrate the findings of our analysis with historical and epidemiological evidence to propose a geographic sequence and timeline for the emergence of the SRLV pandemic. We identify the Colonial expansion of European nations during the ‘Age of Imperialism’ (~1870–1950), and the associated development of novel agricultural systems, as having played a key role in enabling the global spread of SRLV infection.

#### P41 Near full-length genome (NFLG) characterisation of HIV-1 subtype B identified in South Africa

##### Adetayo Obasa, Graeme Jacobs, Susan Engelbrecht

###### Stellenbosch University, Department of Pathology, Cape Town, South Africa

**Correspondence:** Adetayo Obasa

*Retrovirology* 2016, **13(Suppl 1)**: P41

**Background:** The first reported cases of HIV-1 infection in South Africa occurred in 1982, which was initially spread by MSM. Almost 7 million people are living with HIV-1 infection in South Africa and 2 separate epidemics have been described. The majority of these infections are caused by HIV-1 subtype C, spread through heterosexual contact. The minor subtype B epidemic in South Africa was, in the past, transmitted via MSM. We recently described the detection of new BC URFs circulating in the country. This indicates that both epidemics are still co-circulating in South Africa, but only 6 HIV-1 subtype B NFLGs sequences have been previously characterised.

**Methods:** Ten samples were selected for NFLG amplification. Seven of the samples were obtained from the late 1980s, while the other three samples were from more recent infections. The NFLG amplification was performed using a PCR protocol designed to target two overlapping 5.5 kb fragments. There after samples were sequenced through conventional “Sanger” sequencing and next-generation sequencing (NGS) using the Illumina MiSeq platform. The samples were subtyped using the REGA, COMET, RIP and jpHMM online tools. Multiple sequence alignments were done using MAFFT and then codon aligned. Maximum likelihood phylogenetic trees were constructed in Geneious 9 and MEGA.

**Results:** The six 1980s samples were obtained from MSM in the Western Cape South Africa. The others obtained were from a 16 year old heterosexual teenager in Gauteng, one woman from the Eastern Cape and one woman from the Western Cape. Two of the subtype B NFLG sequences obtained cluster with reference subtype B strains from the 1980s. Three sequences cluster more closely with reference strains from the late 1990s. Another sequence was identified as a unique BC recombinant strain.

**Discussion:** We have detected and characterised HIV-1 subtype B strains circulating in South Africa since the early 1980s to 2000s. This subtype B epidemic crossed over into the heterosexual population, as indicated by infection of both children and women in the different provinces of South Africa of concern is the characterisation of the newly described subtype BC URF strains. We will continue to monitor the HIV-1 subtype B epidemic in the heterosexual population in South Africa.

#### P42 Acquisition of Vpu-mediated tetherin antagonism by an HIV-1 group O strain

##### Katharina Mack^1^, Kathrin Starz^1^, Daniel Sauter^1^, Matthias Geyer^2^, Frederic Bibollet-Ruche^3^, Christina Stürzel^1^, Marie Leoz^4,5^, Jean Christophe Plantier^4,5,6^, Beatrice H. Hahn^3,7^, Frank Kirchhoff^1^

###### ^1^Institute of Molecular Virology, Ulm, Germany; ^2^Max Planck Institute of Molecular Physiology, Department of Physical Biochemistry, Dortmund, Germany; ^3^University of Pennsylvania, Department of Medicine, Philadelphia, PA, United States; ^4^CHU Charles Nicolle, Laboratoire de Virologie, Rouen, France; ^5^Université de Rouen, EA 2656 GRAM, Rouen, France; ^6^CHU Charles Nicolle, Laboratoire associé au Centre National de Référence du VIH, Rouen, France; ^7^University of Pennsylvania, Department of Microbiology, Philadelphia, PA; United States

**Correspondence:** Katharina Mack

*Retrovirology* 2016, **13(Suppl 1)**: P42

The restriction factor tetherin inhibits the release of enveloped viruses and imposes a barrier for efficient spread of HIV in the human population. The direct precursors of HIV-1, SIVcpz and SIVgor, use their Nef protein to antagonize the tetherin orthologue of their respective hosts. Because of a five amino acid deletion in its cytoplasmic tail, human tetherin is resistant to SIV Nef. Overcoming this hurdle may have been a prerequisite for effective spread of HIV-1 in humans. Pandemic HIV-1 group M strains acquired Vpu-mediated anti-tetherin activity during human adaptation to overcome this hurdle. In contrast, HIV-1 group O Vpus do usually not counteract human tetherin. Instead, the accessory Nef protein of group O viruses evolved the ability to target a region adjacent to the deletion to antagonize tetherin in humans. Here, we demonstrate that the infectious molecular clone of HIV-1 O RBF206 utilizes both Nef and Vpu to antagonize human tetherin. Using FACS analyses and virus release assays, we show that the RBF206 Vpu is as efficient as the group M NL4-3 Vpu in reducing cell surface levels of human tetherin and promoting virus release. Unlike that of NL4-3, the RBF206 Vpu also efficiently antagonizes the second shorter isoform of humans tetherin that lack the first 12 amino acids. In the NL4-3 context, both Nef and Vpu reduce cell surface levels of human tetherin in infected PBMCs and promote virus release in 293T cells. Our data suggest that HIV-1 group O is still adapting to human tetherin and further illustrate the enormous capacity and plasticity of Vpu and Nef proteins in counteracting cellular defense mechanisms.

#### P43 The human endogenous retrovirus type K is involved in cancer stem cell markers expression and in human melanoma malignancy

##### Ayele Argaw-Denboba^1^, Emanuela Balestrieri^1^, Annalucia Serafino^2^, Ilaria Bucci^1^, Chiara Cipriani^1^, Corrado Spadafora^2^, Paolo Sinibaldi-Vallebona^1,2^, Claudia Matteucci^1^

###### ^1^University of Rome Tor Vergata, Department of Experimental Medicine and Surgery, Rome, Italy; ^2^National Research Council, Institute of Translational Pharmacology, Rome, Italy

**Correspondence:** Claudia Matteucci

*Retrovirology* 2016, **13(Suppl 1)**: P43

Increasingly scientific evidence underline retroelements and in particular human endogenous retroviruses (HERVs) as important players in cell plasticity, transformation and tumour progression. Expression of the HERV-K, especially the HML-2 family, was found elevated in melanoma and has been suggested to be implicated in the etiopathogenesis of the disease. We previously demonstrated that HERV-K activation and viral particles production were associated to aggressiveness and immune evasion of metastatic melanoma cells. However, melanoma consists of heterogeneous cell populations whose biological properties remain poorly characterised. In this context, phenotype-switching and cancer stem cell (CSC) models of melanoma progression are driven by genetic and epigenetic signalling, depending from microenvironment changes. Therefore, we investigated the potential role of HERV-K in cellular plasticity and stemness features of melanoma cells under modification of the microenvironment. To this aim, melanoma cell lines were exposed to different culture conditions; HERV-K dependency of cell phenotypic modifications, stem cell markers expression and metastatic features were evaluated. The study demonstrated that the plasticity features of melanoma cells were correlated and dependent to HERV-K activation. During modifications of the microenvironment, HERV-K expression was accompanied with the increase of cancer stem cell markers and with the switching towards an invasive malignant phenotype. Notably, the inhibition of HERV-K restrained cellular plasticity and stem cell markers expression. The understanding of the origin of stem-like cells plasticity and of multipotent phenotypes in the tumor would help the identification of new targets for therapy.

#### P44 Natural infection of Indian non-human primates by unique lentiviruses

##### S. Nandi Jayashree^1,2^, Ujjwal Neogi^3^, Anil K. Chhangani^4^, Shravan Sing Rathore^5^, Bajrang R. J. Mathur^6^

###### ^1^Albert Einstein College of Medicine, Dept. of Microbiology and Immunology, New York City, NY, United States; ^2^National Cancer Institute, Retrovirus Assembly Laboratory, HIV Dynamics and Replication Program, Frederick, MD, United States; ^3^Karolinska Institute, Division of Clinical Microbiology; F68, Department of Laboratory Medicine, Stockholm, Sweden; ^4^Maharaja Ganga Singh University, Department of Environmental Science, Rajasthan, India; ^5^Machiya Biological Park, Veterinary Center, Jodhpur Rajasthan, India; ^6^Kamla Nehru Nagar, 1B1, Jodhpur Rajasthan, India

**Correspondence:** Nandi Jayashree S.

*Retrovirology* 2016, **13(Suppl 1)**: P44

**Introduction:** Indian primates, rhesus macaques (*Macaca mulatta*) and langurs (*Semnopithecus entellus*) are not known to be naturally infected by SIVs. The reported ‘SIVmac’ is a variant of SIVsm that naturally infects African sootey mangabeys (*Cerocebus atys*). Cases of monkey bites are routinely reported from different parts of India, a potential source for zoonosis, or antropozoonosis, but consumption of monkey flesh is not common in India.

**Methods:** Plasma samples from the two common simian species were screened by HIV-1 WB assay as serologic tests for SIVs were not available in India, to investigate natural lentivirus infection of wild simians inhabiting north Indian Rajasthan forests. Antibodies cross reacting with some HIV antigens including gp120, p66, gp 41, p24 and p17 were present in a proportion of the plasma samples tested, suggesting lentivirus infection.

To molecularly characterise the unknown lentiviruses, DNA extracted from PBMCs and RNA extracted from plasma samples of multiple rhesus macaques and langurs were amplified by PCR and RT-PCR respectively, using pan-lentiviral primers from *pol* (RT, Protease), *gag* (p24, p17) and *env* (gp120, gp41) regions as well as LTR, and accessory genes *nef*, *vif and vpr.* The molecular virology work was conducted in the USA. Extensive phylogenetic analyses of the viral sequences were conducted independently in Sweden. Multiple sequence alignments were performed in AliView v1.17.1. Maximum likelihood phylogenetic analyses were performed in FastTreev1 and confirmed by MEGA6.

**Results:** The viral sequences revealed unexpected homology to Subtype B HIV-1, transmitted in some parts of north India. While the conserved p24 *gag* and RT (*pol*) sequences had exact homology with prototype subtype B HIV-1including HXB2, other region of *gag* (p17), partial *env* (gp120), *vpr*, *nef*, *vif* and LTR had distinct mutations, discounting any unintentional laboratory contamination. Intriguingly the sequences were not related to any known SIV, including SIVmac. The analysis identified the origin of LTR and *gag* sequences from HIV-1B sequences from north India with >80 % bootstrap support (LTR: NII_LTRS1; EU659806 and *gag*: B.IN.11807: EF694037). Lentiviruses infecting feral rhesus macaques and langurs clustered together with 74 % bootstrap support in the p17 *gag* region. *Vif* was truncated at the 3′region while *nef* at the 5′region. Importantly *nef* truncation occurred at equivalent conserved regions of both HIV-1 and SIV genomes, which could be the reason for the observed low to medium viral load in the simian plasma samples. The *env* gp120 sequences of lentiviruses infecting wild langur and rhesus macaques had several distinctive differences compared to HXB2, and were unrelated to equivalent sequences from SIVmac.

**Conclusion:** Reverse transmission of HIV-1 from infected humans to the simians through monkey bite is proposed. If the phenomenon is more widespread than this specific simian population, it could have profound consequences for the reservoirs of HIV-related lentiviruses in Asia and the rest of the world.

#### P45 Free cervical cancer screening among HIV-positive women receiving antiretroviral treatment in Nigeria

##### Adeyemi Abati

###### Luth, Publi Health, Lagos, Nigeria

**Correspondence:** Adeyemi Abati

*Retrovirology* 2016, **13(Suppl 1)**: P45

**Background:** Although the introduction of antiretroviral medications in resource-limited settings has decreased the number of HIV-positive women dying from AIDS, many are still at risk for HIV-related diseases including cervical cancer. Offering free cervical cancer screening at antiretroviral treatment centers may decrease the incidence of cervical cancer in this high-risk population.

**Methods:** HIV-positive women between the ages of 30 and 39 and receiving free antiretroviral treatment at the University of LAGOS TEACHING HOSPITAL AND CENTER FOR INFECTOIUS DISEASES were eligible for free cervical cancer screening. Eligible subjects were offered Pap smears during routine clinic visits. Pelvic examinations were performed by trained nurses and Pap smears were obtained using a slide, cervical brush and cytology fixative (UNIVERSITY OF IBADAN). Women with high-grade lesions were referred to UNIVERSITY OF CALABAR for colposcopic biopsy.

**Results:** Between July 20010 and June 2011, 595 eligible HIV-positive women were offered Pap smears, and 261 (44 %) accepted. Of the 261 women, 125 (48 %) women had normal cytological results, 112 (43 %) had abnormal cytological results, and 24 (9 %) had results which were indeterminate due to inflammation, inadequate sample collection, or insufficient data. Abnormal cytological results included 25 women (10 %) with atypical squamous cells of undetermined significance (ASCUS), 66 (25 %) low-grade squamous intraepithelial lesions (LSIL), and 21 (8 %) high-grade squamous intraepithelial lesions (HSIL). Of the 21 women with HSIL, 14 underwent colposcopic biopsy. Histology revealed 4 (29 %) CIN-I, 4 (29 %) CIN-II, 4 (29 %) CIN-III, and 2 (14 %) invasive cancers.

**Conclusions:** Among HIV-positive women receiving free antiretroviral treatment in Africa, we found significant abnormal cytological results and a large number of high-grade lesions. However, less than half of all eligible women accepted free screening. Further study is necessary to determine barriers to screening and the association of antiretroviral treatment and immunological status to cervical lesions

#### P46 Molecular evolutionary status of feline immunodeficiency virus in Turkey

##### B. Taylan Koç^1,2^, Tuba Çiğdem Oğuzoğlu^2^

###### ^1^Adnan Menderes University Faculty of Veterinary Medicine, Virology, Aydin, Turkey; ^2^Ankara University Faculty of Veterinary Medicine, Virology, Ankara, Turkey

**Correspondence:** B. Taylan Koç

*Retrovirology* 2016, **13(Suppl 1)**: P46

**Question:** Feline immunodeficiency virus (FIV) is one of the most significant infection agent among wild and domestic cats throughout the World, which belongs to *Retroviridae* family and also has been used as a model for the study of human immunodeficiency virus (HIV). Phylogenetic analyses, have been performed to date, indicated that FIV, as similar as HIV, has continually been diverged from each other due to genetic variations and genome integrations occurred between virus and host close interaction. Thus we aimed to investigate current molecular evolutionary status of FIV in Turkey.

**Methods:** The blood samples of 50 domestic cats were examined in term of FIV infection and six samples were found positive by *Polymerase Chain Reaction* (PCR). V3-V6 regions of the envelope gene (*env*) of six positive samples were characterised as well as many performed studies previously and *Maximum Likelihood* (ML) tree was constructed to detect diversities using other FIV strains from GenBank database.

**Results:** Some considerable results were obtained through this analysis. First one, data indicated that new characterised FIV strains has localized on separated clade from FIV A–E subtypes. Other one, it was found that these six FIV positive samples have been different from both by each other and older characterised Turkish FIV strains.

**Conclusion:** These results have shown that there was existence and prevalence of FIV infection among domestic cats in Turkey. Additionally the mentioned differences between FIV strains were required to be study with more samples relevant to whether or not using FIV vaccine and, choice criteria using vaccine.

### Topic 7: Innate sensing & intrinsic immunity

#### P47 Cell-to-cell contact with HTLV-1-infected T cells reduces dendritic cell immune functions and contributes to infection *in trans*

##### Takatoshi Shimauchi^1,2^, Stephan Caucheteux^2^, Jocelyn Turpin^3^, Katja Finsterbusch^2^, Charles Bangham^3^, Yoshiki Tokura^1^, Vincent Piguet^2^

###### ^1^Hamamatsu University School of Medicine, Department of Dermatology, Hamamatsu, Japan; ^2^Institute of Infection and Immunity, Cardiff University, Department of Dermatology, Cardiff, Great Britain; ^3^Imperial College London, Section of Virology, Department of Medicine, London, Great Britain

**Correspondence:** Vincent Piguet

*Retrovirology* 2016, **13(Suppl 1)**: P47

**Question:** Human T-lymphotropic virus type 1 (HTLV-1) infects between 5 and 10 million people worldwide. It causes the aggressive malignancy known as Adult T-cell Leukemia/Lymphoma (ATL), HTLV-1-Associated Myelopathy/Tropical Spastic Paraparesis (HAM/TSP), and infective dermatitis. Although strong immune responses are generated against the virus, they do not eliminate HTLV-1.

**Methods:** Primary human monocyte derived DCs were co-cultured with a HTLV-1 infected cell line, MT-2 with or without LPS. Morphological analyses for virus transmission were performed by confocal and electron microscopy. Virus binding or LPS-induced activation of DCs were analysed by FACS. Signaling pathways were also demonstrated by western blots. ALU-PCR or high-throughput integration site map analysis was performed by using purified DNA from DCs co-cultured with MT-2.

**Fig. 16:**
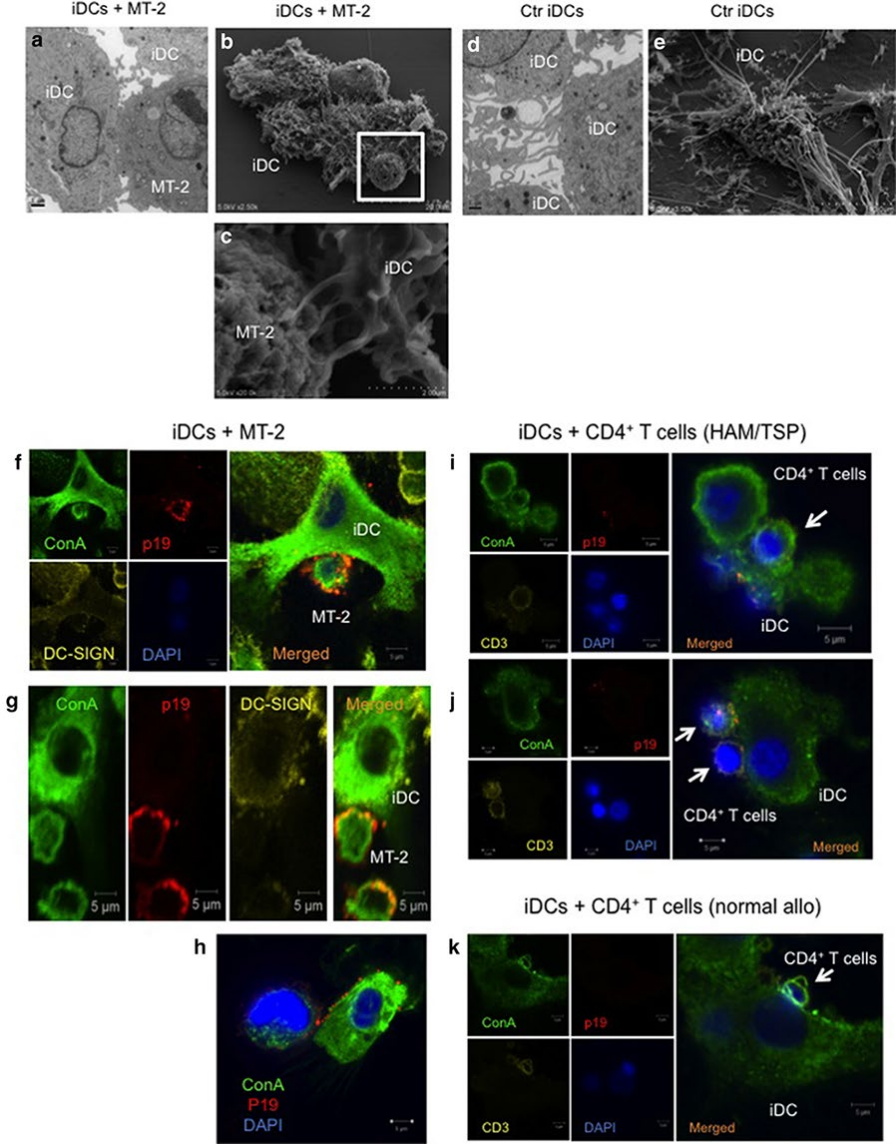
Transmission of HTLV-1 from infected cells to DCs via cell-to-cell contact. **a**, **d** TEM analysis of DC-MT-2 cell-to-cell contact or control DC. **b**, **c**, **e** SEM analysis of DC-MT-2 cell-to-cell contact or control DC. **f**–**k** Confocal immunofluorescence analysis of lectin-ConA (*green*), p19 Gag (*red*), DC-SIGN (*yellow*) or CD3 (*yellow*) of DCs co-cultured with MT-2, CD4^+^ T-cells from HAM/TSP or a normal allogeneic subject. DAPI (nucleus) is shown in *blue*

**Results:** We demonstrate that the contact formed between a DC and an HTLV-1-infected T cell can reduce DCs immunological functions by reducing TLR responses, IL-12 p40/p70 expression and MHC-II expression on the DCs. These DC-T cell contacts induce spleen tyrosine kinase-mediated c-Raf/MEK/ERK signaling pathway activation on DCs but are independent of DC-SIGN signalling. Furthermore, both Alu-PCR and high-throughput integration site analysis of HTLV-1 show little evidence of integrated proviruses in DCs. Finally, we show that DCs can re-transfer HTLV-1 to target autologous CD4^+^ T-cells in *trans* (Fig. 16).

**Conclusions:** These findings suggest that DC/T-cell virological synapses contribute not only to viral cell-to-cell transmission in trans, but also to down-modulation of host innate and adaptive immunity against HTLV-1. Restoring DC functions in HTLV-1 infection might improve the early immune control of the virus and reduce the risk of emergence of ATL and other diseases including infective dermatitis.

#### P48 Deciphering the mechanisms of HIV-1 exacerbation induced by *Mycobacterium tuberculosis* in monocytes/macrophages

##### Shanti Souriant^1^, Luciana Balboa^2^, Karine Pingris^1^, Denise Kviatcowsky^2^, Brigitte Raynaud-Messina^1^, Céline Cougoule^1^, Ingrid Mercier^1^, Marcelo Kuroda^3,4^, Pablo González-Montaner^5^, Sandra Inwentarz^5^, Eduardo Jose Moraña^5^, Maria del Carmen Sasiain^2^, Olivier Neyrolles^1^, Isabelle Maridonneau-Parini^1^, Geanncarlo Lugo-Villarino^1^, Christel Vérollet^1^

###### ^1^IPBS, CNRS UMR 5089, Toulouse, France; ^2^Instituto de Medicina Experimental (IMEX)-CONICET, Inmunologia de Enfermedades Respiratorias, Buenos Aires, Argentina; ^3^Tulane National Primate Research Center, Division of Immunology, Covington, LA, United States; ^4^School of Medicine, Tulane University, Department of Microbiology and Immunology, New Orleans, LA, United States; ^5^Hospital de Infecciosas Dr. F.J. Muñiz, Instituto Prof. Dr. Raúl Vaccarezza, Buenos Aires, Argentina

**Correspondence:** Shanti Souriant

*Retrovirology* 2016, **13(Suppl 1)**: P48

**Question:** AIDS is the deadliest disease due to a single infectious agent (*i.e.* Human Immunodeficiency Virus, HIV-1). Its well-known synergy with *Mycobacterium tuberculosis* (Mtb), the etiological agent for tuberculosis (TB), places an immense clinical and economic burden in resource-limited countries where co-infection with these pathogens is highly prevalent, and thus requires a better understanding at the cellular and molecular level. Interestingly, both pathogens impair the host immune response and share monocytes/macrophages as common host target cells. Our work aims at deciphering the mechanisms by which Mtb exacerbates HIV-1 infection in monocytes/macrophages.

**Methods:** Primary human monocytes were treated with either pleural effusions (PE) from TB patients (PE-TB) versus PE from patients with other pulmonary infections (PE-nonTB), or conditioned medium from Mtb-infected (CmMTB) versus uninfected (CmCTR) human macrophages. At day 3, conditioned cells were infected with HIV-1. Cell phenotype was assessed by flow cytometry, and macrophage infection was qualitatively and quantitatively characterised at day 13 using detection of p24 viral protein in confocal microscopy.

**Results:** We demonstrate that treatment of monocytes with PE-TB (or CmMTB) exacerbates HIV-1 infection (*i.e.* number of infected cells, virus entry and replication, formation of multinucleated giant cells), in comparison to treatment with PE-nonTB (or CmCTR). This increased HIV-1 infection is associated with a deregulator (M2c) activation program of monocytes/macrophages, characterised by the CD16^+^CD163^+^MerTK^+^CD169^+^ cell-surface marker signature, also observed on monocytes from the pleural cavity of TB patients. We identified specific molecular mechanisms responsible for these effects, including the increased expression of HIV-1 entry co-receptors CCR5 and CXCR4, and the IL-10/STAT3 signaling pathway.

**Conclusion:** Collectively, this study improves our understanding of how Mtb modulates the differentiation process of human monocytes towards a macrophage activation program that increases susceptibility to viral infection and formation of cell reservoirs for HIV-1. Finally, it will also provide novel target candidates with diagnostic and therapeutic potential against the co-morbidity established between AIDS and TB.

#### P49 The SAMHD1-mediated inhibition of LINE-1 retroelements is regulated by phosphorylation

##### Alexandra Herrmann^1^, Sabine Wittmann^1^, Caitlin Shepard^2^, Dominique Thomas^3^, Nerea Ferreirós Bouzas^3^, Baek Kim^2^, Thomas Gramberg^1^

###### ^1^Institute of Clinical and Molecular Virology, Erlangen, Germany; ^2^Emory University School of Medicine, Atlanta, GA, United States; ^3^Institute of Clinical Pharmacology, Frankfurt a. M., Germany

**Correspondence:** Alexandra Herrmann

*Retrovirology* 2016, **13(Suppl 1)**: P49

The SAM and HD domain-containing protein 1 (SAMHD1) blocks retroviral infection in nondividing myeloid cells and resting CD4^+^ T cells. SAMHD1 acts as a dNTP hydrolase and restricts viral infection by depleting cellular dNTPs below the level supporting reverse transcription. In addition, SAMHD1 has been shown to inhibit endogenous LINE-1 (L1) elements, the only autonomously active retroelements in humans.

In contrast to retroviral restriction, SAMHD1 blocks L1 elements also in dividing cells. To determine whether SAMHD1 inhibits HIV-1 and L1 through distinct mechanisms, we analysed SAMHD1-mediated restriction of L1 using a GFP-based L1 retrotransposition assay. In transiently transfected 293T cells, we found that co-expression of human and murine SAMHD1 reduced L1 retrotransposition. The activity of SAMHD1 against L1-GFP proofed to be regulated by phosphorylation of SAMHD1 at Threonine 592. Wildtype or SAMHD1 containing the phosphomimetic mutation T592D did not restrict L1, whereas the non-phosphorylated SAMHD1 T592A mutant potently inhibited L1. SAMHD1 activity was also dependent on the enzymatic active site and the allosteric dGTP-binding site. In addition, our data show that the intracellular localization of SAMHD1 is not critical for L1 restriction, since the ΔNLS-mutant also blocked retrotransposition. Quantification of intracellular dNTP levels indicated that the dNTPase activity alone might not be responsible for L1 restriction, hinting towards an additional mechanism of L1 restriction. Using a luciferase-based L1 promotor-assay, we found that SAMHD1 does not repress L1 promotor activity. Quantitative RT-PCR experiments suggest that SAMHD1 does not reduce L1 RNA levels and western blot analysis showed no decreased L1 protein levels upon co-expression of SAMHD1.

Together, our results demonstrate that SAMHD1 contributes to genome stability by restricting L1 retroelements. The mechanism of L1 restriction seems similar but not identical to that of HIV-1. In contrast to HIV-1, L1 is also blocked in dividing cells. Since neither the dNTPase nor the postulated RNase activity seem to contribute to L1 inhibition, further analyses of the L1 restriction might identify a previously unrecognized activity of SAMHD1.

#### P50 Activities of nuclear envelope protein SUN2 in HIV infection

##### Xavier Lahaye^1^, Anvita Bhargava^1^, Takeshi Satoh^1^, Matteo Gentili^1^, Silvia Cerboni^1^, Aymeric Silvin^1^, Cécile Conrad^1^, Hakim Ahmed-Belkacem^2^, Elisa C. Rodriguez^3^, Jean-François Guichou^4^, Nathalie Bosquet^5^, Matthieu Piel^6^, Roger Le Grand^5^, Megan King^3^, Jean-Michel Pawlotsky^2^, Nicolas Manel^1^

###### ^1^Institut Curie, Inserm, U932, Paris, France; ^2^Hôpital Henri Mondor, Department of Virology, Créteil, France; ^3^Yale University, School of Medicine, New Haven, CT, United States; ^4^CNRS, UMR5048, Montpellier, France; ^5^CEA, IDMIT Center, Fontenay-aux-Roses, France; ^6^Institut Curie, CNRS, UMR144, Paris, France

**Correspondence:** Xavier Lahaye

*Retrovirology* 2016, **13(Suppl 1)**: P50

HIV replication requires the successful orchestration of reverse transcription, nuclear entry, and integration while avoiding various antiviral factors and innate immune sensors during early steps of infection. The viral capsid and its interactions with cellular factors plays a major role during all of these steps. One of these factors, Cyclophilin A (CypA), binds the HIV capsid and is essential at these steps, but the underlying cellular pathway remained elusive. We identified a family of capsid mutants in HIV and SIVmac that are restricted by CypA. This antiviral restriction is maintained across species and inhibits nuclear import of the viral cDNA. We have demonstrated that the inner nuclear envelope protein SUN2 is required for this antiviral activity on HIV and SIV capsid mutants. In primary CD4+ target cells, wild-type HIV seems to manipulate SUN2 as an essential host factor for viral infection and SUN2 is required for the positive activities of CypA on reverse transcription and infection. Thus, these results identify fundamental CypA-dependent functions of SUN2 in HIV infection at the nuclear envelope. We are currently exploring the molecular mechanisms of SUN2 activities and associated factors at the nuclear envelope in HIV infection. The nuclear envelope is increasingly recognized as a highly versatile structure and it likely plays critical functions in HIV infection.

#### P51 Activation of TLR7/8 with a small molecule agonist induces a novel restriction to HIV-1 infection of monocytes

##### Henning Hofmann^1,2^, Benedicte Vanwalscappel^2^, Nicolin Bloch^2^, Nathaniel Landau^2^

###### ^1^Robert Koch Institut, HIV and other Retroviruses, Berlin, Germany; ^2^New York University, Microbiology, New York City, NY, United States

**Correspondence:** Henning Hofmann

*Retrovirology* 2016, **13(Suppl 1)**: P51

Myeloid cells such as monocytes, macrophages and dendritic cells sense virus infection using pattern recognition receptors (PRRs) including the toll-like receptors (TLRs) and RIG-I-like receptors (RLRs) that are activated by pathogen-associated molecular patterns (PAMPS). Sensing through these receptors activates a variety of antiviral systems and induces type-I interferon. In the case of HIV-1, PAMPs are thought to include the viral genomic RNA molecules, the reverse transcribed viral DNA and viral structural proteins. To determine whether TLRs could be further stimulated by small molecule agonists to potentiate innate anti-viral mechanisms, we tested a series of TLR agonists for their effects on HIV-1 infection of myeloid and T cells. One such molecule, the TLR7/8 agonist R848, induced a potent block to HIV-1 and HIV-2 in myeloid cells. We found that the block was post virus entry yet prior to or at reverse transcription. The block could not be overcome by packaging the Vpx accessory protein into the virus despite its maintained ability to degrade SAMHD1. Agonist treatment of bone marrow derived dendritic cells isolated from SAMHD1 knock-out mice also prevented HIV-1 infection, further suggesting a SAMHD1-independent restriction mechanism. Activation of TLR7/8 in monocytes caused the release of pro-inflammatory cytokines including type-I interferon (IFN). However, type-I IFN treatment of monocytes did not block reverse transcription in monocytes and IFN-blocking antibodies did not alleviate the R848-induced restriction, suggesting that the restriction was not caused by type-I IFN. Interestingly, the secreted cytokines blocked the infection of bystander cells. We conclude that viral sensing can be stimulated by small molecule agonists to both prevent the infection of target cells and protect bystanders. In target cells, the block to HIV infection is SAMHD1 and type-I IFN independent. This restriction could not be accounted for by any of the known restriction factors and thus is likely caused by a yet unidentified factor.

#### P52 Steady state between the DNA polymerase and Rnase H domain activities of reverse transcriptases determines the sensitivity of retroviruses to inhibition by APOBEC3 proteins

##### Stanislav Indik, Benedikt Hagen

###### University of Veterinary Med, Institute of Virology, Vienna, Austria

**Correspondence:** Stanislav Indik

*Retrovirology* 2016, **13(Suppl 1)**: P52

Members of the APOBEC3 (A3) protein family restrict replication of reverse transcriptase (RT)-containing viruses and other mobile elements. The most extensively studied A3 proteins, the human A3G and mouse mA3, restrict propagation of retroviruses by inducing deamination of deoxycytidine to deoxyuridine in the transiently single-stranded, minus-sense DNA (-ssDNA) intermediates during reverse transcription. Due to this specificity, the polypurine tract-proximal region, which remains single-stranded for the longest period of time, accumulates more mutations than the primer binding site-proximal region. To counteract A3 activity, retroviruses have evolved several strategies. These primarily include prevention of encapsidation of A3 proteins to virions either by a modification of the C terminus of nucleocapsid protein (HTLV-1) or by employing accessory proteins such as HIV-1 Vif and foamyvirus Bet. However, the majority of reverse-transcribing viruses lack obvious A3-neutralising factor and it is unlikely that they evade restriction by A3 avoidance like HTLV-1. One of such viruses is mouse mammary tumor virus (MMTV) that efficiently packages deamination-competent mA3 and A3G but is only partially inhibited by both restriction factors. We found that the lower sensitivity to inhibition results from a reduced frequency of deamination of the MMTV genome during reverse transcription governed by the MMTV RT. Specifically, we determined that the balance between the rate of DNA polymerization and the rate of RNA cleavage, which defines the extent of RNA degradation during the synthesis of the -ssDNA, is responsible for the low level of A3-mediated deamination of the MMTV reverse transcripts. Hence, the MMTV RT narrows down the window of opportunity for A3 to bind substrate. Residual mutagenic capacity of A3 proteins does not abolish virus infectivity and may be even exploited by MMTV to increase virus diversity and to modulate overall viral fitness. To our knowledge, alleviation of the deamination activity of A3 proteins, represents a novel mechanism of A3 counteraction and it seems likely that the same strategy is employed by other RT-containing viruses lacking Vif-like protein. Understanding of the A3 evasion mechanisms could be beneficial for improvement of current therapeutic protocols.

#### P53 HIV restriction in mature dendritic cells is related to p21 induction and p21-mediated control of the dNTP pool and SAMHD1 activity

##### José Carlos Valle-Casuso^1^, Awatef Allouch^1^, Annie David^1^, Françoise Barré-Sinoussi^1^, Michaela Müller-Trutwin^1^, Monsef Benkirane^2^, Gianfranco Pancino^1^, Asier Saez-Cirion^1^

###### ^1^Institut Pasteur, Virology, Paris, France; ^2^Institute of human genetics, CNRS UPR 1142, Montpellier, France

**Correspondence:** José Carlos Valle-Casuso

*Retrovirology* 2016, **13(Suppl 1)**: P53

**Background:** Dendritic cells (DCs) play a key role in the induction of immune responses against HIV. However, HIV has evolved ways to exploit them, facilitating immune evasion and viral dissemination. Immature myeloid DCs can sustain HIV-1 replication, in contrast mature myeloid DC are strongly resistant to HIV infection. Our group has recently shown that the cellular factor p21^cip/waf^ potently blocks HIV infection in macrophages by reducing the pool of dNTPs through the inhibition of RNR2. p21 through its cyclin-dependent kinase inhibitory activity might also modulate the phosphorylation state of SAMHD1 and its antiviral activity. We wondered whether p21 could be involved in the strong resistance of DCs to HIV infection.

**Results:** We found that the maturation of monocyte derived DCs (MDDCs), which strongly blocked HIV-1 replication, was associated with a strong increase in the expression of p21. Induction of p21 was accompanied by a decrease in the expression of RNR2 but also of the Thymidine kinase1 (TK1) and Thymidylate synthase (TYMS), which are critical for dTTP synthesis. Accordingly, we observed a decrease in the levels of all dNTPs in mature MDDCs. The knockdown of p21 expression recovered RNR2, TK1 and TYMS and the level of dNTPs, and increased infection. Supplementation with exogenous dNTPs recovered infection in mature MDDCs but not to the level observed in immature MDDCs. Maturation of MDDCs did not change total levels of SAMHD1 but was accompanied by a decrease in pSAMHD1 (inactive against HIV). Knockdown of p21 increased levels of pSAMHD1, supporting the role of p21 in the regulation of phosphorylation of SAMHD1 in DCs. Although degradation of SAMHD1 in the presence of VPX increased infection in mature MDDCs at great extent, the addition of exogenous dNTPs to MDDCs treated with VPL-VPX was necessary to completely rescue HIV infection.

**Conclusion:** Our results suggest that blocking HIV replication in mature MDDCs is due to an induction of p21 during maturation. p21 then regulates several factors involved in dNTPs synthesis and the phosphorylation of SAMHD1, resulting in conditions that allow efficient block of HIV-1 replication through complementary/synergistic mechanisms. Overall, p21 appears to be a key regulator of HIV infection in myeloid cells.

#### P54 IFITM protens restrict HIV-1 protein synthesis

##### Wing-Yiu Lee^1^, Chen Liang^2^, Richard Sloan^1^

###### ^1^Barts and The London School of Medicine, Blizard Institute, London, Great Britain; ^2^McGill University AIDS Centre, Lady Davis Institute, Montreal, Canada

**Correspondence:** Richard Sloan

*Retrovirology* 2016, **13(Suppl 1)**: P54

Interferon induced transmembrane proteins (IFITMs) restrict the cellular entry of a broad range of viruses, but it has been suspected that for HIV-1 IFITMs may also inhibit a post-integration replicative step. We investigated the effects of IFITM expression on late stage HIV-1 replication.

We found that the expression of human IFITMs reduces the quantity of released HIV-1, HIV-2, SIV and MLV viral particles from transfected cells. While knockdown of endogenous IFITMs enhances HIV-1 production. This phenotype is apparent during single cycle and multiple cycle infections of T-cells. Notably, IFITM-mediated antagonism is more potent for HIV-1 in which Nef has been deleted.

We show that IFITM expression reduces HIV-1 viral protein synthesis by preferentially excluding viral transcripts from translation and thereby restricts viral production. Codon-optimization of proviral DNA rescues viral translation during IFITM expression, implying that IFITM-mediated restriction targets viral RNA. While RRE-containing viral transcripts are more strongly excluded from translation when IFITMs are expressed, in agreement with a greater decrease of Vpu and Gag proteins, compared to multiply-spliced transcript levels and Nef.

IFITMs require cellular proteins to suppress viral production. Screening a panel of RNA helicases revealed that siRNA-mediated knockdown or expression of a dominant negative mutant of DDX3 impaired IFITM1-mediated restriction of HIV-1.

Further, mutation of cysteines in IFITM1 and IFITM2 that perturb their palmitoylation, and retention in plasma membrane and endosomes, strongly reduces levels of all classes of viral transcripts and potently inhibits viral output far beyond the level of restriction already seen for wild-type IFITM proteins.

Our studies identify a novel role for IFITMs in inhibiting HIV-1 replication at the level of translation and for which viral RNA is a determinant. Yet we show that this restriction can be overcome by the lentiviral countermeasure Nef. Further, we identify the host RNA helicase DDX3 as a cofactor for restriction of HIV-1 protein synthesis by IFITM1. We also show that it is possible to engineer highly active anti-HIV-1 IFITM variants by modulating their palmitoylation status.

#### P55 Characterisation and functional analysis of the novel restriction factor Serinc5

##### Bianca Schulte, Silvana Opp, Felipe Diaz-Griffero

###### Albert Einstein College of Medicine, Microbiology and Immunology, New York City, NY, United States

**Correspondence:** Bianca Schulte

*Retrovirology* 2016, **13(Suppl 1)**: P55

In recent years several human proteins have come to light which act as restriction factors for HIV-1 infection. Among these Serinc3 and Serinc5, members of the Serinc family of transmembrane proteins could recently be shown to have strong anti-HIV-1 activity if expressed in virus-producing cells. The exact mechanism by which Serinc3 and Serinc5 restrict HIV-1 is as of yet unknown. Therefore, we have analysed different regions of Serinc5, the most potent restriction factor of the family, to discover those regions of the protein which are crucial for restriction. To achieve this, we systematically examined the protein by means of domain deletions, domain swaps, amino acid mutations and construction of chimeric proteins. Beyond the extensive mapping of the protein’s function, we were able to characterise the membrane topology of this 10-transmembrane protein, but most importantly, we have devised several assays to define the protein’s function in HIV-1 restriction: Analysis of surface expression, total expression by Western blot and flow cytometry, localisation, infectivity, particle incorporation, gag and env maturation, and its influence on antibody-mediated virus neutralisation. With the help of these methods we collected many insights into the protein’s domain structure, localization and mechanism of action.

#### P56 piRNA sequences are common in Human Endogenous Retroviral Sequences (HERVs): an antiretroviral restriction mechanism?

##### Jonas Blomberg^1^, Luana Vargiu^1,2,3^, Patricia Rodriguez-Tomé^1,2,3^, Enzo Tramontano^1,2,3^, Göran Sperber^1,2,3,4^

###### ^1^Uppsala University, Medical Sciences, Uppsala, Sweden; ^2^Cagliari University, Life and Environmental Sciences, Cagliari, Sweden; ^3^Nurideas SRL, Cagliari, Sweden; ^4^Uppsala University, Neuroscience, Uppsala, Sweden

**Correspondence:** Jonas Blomberg

*Retrovirology* 2016, **13(Suppl 1)**: P56

**Background:** One of several inhibitory RNA (RNAi) systems is the piwi anti-transposon system, which stores short (24–32 nt) concatenated transposon copies in genomic DNA. It is especially active during early embryogenesis. It is not known if the piwi system is active against retroviruses and related retrotransposons in humans. We therefore searched for sense and antisense piwi sequences in our collection of HERV sequences, and in random sequences of a similar length.

**Materials and methods:** The following sequence collections were used: 3290 proviruses found by the RetroTector program [Sperber et al. NAR (2007); 35:4964–76] in the GRCh 37/hg19 human genome assembly (HG19), 3150 random 5000 bp sequences and 23,595 human entries of the piRNA database [Lakshmi and Agarwal, NAR (2008); 36: D173–D177]. BLASTn was downloaded from the NCBI website. The runs were administered by programs written in Visual Foxpro by JB.

**Results:** Of 3290 proviruses in HG19 870 contained a perfect copy (100 % match) of at least one piRNA (558 had one, 166 two, 72 three, 33 four, 16 five, 14 six, 7 seven, 4 eight, average 2.07 per provirus). Relative to the ordinary 5′–3′sense of retroviruses (rvsense, s), 42 % of the piwi sequences were in rvsense, 58 % in antirvsense (as). None of the random sequences contained piwi sequences. The frequency of piwi sequences in 5′LTRs was 118 (37 % s and 63 % as); in 3′LTR 94 (42 % s, 58 % as); in gag 126 (44 % s, 66 % as); in pro 54 26 % s, 74 % as; in pol 376 (39 % s, 61 % as); in env 132 (35 % s, 65 % as). Proviruses containing piwi sequences occurred on all chromosomes.

**Conclusion**: HERVs may participate in piwi-dependent RNAi retroviral restriction.

#### P57 Ferroportin restricts HIV-1 infection in sickle cell disease

##### Namita Kumari, Tatiana Ammosova, Sharmeen Diaz, Patricia Oneal, Sergei Nekhai

###### Howard University, Medicine, Washington D.C., United States

**Correspondence:** Sergei Nekhai

*Retrovirology* 2016, **13(Suppl 1)**: P57

Low occurrence of HIV-1 infection in sickle cell disease (SCD) patients suggests a potential restriction of HIV-1 infection in SCD. Here we analysed whether ex vivo HIV-1 infection is restricted in SCD PBMCs and determined the mechanism of the restriction. We observed reduced HIV-1 replication and increased expression of heme and iron-regulated genes, including ferroportin. We also observed the induction of several HIV-1 regulatory host factors, including SAMHD1. Critically, HIV-1 restriction was alleviated with hepcidin treatment suggesting that ferroportin mediates HIV-1 restriction which might go unsuppressed as hepcidin levels were not elevated in serum of SCD patients. Consistent with the increased ferroportin expression, labile iron levels were reduced in SCD PBMCs and protein levels of ferroportin and HIF-1α were increased. The effect of hepcidin was replicated in HIV-1 infected primary and cultured cells, in which HIV-1 inhibition by hemin was reversed by hepcidin. Knock down of ferroportin, HO-1 but not HIF-1α alleviated the HIV-1 inhibition by hepcidin. Analysis of HIV-1 replication steps showed inhibition of reverse transcription, implicating SAMHD1. SAMHD1 is negatively regulated by CDK2, which is inhibited by iron chelation. In SCD PBMCs, CDK2 activity was low and SAMHD1 phosphorylation was reduced. SAMHD1 expression was induced and its phosphorylation decreased with heme treatment further supporting its involvement in HIV-1 restriction in SCD. Our findings point to the previously unknown role of ferroportin as an HIV-1 restriction factor in biologically relevant settings, linking reduced intracellular iron levels to the inhibition of CDK2, reduction of SAMHD1 phosphorylation and HIV-1 inhibition.

#### P58 APOBEC3G modulates the response to antiretroviral drugs in humanized mice

##### Audrey Fahrny^1^, Gustavo Gers-Huber^1^, Annette Audigé^1^, Roberto F. Speck^1^, Anitha Jayaprakash^2^, Ravi Sachidanandam^2^, Matt Hernandez^3^, Marsha Dillon-White^3^, Viviana Simon^3,4^

###### ^1^University hospital Zurich, Infectious diseases, Zurich, Switzerland; ^2^Icahn School of Medicine at Mount Sinai, Department of Oncological Sciences, New York City, NY, United States; ^3^Icahn School of Medicine at Mount Sinai, Department of Microbiology and The Global Health and Emerging Pathogens Institute, New York City, NY, United States; ^4^School of Medicine at Mount Sinai, Division of Infectious Diseases, Department of Medicine, New York City, NY, United States

**Correspondence:** Audrey Fahrny

*Retrovirology* 2016, **13(Suppl 1)**: P58

Viral evolution and diversification in HIV+ patients have been associated with increased pathogenicity and underlies the rapid appearance of viral variants resistant to antiretroviral drugs. APOBEC3G (A3G) restricts HIV-1 by inducing G-to-A mutations in the newly synthesized proviral cDNA during RT. The HIV-1 accessory protein Vif counteracts A3G restriction and is, thus, critical for productive HIV-1 replication in vivo. Humanized mouse models provide us with an experimental system to directly test the extent to which sub-optimal neutralisation of A3G impacts viral fitness, pathogenicity and antiretroviral treatment outcomes. CD34 complemented NSG mice were infected with HIV strains that encoded Vif variants differing in anti-A3G activity (e.g., WT: 100 %, 45G: 10 % and SLQ: 1 %). In a subset of mice antiretroviral treatment with lamivudine (3TC) was initiated after 4 weeks of infection. Plasma viremia and T cell subsets were measured for up to 4 months’ post infection. HIV WT and 45G viruses established productive infection and displayed comparable levels of plasma viremia at 4 weeks post infection. In contrast, HIV SLQ failed to infect and spread in humanized mice suggesting that counteracting A3G is needed. Interestingly, we observed that the WT infected mice maintained high levels of plasma viremia while the 45G infected mice displayed a significant reduction in plasma viremia over time. As expected, 3TC treatment decreased viremia in all infected animals but the 45G infected mice experienced less overall reduction in viremia and a more rapid viral rebound. In summary, viruses engineered to display a range of anti-APOBEC3 activities are useful tools to probe for impact of APOBEC3 on viral pathogenesis and treatment outcome. Our results suggest that partial neutralisation of APOBEC3 impacts viral fitness and response to lamivudine. Ongoing and future studies will analyze the genotypes (e.g., RT, Vif) underlying the observed in vivo phenotypes.

#### P59 High-throughput epigenetic analysis of evolutionarily young endogenous retrovirus presents in the mule deer *(Odocoileus hemionus*) genome

##### Tomas Hron, Helena Farkasova, Daniel Elleder

###### Institute of Molecular Genetics, ASCR, Laboratory of Viral and Cellular Genetics, Prague, Czech Republic

**Correspondence:** Tomas Hron

*Retrovirology* 2016, **13(Suppl 1)**: P59

Endogenous retroviruses (ERVs) are genetic elements constituting a significant part of the vertebrate genomes. They are generated when an exogenous virus integrates into the host germline which leads to vertical transfer of ERVs to subsequent host generations. ERVs are usually fixed in the host population for millions of years and their sequences are damaged by mutations. However, small portion of ERVs retains intact genetic information and have been recently shown to play a key role in various cellular processes and pathologies. Study of the DNA methylation-dependent transcriptional silencing, the main mechanism of host defence against uncontrolled virus propagation in its genome, is crucial for uncovering the ERV-host interactions. Despite the progression in this field, the involvement of epigenetic regulations in the defense against active ERVs is still poorly understood.

We study a recently identified ERV present in the mule deer genome, CrERV. This evolutionary young virus is extremely polymorphic in its integrations suggesting an ongoing invasion into the host genome. This makes CrERV a unique model for studying retrovirus endogenisation. In our work, we employed next generation bisulfite sequencing strategy to determine the methylation pattern of individual CrERV integrations in different animals. This method offers new insight into the interactions between host and active ERVs.

#### P60 Characterisation of the expression of novel endogenous retroviruses and immune interactions in a macaque model

##### Neil Berry^1^, Emmanuel Maze^2^, Claire Ham ^1,2^, Neil Almond^1^, Greg Towers^3^, Robert Belshaw^2^

###### ^1^NIBSC, Virology, South Mimms, Great Britain; ^2^Plymouth University Peninsula Schools of Medicine and Dentistry, Plymouth University Peninsula Schools of Medicine and Dentistry, Plymouth, Great Britain; ^3^University College London, Infection and Immunity, London, Great Britain

**Correspondence:** Neil Berry

*Retrovirology* 2016, **13(Suppl 1)**: P61

**Background and question:** Endogenous retroviruses (ERVs), descendents of retroviruses integrated into host germline cells which have proliferated over millions of years, represent ~5 % of our and other mammal genome sequences. Recent research indicate ERVs (and other retroelements such as LINEs) may play a role in innate sensing, with implications for combating autoimmune disease and viral infection. We have previously shown the macaque to have recently integrated ERVs belonging to three different lineages. Archived material from past macaque/SIV studies provides an ideal opportunity to examine the dynamics of ERV and SIV expression in the context of their interaction with the innate immune response. Specifically, we plan to explore Volkman & Stetson’s (2014) hypothesis that endogenous retroelement expression affects the threshold for the innate response to exogenous viral infection.

**Methods:** Generation of in vitro transcripts and specific oligonulcleotide primer and probe sequences have enabled development of qPCR assays for the three ERV lineages in the macaque shown to contain recently integrated loci. We have also searched bioinformatically for full-length ORFs in the reference rhesus macaque genome sequence in order to predict the potential of these lineages to produce virions and hence RNA in the plasma.

**Results:** ERV-specific RNA and DNA signals appear to increase in both Mauritian cynomolgus and Indian macaque species during acute infection with several wild-type exogenous SIV strains. The relationship between expression of ERV-specific lineages and SIV RNA during acute infection is currently being explored, in tandem with induction of an innate response detectable during acute infection.

**Conclusions:** Preliminary elucidation of levels of ERV-specific RNA and cell-free DNA in macaque plasma suggest a dynamic relationship with exogenous SIV infection. Localised expression of ERV mRNA in cell-associated SIV infection in conjuction with upregulation of interferon stimulated genes in multiple macaque challenge studies will provide a fuller picture of the potential dynamic interplay of these parameters in the outcome of infection.

#### P61 HIV-1 restriction by orthologs of SERINC3 and SERINC5

##### Patrícia de Sousa-Pereira^1,2,3,4^, Joana Abrantes^2^, Massimo Pizzato^5^, Pedro J. Esteves^2,3,6^, Oliver T. Fackler^7^, Oliver T. Keppler^1,4^, Hanna-Mari Baldauf^1,4^

###### ^1^Max von Pettenkofer Institut, LMU, Munich, Germany; ^2^CIBIO/InBIO, University of Porto, Vairão, Portugal; ^3^Faculty of Sciences, University of Porto, Porto, Portugal; ^4^Institute of Medical Virology, University of Frankfurt, Frankfurt a. M., Germany; ^5^University of Trento, Centre for Integrative Biology, Trento, Italy; ^6^CITS, CESPU, Gandra, Portugal; ^7^Department of Infectious Diseases, Integrative Virology, University Hospital Heidelberg, Heidelberg, Germany

**Correspondence:** Patrícia de Sousa-Pereira

*Retrovirology* 2016, **13(Suppl 1)**: P62

The recent discovery of SERINC3 and SERINC5 as an innate immunity factor acting against HIV revealed another example of how virus-host interactions control pathogen replication. The current knowledge is that SERINC3 and SERINC5 expression leads to markedly reduced virion infectivity in the absence of Nef. The exact mechanism of action of SERINC restriction and Nef antagonism are currently elusive and domains essential for SERINC’s function are unknown.

As SERINC proteins are present in several eukaryotes’ genome, the aim of the current work is to assess the ability of rodent and lagomorph SERINC orthologs to interfere during HIV-1 replication and to identify thereby essential domains and the mode of action, as well as their importance for the generation of an animal model for HIV replication/pathogenesis.

cDNAs of SERINC3 and SERINC5 from mouse, rat and rabbit were amplified by PCR and cloned into expression vectors. After co-transfection of SERINC orthologs with HIV-1deltaNef proviral DNA into 293T cells, the effect on HIV-1 infectivity was assessed by measuring RT activity of released viral particles using SG-PERT, and firefly luciferase activity on TZM-bl cells of viral supernatants. Viral entry into new target cells was also measured using a FACS-based virion-fusion assay. In addition, evolutionary analyses were conducted to evaluate the existence of selective pressure and to determine the mutation rate in SERINC3 and SERINC5 from different mammal species.

Although a certain level of amino acid divergence was observed, all SERINC3 and SERINC5 orthologs displayed an anti-HIV activity comparable to the human orthologs. The first results obtained with the FACS-based virion fusion assay correlate with the data from the infectivity assay, showing a lower activity of SERINC3 in restricting HIV infectivity when compared with SERINC5. The assessment of the potency by the viral antagonist Nef to overcome the imposed restriction is currently in progress.

Our results show that rodent and rabbit SERINC3 and SERINC5 inhibit HIV-1 infectivity in a different extent but comparable with the human orthologs. The antiviral activity of SERINC proteins thus appears to be extended to rodents and rabbits, therefore representing a potential restriction factor when using these species as an animal model for HIV replication.

#### P62 TRIM19/PML restricts HIV infection in a cell type-dependent manner

##### Bianca Volkmann^1^, Tanja Kahle^1^, Kristin Eissmann^1^, Alexandra Herrmann^1^, Sven Schmitt^2^, Sabine Wittmann^1^, Laura Merkel^1^, Nina Reuter^1^, Thomas Stamminger^1^, Thomas Gramberg^1^

###### ^1^Friedrich-Alexander University Erlangen-Nuremberg, Institute of Clinical and Molecular Virology, Erlangen, Germany; ^2^University Hospital Bonn, Institute of Clinical Chemistry and Pharmacology, Bonn, Germany

**Correspondence:** Bianca Volkmann

*Retrovirology* 2016, **13(Suppl 1)**: P63

The promyelocytic leukemia protein (PML), also named TRIM19, is a member of the TRIM protein family. The seven main PML isoforms are located both in the cytoplasm and the nucleus. In the nucleus, PML is mostly sumoylated and is the main structural component of the nuclear matrix structures known as nuclear domain 10 (ND10) or PML nuclear bodies (PML-NBs). ND10 structures, in which PML is associated with Sp100, Daxx and other proteins, have been shown to mediate an intrinsic immune response against various different viruses. In this study, we analysed the role of PML during retroviral replication in different cell types using cell lines exhibiting a shRNA-mediated knockdown of PML, Daxx and SP100. Whilst the permanently ND10 associated proteins Daxx and SP100 have no restricting effect, PML inhibits HIV in a cell type-dependent manner. HIV reporter virus infection assays comparing control cells harboring intact PML and PML-knockdown cell lines revealed an active PML-mediated block to retroviral infection in primary human fibroblasts and murine embryonic fibroblasts. However, this block to HIV reporter virus infection was not present in T cell lines, e.g. Molt4 or Jurkat, and myeloid cell lines, e.g. CEM and HuT78. Quantitative PCR analysis of HIV cDNA in infected cells revealed that PML restricts infection at the level of reverse transcription, which occurs in the cytoplasm after virus entry. Indeed, in immunofluorescence analysis of infected human fibroblasts we found a temporary relocation of PML from the nucleus to the cytoplasm, which started 30 min post infection and increased till 4 h post infection. Overexpression of the PML isoforms I to VI resulted in a moderate reduction of HIV reporter virus infectivity, but failed to reveal a particular isoform responsible for the PML-mediated block to retroviral infection. Finally, we showed that PML has antiviral activity against other members of different genera of the retroviral family, e.g. SIV, MPMV and MLV. Our findings shed light on the controversial role of PML during retroviral infection and show that PML contributes to the intrinsic restriction of retroviral infections in a cell type-dependent manner.

#### P63 Recent invasion of the mule deer genome by a retrovirus

##### Helena Farkasova, Tomas Hron, Daniel Elleder

###### Institute of Molecular Genetics of the ASCR, Laboratory of Viral and Cellular Genetics, Prague, Czech Republic

**Correspondence:** Helena Farkasova

*Retrovirology* 2016, **13(Suppl 1)**: P64

Endogenous retroviruses (ERVs) originate by germline infection and subsequent mendelian inheritance of their exogenous counterparts. With notable exceptions, all mammalian ERVs are evolutionarily old and fixed in the population of its host species. Broader knowledge about the process of endogenization is lacking.

Besides endogenous retrovirus in koalas, ERV in mule deer (*Odocoileus hemionus*) forms new germline insertions in the natural host population in the present time and serve as important model of the retrovirus endogenization process. Previously, we have determined complete genome sequence of the deer ERV, denoted cervid endogenous retrovirus (CrERV). Using next generation sequencing-based approach, we have characterised thousands of highly polymorphic CrERV integrations in approximately 50 animals. Notable polymorphism within the population of mule deer with integration sites allocated to specific area verify the predicted young age of the virus as well as the current process of endogenization.

For virological studies, we have obtained an infectious virus by cocultivation with susceptible human cells and performed experiments to characterise its biophysical properties. Subsequently, we have constructed an infectious molecular clone of CrERV and defined its basic replication properties. Surprisingly, CrERV exhibits a xenotropic behaviour, where it infects human cells but not original host deer cells. This is in contrast with the efficient generation of many new endogenous integrations. We are using reciprocal pseudotypes with murine leukemia virus (MLV) and vesicular stomatitis virus envelope (VSV) bearing a GFP signal, to characterise the nature of the replication block on mule deer cells. The preliminary data point to the block at the receptor level.

#### P64 cGAMP transfers intercellularly via HIV-1 Env-mediated cell–cell fusion sites and triggers an innate immune response in primary target cells

##### Shuting Xu^1^, Aurélie Ducroux^1^, Aparna Ponnurangam^1^, Sergej Franz^1^, Gabrielle Vieyres^1^, Mathias Müsken^2^, Thomas Zillinger^3^, Angelina Malassa^1^, Ellen Ewald^1^, Veit Hornung^4^, Winfried Barchet^3^, Susanne Häussler^2^, Thomas Pietschmann^1^, Christine Goffinet^1^

###### ^1^TWINCORE, Experimental Virology, Hanover, Germany; ^2^TWINCORE, Molecular Bacteriology, Hanover, Germany; ^3^University of Bonn, Clinical Chemistry and Clinical Pharmacology, Bonn, Germany; ^4^Ludwig-Maximilians-University Munich, Gene Center and Department of Biochemistry, Munich, Germany

**Correspondence:** Shuting Xu

*Retrovirology* 2016, **13(Suppl 1)**: P66

Upon virus infection, cGAS-cGAMP-STING-dependent type I IFN signaling contributes to achieve an antiviral state in the host cells. Work from others suggests that cGAMP is transferred intercellularly via gap junctions and/or incorporated into newly synthesized viral particles. The goal of our study is to investigate whether intercellular transfer of cGAMP occurs via HIV-1 Env-mediated cell–cell fusion.

Primary human macrophages that were cocultured with cGAS-positive CHO cells, but not cGAS-negative Jurkat cells expressing HIV-1 Env induced human IFN-beta mRNA expression and scored positive in a bioactivity assay for type I IFNs. Reduction of cGAS expression in CHO cells and reconstitution of cGAS expression in Jurkat cells abolished and rescued the mounting of the IFN response, respectively, in cocultured macrophages. Importantly, donor cell line-specific cGAS expression levels and abundance of SVPDE-sensitive, IFN-inducing small molecules, most conceivably cGAMP, correlated with the ability to trigger an IFN response in cocultured macrophages. Additionally, an intact STING-TBK1-IRF3 signaling axis, but not the cytoplasmic DNA sensor cGAS in macrophages, was essential for the macrophages’ ability to induce an IFN response upon coculture.

Mounting of an IFN response coincided with a functional antiviral state in macrophages and was abolished upon genetic, immunological or pharmacological interference with the membrane fusion process. Whereas HIV-1 Env-positive donor cells and target macrophages interacted via multiple modes, only fusion inhibitor-sensitive cell–cell fusion, but not Jasplakinolide-sensitive phagocytosis of donor cells by macrophages was required for induction of an IFN response.

In conclusion, we propose that cGAMP transfers horizontally through HIV-1 Env-mediated membrane fusion sites from cells with activated or constitutively active cGAS to cocultured macrophages, and activates an innate immune response. The intercellular transfer of this second messenger may have direct implications in the context of cell-to-cell transmission of HIV-1.

#### P65 Pre-infection transcript levels of *FAM26F* in PBMCS inform about overall plasma viral load in acute and postacute phase after SIV-infection

##### Ulrike Sauermann^1^, Aneela Javed^1,2^, Nicole Leuchte^1^, Gabriela Salinas^3^, Lennart Opitz^3,4^, Christiane Stahl-Hennig^1^, Sieghart Sopper^5^

###### ^1^Deutsches Primatenzentrum GmbH, Infektionsmodelle, Göttingen, Germany; ^2^National University of Sciences and Technology, Atta-ur-Rahman School of Applied Biosciences (ASAB), Islamabad, Pakistan; ^3^Faculty of Medicine, University Göttingen, Transcriptome and Genome Analysis Laboratory (TAL), Göttingen, Germany; ^4^Functional Genomics Center Zurich, Swiss Federal Institute of Technology, Zurich, Switzerland; ^5^Medical University Innsbruck and Tyrolean Cancer Research Institute, Tumor Immunology Lab, Innsbruck, Austria

**Correspondence:** Ulrike Sauermann

*Retrovirology* 2016, **13(Suppl 1)**: P67

CD8+ cells from simian immunodeficiency virus (SIV)-infected long term non progressors and certain uninfected macaques can suppress viral replication in vitro (CNAR). Analysis of the global transcription pattern and additional validation revealed that expression of *FAM26F* distinguished CD8+ cells controllers and non controllers.

However, the studies also indicated that the cell surface protein FAM26F might be not necessarily related to CNAR, but to immune activation which provided the rationale for further investigations into *FAM26F* expression and its role in SIV-infection. *FAM26F* was also expressed in many cells of the immune system. Its expression increased in vitro after IFN-γ treatment of lymphocytes. *Ex vivo FAM26F* RNA levels in PBMCs correlated with plasma IFN-γ but not with IFN-α indicating that *FAM26F* transcription is linked to the IFN-γ pathway. Baseline FAM26F expression appeared to be stable for months, albeit the individual expression levels varied significantly. *FAM26F* expression in macaques thus might represent an eQTL similar to humans. *FAM26F* transcription was upregulated in SIV-infected monkeys, but did not directly correlate with viral load in contrast to *MX1* and *CXCL10*. Notably, pre-infection levels of *FAM26F* correlated inversely with overall plasma viral load during the acute and post-acute phase of infection (AUC wpi 0–8) in naïve SIV-infected from two experiments, and—at a lower significance level—even in immunized macaques.

*FAM26F* transcript levels prior to infection thus can inform about the pace and strength of the antiviral immune response during the early stage of infection. It has a strong potential to serve as a novel marker to investigate innate and adaptive immunological responses limiting early retroviral replication.

### Topic 8: Adaptive immunity & immune evasion

#### P66 Sequence-function analysis of three T cell receptors targeting the HIV-1 p17 epitope SLYNTVATL

##### Christiane Mummert^1^, Christian Hofmann^1,2,3^, Angela G. Hückelhoven^1,4^, Silke Bergmann^1^, Sandra M. Müller-Schmucker^1,5^, Ellen G. Harrer^1^, Jan Dörrie^2^, Niels Schaft^2^, Thomas Harrer^1^

###### ^1^Universitätsklinikum Erlangen, Infectious Diseases Section, Department of Internal Medicine III, Erlangen, Germany; ^2^Universitätsklinikum Erlangen, Department of Dermatology, Erlangen, Germany; ^3^University of California, Division of Infectious Diseases, Los Angeles, LA, United States; ^4^University Hospital Heidelberg, Department of Internal Medicine 5, Heidelberg, Germany; ^5^Friedrich-Alexander-Universität Erlangen-Nuremberg, Institute of Clinical and Molecular Virology, Erlangen, Germany

**Correspondence:** Christiane Mummert

*Retrovirology* 2016, **13(Suppl 1)**: P68

**Background:** T-cell receptor (TCR) transfer is a promising approach for boosting the cytotoxic T lymphocyte (CTL) response. Due to the ability of HIV-1 to escape from CTLs it is important to use TCRs with broad recognition of viral variants. So far, there is a lack of data regarding the molecular determinants for the recognition of defined epitopes. Therefore, we performed a sequence-function analysis of three different TCRs targeting the HLA-A2-restricted CTL epitope SLYNTVATL (SL9) in HIV-1 p17.

**Methods:** Three TCRs were cloned from SL9-specific CTL from three HIV-1-infected patients. SL9/TCR-mRNA-constructs encoding the TCR-alpha and TCR-beta chains were electroporated into peripheral blood mononuclear cells (PBMC). TCR functionality with regard to functional avidity and cross-recognition of eleven SL9 variants was analysed in γIFN-ELISPOT assays using synthetic peptides.

**Results:** The SL9-specific TCRs consisted of following TCR-alpha and TCR-beta chains: TCR #1: AV2S1/BV5S1(Cβ1), TCR#2: AV2S1/BV5S1(Cβ2). TCR#3: AV2S3/BV22S1(Cβ2). TCR#1 and TCR#2 used the same variable alpha and beta chains, but displayed variant amino acids in the CDR3-regions of the TCR-alpha and TCR-beta chains. In addition, TCR#1 and #2 used different constant beta chains.

All three TCRs showed a similar functional avidity with a half-maximal peptide sensitizing concentration ranging between 2 μg/ml and 0.2 μg/ml. Recognition of viral variants was similar for TCR#1 and TCR#2, whereas TCR#3 showed a different pattern of variant recognition.

Cross-over exchange of the TCR-alpha and TCR-beta chains from TCR#1 and TCR#2 did not affect SL9-recognition by the new hybrid TCRs. In contrast, hybrid TCRs with combinations of the respective TCR-alpha and TCR-beta chains from TCR#1/TCR#3 and TCR#2/TCR#3 failed to recognize SL9.

**Conclusion:** Despite their differences in the sequences of their TCR-alpha and TCR-beta chains, all three HLA-A2-restricted TCRs showed a similar functionality with regard to peptide avidity. The efficacy of a TCR-based immunotherapy could be enhanced by usage of different TCRs targeting the same epitope with a different pattern of recognition of viral sequence variants.

#### P67 An immunodominant region of the envelope glycoprotein of small ruminant lentiviruses may function as decoy antigen

##### Laure Cardinaux, M.-L. Zahno, H.-R. Vogt, R. Zanoni, G. Bertoni

###### University of Bern, Institute of Virology and Immunology, Vetsuisse Faculty, Bern, Switzerland

**Correspondence:** Laure Cardinaux

*Retrovirology* 2016, **13(Suppl 1)**: P69

Small ruminant lentiviruses persist in infected goats that mount a strong humoral immune response characterised by low neutralising titers. In this study, we characterised the antibody response to SU5, a variable, immunodominant epitope of the envelope glycoprotein of SRLV (1,4). The sequence of this epitope is variable between different phylogenetic groups but surprisingly conserved within a given subgroup (3,4). We compared the neutralising activity of the affinity purified anti-SU5 fraction with that of antibody contained in the unfractionated serum, the flow through of the affinity column and a negative control serum. While the neutralising activity of the flow through associated antibody was not significantly reduced compared to the unfractionated serum, the purified anti-SU5 antibody was devoid of neutralising activity. Taken together these data strongly suggest that anti-SU5 antibody is incapable of binding the functional form of Env expressed at the surface of infectious virus particles. We concluded that the observed variability does not reflect escape from neutralisation but rather indicate a structural tolerance of this particular region. To test this hypothesis, we substituted the original SU5 of the g6221 molecular clone with a FLAG-Tag (2). Preliminary data indicate that the mutated virus replicates efficiently in susceptible cells, confirming the structural tolerance of this particular region of the envelope gene. Experiment in vivo will follow to confirm the immunodominant nature of this envelope region. We propose that SU5 is a decoy epitope exposed on virus debris that lures the humoral immune response in committing an “original epitopic sin”.

**References**

1. Bertoni G, Hertig C, Zahno ML, Vogt H-R, Dufour S, Cordano P, Peterhans E, Cheevers WP, Sonigo P, Pancino G. B-cell epitopes of the envelope glycoprotein of caprine arthritis-encephalitis virus and antibody response in infected goats. JGenVirol 2000;81:2929–40.
2. Blatti-Cardinaux L, Pisoni G, Stoffel MH, Zanoni R, Zahno ML, Bertoni G. Generation of a molecular clone of an attenuated lentivirus, a first step in understanding cytopathogenicity and virulence. Virology. 2015;487:50–8.
3. Cardinaux L, Zahno ML, Deubelbeiss M, Zanoni R, Vogt HR, Bertoni G. Virological and phylogenetic Characterisation of attenuated small ruminant lentivirus isolates eluding efficient serological detection. Vet Microbiol. 2013;162:572–81.
4. Mordasini F, Vogt HR, Zahno ML, Maeschli A, Nenci C, Zanoni R, Peterhans E, Bertoni G. Analysis of the antibody response to an immunodominant epitope of the envelope glycoprotein of a lentivirus and its diagnostic potential. J Clin Microbiol. 2006;44:981–91.

#### P68 Impact of immune activation, immune exhaustion, broadly neutralising antibodies and viral reservoirs on disease progression in HIV-infected children

##### Maximilian Muenchhoff^1^, Philip Goulder^2^, Oliver Keppler^1^

###### ^1^Max von Pettenkofer-Institute, Virology, Munich, Germany; ^2^University of Oxford, Paediatrics, Oxford, Great Britain

**Correspondence:** Maximilian Muenchhoff

*Retrovirology* 2016, **13(Suppl 1)**: P70

Paediatric HIV infection is typically characterised by rapid disease progression in the absence of ART. However, there is a subset (5–10 %) of ART-naïve HIV-infected children who remain clinically healthy, maintaining normal-for-age CD4 T-cell counts throughout childhood defined here as ‘paediatric non-progressors’ (PNPs). To identify mechanisms preventing HIV disease progression we studied a cohort of 170 HIV-1 Clade C infected PNPs recruited from multiple clinical sites across South Africa.

In contrast to adults where HIV long-term non-progression is mostly associated with low to undetectable viral load levels, paediatric non-progressors maintained normal-for-age CD4 T-cell counts despite persistently high viremia (median 26,000 HIV copies/ml). T-cell and monocyte activation was increased in progressor children but remained at levels similar to HIV-uninfected children in PNPs. The CD4+ and CD8+ T-cell compartment in progressors, but not PNPs, was differentiated towards pro-inflammatory effector memory phenotypes with high levels of immune exhaustion. HIV specific T-cell responses and potent broadly neutralising antibody (bnAb) responses were detected in both, progressor and non-progressor children (bnAbs in 72 % of paediatric subjects versus 19 % of infected adults, p < 0.0001), but non-progression in paediatric infection was independent of these HIV-specific immune responses.

However, we observed low levels of CCR5 expression and limited HIV infection in long-lived stem cell memory and central memory CD4+ T-cells in paediatric non-progressors in association with higher frequencies of these T-cell subsets (p < 0.0001 vs. progressor controls), suggesting a mechanism contributing to the maintenance of normal-for-age CD4 T-cell counts despite persistently high viremia.

**Fig. 17:**
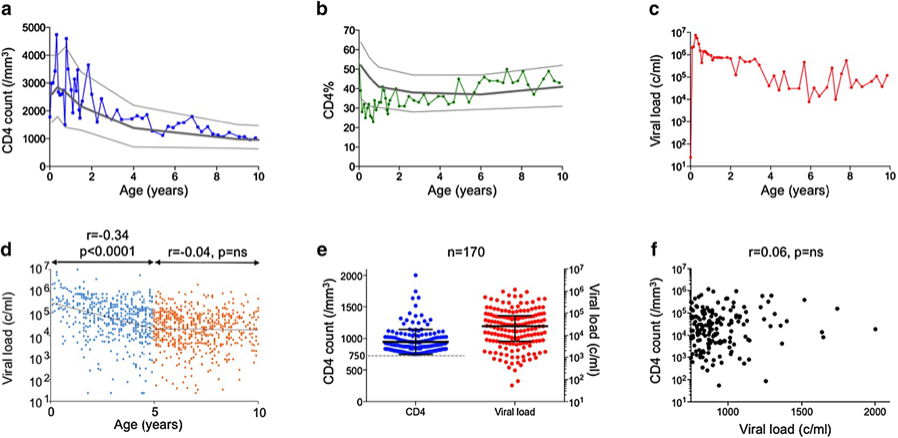
Normal CD4 counts for age and low immune activation despite high viral loads in paediatric non-progressors. **a**–**c** absolute CD4 count, CD4 % and viral load in ART-naïve paediatric subject 517-C over the first 10 years of life. 10th, 50th, and 90th centile of absolute CD4 and CD4% are shown in panels A-B for uninfected children over the first 10 years of life^18–19^. **d** Longitudinal viral load data from 170 cART-naïve paediatric non-progressors. Viral load declines with age over the first 5 years (r = −0.34, p < 0.0001) but then plateaus thereafter. **e** Current absolute CD4 counts and viral loads in 170 paediatric non-progressors. **f** Lack of correlation between CD4 count and viral load in 170 paediatric non-progressors

**Fig. 18:**
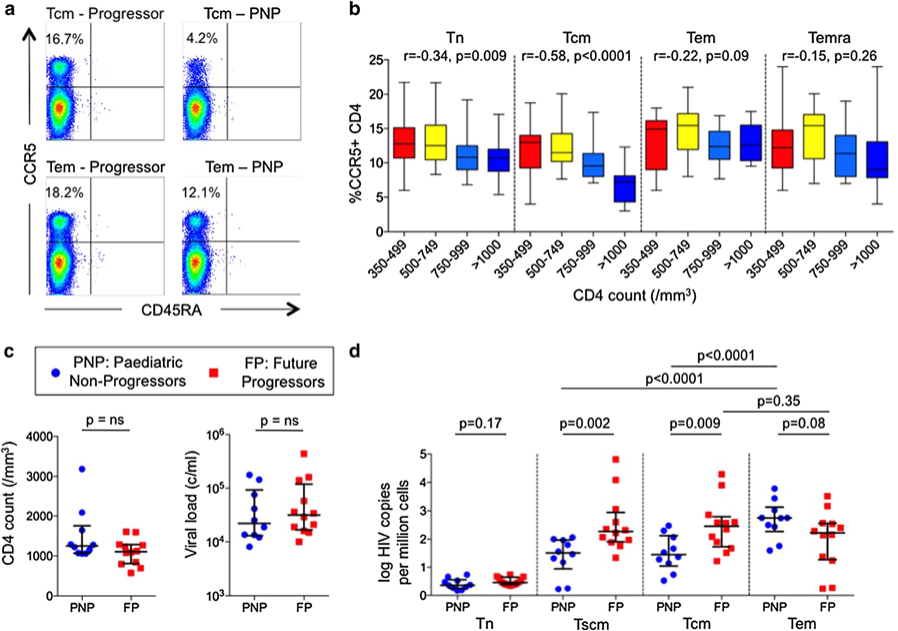
CCR5 expression and HIV infection is lower in central memory CD4+ T-cells in paediatric non-progressors than in progressors. **a** Representative FACS data of CCR5 expression in paediatric progressors versus non-progressors. **b** CCR5 expression on CD4+ T-cell subsets in ART-naive children aged >5 years by absolute CD4 count, **c** Absolute C04 counts and viral loads in paediatric non-progressors and adult future progressors did not differ significantly (median 1251 vs. 1106 cells/mm^J^; and 22,075 vs. 31,500 HIV RNS copies/ml plasma, respectively; similarly CD4% did not differ between the two groups, median 31 versus 36 %, not shown), **d** HIV infection in Tn, Tscm, Tern and Tem in paediatric non-progressors and adult future progressors, determined by qPCR of HIV DNA in sorted CD4 T-cell subsets

These data indicate that the mechanisms of HIV non-pathogenesis in paediatric infection are distinct from those in adults, where disease non-progression is typically linked to viremic suppression in the presence of ‘protective’ HLA class I-mediated immunity; but reminiscent of the natural hosts of SIV infection, such as the sooty mangabey, in which non-pathogenesis is independent of strong virus-specific immunity and characterised by low systemic immune activation despite persistently high viremia (Figs. 17, 18).

### Topic 9: Novel antiviral strategies

#### P69 Identification of natural compounds as new antiviral products by bioassay-guided fractionation

##### Alexandra Herrmann^1^, Stephanie Rebensburg^2^, Markus Helfer^1^, Michael Schindler^3^, Ruth Brack-Werner^1^

###### ^1^Helmholtz Zentrum Munich, Virology, Neuherberg, Germany; ^2^College of Pharmacy/Ohio State University, Columbus, OH, United States; ^3^Institute of Medical Virology and Epidemiology of virus diseases/University Medical Center Tübingen, Tübingen, Germany

**Correspondence:** Alexandra Herrmann

*Retrovirology* 2016, **13(Suppl 1)**: P71

**Introduction:** Medicinal plants are the base of traditional medicine to fight a diversity of infections and diseases for thousands of years. According to estimates, 25 % of the commonly used medicines contain compounds isolated from plants. A variety of herbal products have shown potential to treat a number of viral infections and possess a broad-spectrum antiviral activity.

**Results:** We demonstrate that extracts of *Cistus incanus* (Ci) leaves and roots of *Pelargonium sidoides* inhibit human immunodeficiency virus (HIV) infections in vitro. Antiviral activity was highly selective for virus particles, preventing primary attachment of the virus to the cell surface and viral envelope proteins from binding to heparin. Ci treatment also prevented infection by virus particles containing Ebola and Marburg virus envelope, indicating that antiviral activity of Ci extract extends to emerging viral pathogens. Antiviral activity was mediated by polyphenol-enriched fraction. Using Bioassay-guided fractionation of whole Ci extracts, we detected the presence of numerous, separable antiviral compounds and we were also able to identify the structure of several components with anti-HIV activity.

**Conclusion:** These results demonstrate a potent and broad in vitro antiviral activity of leaf extracts of Ci against potentially lethal viruses for humans and also highlight medicinal plants as a promising source of novel antiviral agents.

#### P70 The PPARG antagonism disconnects the HIV replication and effector functions in Th17 cells

##### Yuwei Zhang^1^, Huicheng Chen^1^, Delphine Planas^1^, Annie Bernier^1^, Annie Gosselin^1^, Jean-Pierre Routy^2^, Petronela Ancuta^1^

###### ^1^CRCHUM, montreal, Canada; ^2^McGill University Health Centre, Montreal, Canada

**Correspondence:** Yuwei Zhang

*Retrovirology* 2016, **13(Suppl 1)**: P72

**Question:** Mucosal Th17-polarized CD4+ T-cells are the first targets of HIV/SIV infection and contribute to HIV persistence during ART. PPARγ agonists negatively regulate RORγt-mediated Th17 functions and block HIV replication. Here, we investigated the potential use of PPARγ antagonism for viral reactivation and Th17 restoration during HIV infection.

**Methods:** Memory CD4+ T cells were isolated from PBMCs of HIV− or HIV+ on ART individuals using magnetic beads (Miltenyi) and stimulated with anti-CD3/CD28 Abs. HIV− cells exposed to replication-competent or VSVG-pseudotyped HIV in vitro and cells isolated from HIV+ on ART individuals were cultured in the presence or absence of the PPARγ agonist Rosiglitazone (RGZ) or antagonist T0070907 (T007) for 12 days. HIV-p24 and IL-17A levels were quantified by ELISA and FACS. HIV-DNA integration was quantified by real-time PCR. Gene expression was quantified by real time RT-PCR. The expression of HIV co-receptors CCR5 and CXCR4 was measured by FACS.

**Results:** RGZ decreased both HIV replication and IL-17A production. As predicted, T007 increased IL-17A expression/production. Surprisingly, T007 alone or in combination with RGZ inhibited HIV replication by reducing CCR5 expression and HIV transcription. The T007-mediated effects coincided with the induction of cholesterol-25-hydroxylase, an enzyme converting cholesterol to 25-hydroxycholesterol, a molecule known to broadly inhibit viral infection and recently identified as an intrinsic agonist of RORγt. Finally, T007 inhibited HIV reactivation and promoted IL-17A production in CD4+ T cells from HIV+ on ART individuals.

**Conclusions:** We demonstrated that PPARγ antagonism interferes with HIV replication/reactivation while promoting the Th17 effector functions at least in part via the synthesis of 25-hydroxycholesterol. Future studies in animal models and human clinical trials should determine whether PPARγ is an appropriate target for viral eradication and immune restoration.

#### P71 Characterisation of a multiresistant subtype AG reverse transcriptase: AZT resistance, sensitivity to RNase H inhibitors and inhibitor binding

##### Birgitta Wöhrl^1^, Anna Schneider^1^, Angela Corona^2^, Imke Spöring^3^, Mareike Jordan ^1^, Bernd Buchholz^4^, Elias Maccioni^2^, Roberto Di Santo^5^, Jochen Bodem ^3^, Enzo Tramontano^2^, Kristian Schweimer^1^

###### ^1^University Bayreuth, Biopolymere, Bayreuth, Germany; ^2^University of Cagliari, Life and Environmental Sciences, Monserrato Cagliari, Italy; ^3^University Würzburg, Virology and Immunbiology, Würzburg, Germany; ^4^University Heidelberg, Medical Institution Mannheim, Heidelberg, Germany; ^5^University of Rome, Rome, Italy

**Correspondence:** Birgitta Wöhrl

*Retrovirology* 2016, **13(Suppl 1)**: P73

We analysed a multiresistant (MR) HIV-1 reverse transcriptase (RT), subcloned from a patient-derived subtype CRF02_AG, harboring 45 amino acid exchanges, amongst them four thymidine analog mutations (TAMs) relevant for AZTMP excision (M41L, D67N, T215Y, K219E, lacking K70R) as well as four substitutions of the AZTTP discrimination pathway (A62V, V75I, F116Y and Q151M, lacking F77I). In addition, K65R, known to antagonize AZTMP excision in HIV-1 subtype B was present. Although MR-RT harbored the most significant exchanges T215Y and Q151M of each pathway, it exclusively used AZTTP discrimination. This indicates that the two mechanisms are mutually exclusive and that the Q151M pathway is preferred since it confers resistance to most nucleoside inhibitors. A derivative was created, additionally harboring the TAM K70R and the reversions M151Q as well as R65K since K65R antagonizes excision. MR-R65K-K70R-M151Q was able of AZTMP excision, whereas other combinations thereof with only one or two exchanges still promoted discrimination. Furthermore, several amino acid substitutions present in the ribonuclease H domain correlate with TAMs, thus making RNase H a suitable target for inhibitors. All MR-RTs exhibited similar sensitivity towards RNase H inhibitors belonging to different inhibitor classes, indicating the importance of developing RNase H inhibitors further as anti-HIV drugs. Using NMR spectroscopy, we were able to identify the binding pocket in the HIV-1 RNase H for one of the RNase H inhibitors.

#### P72 Insigths into the acetylation pattern of HDAC inhibitors and their potential role in HIV therapy

##### Christian Schölz^1^, Brian Weinert^2^, Sebastian Wagner^2^, Petra Beli^2^, Yasuyuki Miyake^3^, Jun Qi^4^, Lars Jensen^2^, Werner Streicher^2^, Anna McCarthy^5^, Nicholas Westwood^6^, Sonia Lain^5^, Jürgen Cox^7^, Patrick Matthias^3^, Matthias Mann^7^, James Bradner^4^, Chunaram Choudhary^2^

###### ^1^Max-von-Pettenkofer Institute; LMU, Virology, Munich, Germany; ^2^NNF CPR; University Copenhagen, Copenhagen, Denmark; ^3^Friedrich Miescher Institute, Basel, Switzerland; ^4^Dana-Farber Cancer Institute, Harvard Medical School, Boston, MA, United States; ^5^Karolinska Institutet, Stockholm, Sweden; ^6^EaStCHEM, St. Andrews, Great Britain; ^7^Max Planck Institute for Biochemistry, Martinsried, Germany

**Correspondence:** Christian Schölz

*Retrovirology* 2016, **13(Suppl 1)**: P74

HIV latency, e.g. in resting CD4^+^ T-cells, is the cardinal obstacle for eradicating the virus and curing an infected individual. Antiretroviral therapy (ART) is extremely effective in suppressing viral replication, yet fails to effectively diminish latent, transcriptionally inactive cellular reservoirs of HIV. Remarkably, very recently it has been demonstrated that inhibition of histone deacetylases by chemical compounds is sufficient to reactivate HIV and thus to unmask and sensitize a proportion of latently infected cells for killing. These lysine deacetylase inhibitors (KDACIs) are used in basic research, and many are being investigated in clinical trials for treatment of cancer and other diseases like HIV. However, their specificities in cells are incompletely characterised. Here we used quantitative mass spectrometry (MS) to obtain acetylation signatures for 19 different KDACIs, covering all 18 human lysine deacetylases. Most KDACIs increased acetylation of a small, specific subset of the acetylome, including sites on histones and other chromatin-associated proteins. Inhibitor treatment combined with genetic deletion showed that the effects of the pan-sirtuin inhibitor nicotinamide are primarily mediated by SIRT1 inhibition. Furthermore, we confirmed that the effects of tubacin and bufexamac on cytoplasmic proteins result from inhibition of HDAC6. Bufexamac also triggered an HDAC6-independent, hypoxia-like response by stabilizing HIF1-α, providing a possible mechanistic explanation of its adverse, pro-inflammatory effects. Our results offer a systems view of KDACI specificities, providing a profound framework for the understanding of their effect on latently infected cells and will support future investigations towards a combined ART/KDACI therapy.

#### P73 HPV-derived and seminal amyloid peptides enhance HIV-1 infection and impair the efficacy of broadly neutralising antibodies and antiretroviral drugs

##### Marcel Stern^1,2,3^, Oliver T. Keppler^1,2,3^

###### ^1^LMU, Max von Pettenkofer Institute, Virology, Munich, Germany; ^2^German Center for Infection Research, Munich, Germany; ^3^University Hospital Frankfurt, Institute of Medical Virology, Frankfurt a. M., Germany

**Correspondence:** Marcel Stern

*Retrovirology* 2016, **13(Suppl 1)**: P75

SEVI (*seminal enhancer of virus infection*) forms amyloid fibrils from fragments of the abundant semen marker prostatic acidic phosphatase in human seminal fluid and enhances HIV infection (Münch et al., Cell, 2007). We recently identified the ability of an abundant cleavage product of the E4 protein of mucotropic HPV types ((Δ1–17)E4) (thereafter referred to as E4) to assemble into cationic, intermediate amyloid fibrils that can capture and concentrate HIV particles, protecting virion infectivity and drastically promoting fusion to primary HIV target cells. Thus, the concept emerges that combinations of aggregating cleavage products originating from body fluids and from co-infecting pathogens may enhance the likelihood of HIV transmission in the anogenital tract. Besides antiretroviral (ART) treatment, protection from sexual HIV transmission by broadly neutralising antibodies (bNAbs) could in principle be accomplished by active immunisation with a potent vaccine or, as has been demonstrated in animal models, by passive immunisation with bNAbs.

In the present study we explored the capacity of three potent human second generation bNAbs, targeting either the CD4 binding site or spanning several epitopes in Env, as well as binding and entry inhibitors to block infection of primary CD4 T-cells in the context of infection-enhancing amyloids, SEVI or E4 from HPV16. We observed that the relative and absolute efficacy of drugs and bNAbs was diminished to variable degrees in the presence of the infection enhancers with increases of IC50 concentrations ranging from 2- to 30-fold (drugs) and 2- to 16-fold (bNAbs), respectively. At concentrations of drugs or bNAbs, which reduced control infection levels to background, infections in the presence of the amyloid peptides were elevated by 4- to 26-fold (drugs) or 7- to 54-fold (bNAbs). At high bNAb concentrations the most potent bNAbs and all drugs tested were capable of completely inhibiting infection even when SEVI or E4 were present. Taken together and in line with a recent report (Zirafi et al., Sci. Transl. Med., 2014), amyloid fibrils that are present at the anogenital tract can undermine the inhibitory effect of antiretroviral drugs and the neutralising capacity of bNAbs. These types of studies may impact the drug and bNAb candidate selection and may help to define desirable target concentrations in the anogenital mucosa for the prevention of sexual transmission of HIV.

#### P74 D(−)lentiginosine inhibits both proliferation and virus expression in cells infected by HTLV-1 in vitro

##### Elena Valletta^1^, Caterina Frezza^1^, Claudia Matteucci^2^, Francesca Marino-Merlo^3^, Sandro Grelli^2^, Anna Lucia Serafino^4^, Antonio Mastino^3,4^, Beatrice Macchi^1,2^

###### ^1^University of Rome Tor Vergata, Systems Medicine, Rome, Italy; ^2^University of Rome Tor Vergata, Experimental Medicine and Surgery, Rome, Italy; ^3^University of Messina, Chemical, Biological, Pharmaceutical and Environmental Sciences, Messina, Italy; ^4^CNR, The Institute of Translational Pharmacology, Rome, Italy

**Correspondence:** Beatrice Macchi

*Retrovirology* 2016, **13(Suppl 1)**: P76

**Question:** (−)lentiginosine (-LENT) is a non-natural iminosugar showing glycosidase inhibitor properties. We have recently shown that -LENT exerts a preferential pro-apoptotic activity towards tumor cells in comparison with non-tumor cells.

**Methods:** The effects of -LENT on PBMC from healthy donors infected in vitro with HTLV-1 by co-culture with irradiated MT-2 or C91/PL cells, were then evaluated through real time PCR and flow cytometry analysis.

**Results:** LENT inhibited HTLV-1 expression in a dose-dependent fashion, at concentrations of 15, 10 and 5 µM, in HTLV-1 infected cells. Proliferation of MT-2, C91/PL and HTLV-1-immortalized IL-2-dependent CD4+ cells (CD4/HTLV-1) were inhibited, in comparison with chemotherapeutic agent, after 24 h of treatment, with an IC_50_ of 327, 113 and 131 µM, respectively, while stimulated PBMC were inhibited with an IC_50_ of 170 µM. Conversely, cell growth of HTLV-1 infected cell lines was more efficiently inhibited after 48–72 h of treatment, with cytotoxic concentrations 50 % (CC_50_) of 50 and 70 µM for MT-2, C91/PL and CD4/HTLV-1, respectively. In addition, cell growth of HTLV-1 infected cell lines was more efficiently inhibited after long-term treatment with -LENT at a concentration of 5 µM in comparison with stimulated PBMC and 5 µM AZT-treated HTLV-1-infected cells. Moreover, confocal microscopy studies and flow cytometry analysis showed that treatment for 4 h with 20 µM -LENT inhibited by 50 % GLUT-1 receptor expression. Actually, GLUT-1 was differently distributed in the cytosol of -LENT-treated cells in comparison with untreated cells.

**Conclusions:** These data suggest that -LENT could interfere with glucose metabolism in HTLV-1 infected/transformed cells, causing a preferential inhibition of cell growth in these cells. The use of metabolic inhibitors, in combination or not with other agents, is an interesting, potential novel strategy against HTLV-1 associated pathologies. Further studies are necessary to verify this hypothesis.

#### **P75** HIV-1 resistance analyses of the Cape Winelands districts, South Africa

##### Sello Mikasi, Graeme Jacobs, Susan Engelbrecht

###### Stellenbosch University, Pathology, Cape Town, South Africa

**Correspondence:** Sello Mikasi

*Retrovirology* 2016, **13(Suppl 1)**: P78

**Background:** South Africa remains the leading country highly affected by HIV/AIDS, with 6.8 million people living with the disease and at least 3.1 million people on ARV(UNAIDS 2015). With the scale-up of ARV programme in the country a pragmatic approach to ART programme in monitoring and evaluation was developed in the Western Cape Province of South Africa.In this study we investigated the change in genotypic drug resistance of the Reverse *Transcriptase* (PR) region from our viral load monitoring cohort.

**Methods:** We analysed the HIV-1 associated drug resistance mutations in plasma samples submitted to the Tygerberg Academic Hospital National Health Service Laboratoryfor HIV-1 Viral load monitoring. Our 205 cohort samples with a viral load above 2000 copies/ml were amplified by PCR and sequenced. Viral subtyping was done using online tools and drug resistannce mutations were screened using the Stanford University HIV Drug Resistance Database for Interpretation and the International AIDS Society-USA Guidelines

**Results:** We detected resistance associated mutations against RT inhibitors in 63.5 % of samples analysed. This includes 34 NRTI mutations (33.9 %) and 71 NNRTI mutations (61.7 %). In addition 93.1 % of the virus is subtype C With 6.9 % of other non-C subtypes detected A(1.7 %) and B(5.2 %) respectively.

**Discussion:** As the ARV programme is scaling up in the country, it is essential to monitor and evaluate the resistance patterns of HIV-1. Our results shows that majority of the HIV/AIDS population around the Western Cape are acquiring drug resistance mutation. Our current results reflect that most patients harbourmutations that confer resistance to first-line ARV therapy given the highest number of RT mutations detected in this study.

**Reference**

1. UNAIDS. UNAIDS World AIDS Day Report. 2015.

### Topic 10: Recent advances in HIV vaccine development

#### P76 Induction of complex retrovirus antigen-specific immune responses by adenovirus-based vectors depends on the order of vector administration

##### Meike Kaulfuß, Sonja Windmann, Wibke Bayer

###### University Hospital Essen, Institute for Virology, Essen, Germany

**Correspondence:** Wibke Bayer

*Retrovirology* 2016, **13(Suppl 1)**: P77

In the Friend retrovirus mouse model we developed potent adenovirus-based vaccines that were designed to induce strong Friend virus-specific CD8^+^ T cell or antibody responses, respectively. In order to create an optimal vaccine, we pursued a combination vaccination protocol.

While the vectors on their own confer strong protection from a subsequent Friend virus challenge, the simple combination of the vectors for the establishment of an optimized immunisation protocol did not result in a further improvement of vaccine effectivity. In fact, we found that the co-immunisation of a CD8^+^ T cell-inducing Leader-Gag vector with Envelope-encoding vectors abrogated the induction of GagL_85–93_-specific CD8^+^ T cells, even if the two vaccines were spatially separated. In a successive immunisation approach, we found that the order of vector administration was crucial for the vaccination outcome, as the immunisation with the CD8^+^ T cell inducing vector had to precede the immunisation with an Envelope encoding vector for the efficient induction of CD8^+^ T cells, which would otherwise be suppressed, whereas the antibody response to Envelope was in fact enhanced when the mice were adenovirus-experienced from a prior immunisation. Using a rational, two immunisations-based vaccination protocol we established an adenovirus-based vaccine regimen that induces potent immune responses and confers strong protection of highly Friend virus-susceptible mice from a lethal Friend virus challenge. The degree of protection from the high-dose challenge FV infection mediated by the optimized vector-based immunisation was comparable to the level of protection conferred by an attenuated retrovirus immunisation, which is considered by many the gold standard vector-based vaccines have to meet.

Our data highlights the importance to consider the interplay of vaccine antigens in simultaneous as well as consecutive immunisations, and demonstrates the potential of vector-based immunisation approaches.

#### P77 Direct impact of structural properties of HIV-1 Env on the regulation of the humoral immune response

##### Rebecca Heß^1^, Michael Storcksdieck gen. Bonsmann^1^, Viktoria Stab^1^, Carsten Kirschning^2^, Bernd Lepenies^3^, Matthias Tenbusch^1^, Klaus Überla^4^

###### ^1^Ruhr-University Bochum, Molecular and Medical Virology, Bochum, Germany; ^2^University Hospital Essen, Medical Microbiology, Essen, Germany; ^3^University of Veterinary Medicine Foundation, Infection Immunology, Hanover, Germany; ^4^Universitätsklinikum Erlangen, Clinical and Molecular Virology, Erlangen, Germany

**Correspondence:** Rebecca Heß

*Retrovirology* 2016, **13(Suppl 1)**: P79

So far there have been six HIV vaccine efficacy trials, but only the RV144 trial provided evidence for some level of protection. The results of the RV144 trial showed that protection correlated with a higher induction of IgG3 which is the most potent antibody (AB) subclass in human with regard to Fc-mediated immune functions like the Antibody-dependent cell-mediated cytotoxicity (ADCC). Thus, it seems to be important which AB subclass can be induced by a vaccine.

In our previous immunisation experiments, we observed a bias in the HIV-1 Env-specific AB response in mice. Upon DNA vaccination with Env, mice tend to produce more IgG1 than IgG2a while vaccination with structurally comparable viral surface proteins like the Hemagglutinin of the Influenza A Virus (IAV-HA) or the Fusion protein of the Respiratoral Syncytial Virus (RSV-F) results in a balanced response. In mice, IgG2a has the highest ability to induce Fc-mediated effector functions, therefore we want to know which mechanism leads to the differential immune response.

Vaccination with plasmids encoding soluble versions of HIV-Env, IAV-HA, and RSV-F fused to the HIV-1 core protein p24 showed that the bias in the Env specific antibody response was transferred to the p24 specific AB response. The surplus of IgG1 was accompanied by raised levels of the Th2 cytokines IL-5 and IL-13. Immunisations of knockout mice for MyD88 and TRIF, or Card9 respectively, led as well to a biased response suggesting that the bias is neither dependent on Toll Like Receptor (TLR) signaling nor the signaling of C-type lectin receptors (CLR). Interestingly, vaccination with plasmids encoding truncated versions of Env revealed that the bias vanishes when the protein is shortened as far as C1-V1V2. A longer version including additionally C2-V3 still led to a bias indicating that the unbalanced response is due to a feature of the C2-V3 domain of the protein.

Taken together, our results indicate that Env is able to modulate the humoral part of the adaptive immune response via a protein immanent feature within the C2-V3 domain. Further analysis of this protein part might improve our knowledge about the underlying mechanism of the differentially regulated immune response.

#### P78 Lentiviral virus-like particles mediate gerenration of T-follicular helper cells in vitro

##### Anne Kolenbrander^1^, Klaus Überla^2^, Vladimir Temchura^2^

###### ^1^Ruhr-University Bochum, Department of Molecular and Medical Virology, Bochum, Germany; ^2^Institute of Clinical and Molecular Virology, Erlangen, Germany

**Correspondence:** Vladimir Temchura

*Retrovirology* 2016, **13(Suppl 1)**: P80

T-follicular helper cells (TFH) play a central role in the formation and maintenance of germinal centers and the establishment of long-lived antibody responses. The understanding of TFH induction and development during immune responses is crucial to build up novel vaccination strategies. As a B-cell targeting antigen-delivery system, virus-like particles (VLP) are able to provide several unique direct effects on the cognate B-cells that cannot be achieved by the monovalent form of the antigen. In co-cultures of transgenic T- and B-cells, cognate VLPs efficiently induced co-expression of TFH-master regulator transcription factor BCL-6 together with follicular marker CXCR5 in up to 40 % of the CD4^+^ T-cells. Production of IL-21 and isotype switching of the B-cells to IgG1 further indicated helper functions of the generated BCL-6^+^ CXCR5^+^ T-cells. Our study confirms the importance of the cognate B- and T-cell cross-talk for the TFH-differentiation process. Presented robust system to generate TFH cells in vitro, based on natural biological background and minimal additional requirements is relevant for both basic scientific research on TFH cell biology and rational vaccine design.

#### P79 Recruitment of HIV-1 Vpr to DNA damage sites and protection of proviral DNA from nuclease activity

##### Kenta Iijima^1^, Junya Kobayashi^2^, Yukihito Ishizaka^1^

###### ^1^National Center for Global Health and Medicine, Department of Intractable Diseases, Research Institute, Tokyo, Japan; ^2^Kyoto University, Department of Genome Repair Dynamics, Radiation Biology Center, Kyoto, Japan

**Correspondence:** Yukihito Ishizaka

*Retrovirology* 2016, **13(Suppl 1)**: P81

There are lines of evidence that the infection of human immunodeficiency virus ype-1 (HIV) causes DNA double strand break (DSB). We reported that HIV integrates in the DSB sites without catalytic activity of HIV integrase (IN) [1]. Here we found that Vpr is recruited to DSB sites and protects proviral DNA from nuclease activity during viral integration into the host genome.

We first observed that Vpr is recruited to DSB sites by FRAP assay, in which Vpr was accumulated to DSB tracks that were created by micro-irradiation (μ-IR). The ChIP assay revealed similar results that Vpr was accumulated to the *DAB*1 locus after expression of I-*Ppo* I enzyme, a rare-cutting endonuclease that recognizes nucleotide sequence of 15 base pairs, the target site of which is present in the *DAB1* locus. Interestingly, the recruitment of Vpr to the DSB sites was observed even under the presence of inhibitors of ATM and DNA-PKcs, central factors of DSB signaling. Data suggested that the DSB sensor proteins are involved in the early response of Vpr to the DSB sites. To identify a responsive cellular factor, we performed μ-IR experiments in a patient derived cell line, in which Mre11, a component of a DSB sensor complex is deficient (*∆*Mre11 cells). Interestingly, the recruitment of Vpr to μ-IR created DSB sites was completely abolished in the *∆*Mre11 cell, whereas it was restored by complementation of *Mre11* gene product in the cells. We next compared data of nucleotide sequence of the proviral DNA-ends after infection with two types of IN-activity defective HIV viruses that were proficient or deficient of Vpr. Interestingly, the proviral DNA-ends sequence from Vpr deficient virus was susceptible to larger deletion compared to that of proficient virus, implying that Vpr protected viral DNA-ends during the IN-independent integration process. Given that Mre11 and Vpr are physically associated and Mre11 is a nuclease that functions in DSB repair by non-homologous end-joining (NHEJ), data suggested that Vpr negatively regulates the nuclease activity of Mre11 protein, which was confirmed by in vitro nuclease assay. Finally, we tested the inhibitory effect of Mre11 on the HIV infection, and several independent experiments revealed that Mre11 inhibited HIV infection, implying that Mre11 is a novel restriction factor against HIV infection. Notably, the Mre11-mediated suppression of viral infection was not restored by Vpr, implying that Mre11 has multiple roles in HIV infection. Taken together with our observation that Vpr induces DNA damage in resting macrophages, we propose that Vpr is an important factor that positively functions in viral infection into resting macrophages, especially in the presence of integrase inhibitor.

**Reference**

1. Koyama T, Sun B, Tokunaga K, Tatsumi M, Ishizaka Y. DNA damage aids human immunodeficiency virus type 1 infection by overcoming integrase inhibition. Retrovirology. 2013;10:21.

